# Early Dose Reduction or Discontinuation vs Maintenance Antipsychotics After First Psychotic Episode Remission: A Randomized Clinical Trial

**DOI:** 10.1001/jamapsychiatry.2025.2525

**Published:** 2025-10-01

**Authors:** Iris E. Sommer, Franciska de Beer, Shiral Gangadin, Lieuwe de Haan, Wim Veling, Nico van Beveren, Nynke Boonstra, Bram-Sieben Rosema, Jim van Os, Martijn Kikkert, Sanne Koops, Jort Noorman, Frederick Thielen, Ben Wijnen, Marieke Begemann

**Affiliations:** 1 Center for Clinical Neuroscience and Cognition, University of Groningen, University Medical Center Groningen, Groningen, the Netherlands; 2 Department of Early Psychosis, Amsterdam University Medical Center, Academic Medical Center, Amsterdam, the Netherlands; 3 Department of Psychiatry, University of Groningen, University Medical Center Groningen, Groningen, the Netherlands; 4 Department of Psychiatry, University Medical Center Utrecht Brain Center, University Medical Center Utrecht, Utrecht, the Netherlands; 5 Department of Psychiatry and Neuropsychology, School for Mental Health and Neuroscience, Maastricht University Medical Centre, Maastricht, the Netherlands; 6 King’s Health Partners Department of Psychosis Studies, King’s College London, London, United Kingdom; 7 Antes Center for Mental Health Care, Rotterdam, the Netherlands; 8 Department of Neuroscience, Erasmus Medical Center, Rotterdam, the Netherlands; 9 NHL Stenden, University of Applied Sciences, Leeuwarden, the Netherlands; 10 KieN VIP Mental Health Care Services, Leeuwarden, the Netherlands; 11 Department of Research, Arkin Mental Health Care, Amsterdam, the Netherlands; 12 Centre of Economic Evaluation & Machine Learning, Trimbos Institute (Netherlands Institute of Mental Health), Utrecht, the Netherlands; 13 Institute of Psychiatry, Psychology & Neuroscience, London, United Kingdom

## Abstract

**Importance:**

Dose reduction or discontinuation (DRD) early after remission from first-episode psychosis (FEP) increases short-term relapse risk. Controversy remains regarding potential benefits in functioning over the longer term because studies with long-term outcomes show conflicting findings.

**Objective:**

To compare short- and long-term effects between DRD and maintenance medication over a 4-year period in a large sample of patients with FEP.

**Design, Setting, and Participants:**

The Handling Antipsychotic Medication Long-Term Evaluation of Targeted Treatment (HAMLETT) study is a single-blind pragmatic randomized (1:1) clinical trial conducted in 26 specialized psychosis units in the Netherlands from September 2017 to March 2023. Patients remitted for FEP from in- and outpatient services were included.

**Interventions:**

DRD within 12 months after remission compared with 12 months maintenance treatment.

**Main Outcomes and Measures:**

The primary outcome was patient-rated functioning, measured by the World Health Organization Disability Assessment Schedule 2.0 (WHODAS-2). Secondary outcomes were researcher-rated global assessment of functioning (GAF), quality of life, relapse, symptom severity (measured by the Positive and Negative Syndrome Scale [PANSS]), serious adverse events, and adverse effects.

**Results:**

A total of 347 patients (241 male [69.5%]; mean [SD] age, 27.9 [8.7] years) were included, with 168 randomized to early DRD and 179 to maintenance. WHODAS-2 showed no time × condition interaction. In the first year, DRD was associated with higher risk of relapse (odds ratio, 2.84; 95% CI, 1.08 to 7.66; *P* = .04) and lower quality of life (β = −3.31; 95% CI, −6.34 to −0.29; *P* = .03). At 3 years (β = 3.61; 95% CI, 0.28 to 6.95; *P* = .03) and 4 years (β = 6.13; 95% CI, 2.03 to 10.22; *P* = .003), a nonlinear effect of time occurred, showing significantly better GAF for patients in the DRD condition, with a similar trend for PANSS at 4 years (*P *for trend = .06). Although SAEs and adverse effects were similar between groups, 3 confirmed deaths by suicide occurred in the DRD group, against 1 death by suicide in the maintenance group.

**Conclusions and Relevance:**

This randomized clinical trial found that DRD posed risks of relapse and worse quality of life over the first year but yielded better researcher-rated functioning at the third and fourth year, with a similar trend for symptom severity; because antipsychotic medication doses were comparable in the 2 groups from 1 year onwards, this finding is not a direct result of lower medication but may reflect a learning experience to use antipsychotics to better handle psychotic vulnerability. These findings suggest that the potential learning and empowering element of DRD needs to be weighed carefully against short-term risks.

**Trial registration:**

EudraCT number: 2017-002406-12.

## Key Points

**Question:**

What are the short-term effects and long-term effects of early dose reduction or discontinuation (DRD) compared with maintenance treatment for patients remitted from first-episode psychosis?

**Findings:**

This randomized clinical trial of 347 patients with first-episode psychosis found no difference in patient-rated functioning between conditions; in the short term, those who received DRD had higher risk of relapse and lower quality of life. From 3 years onwards, DRD showed better researcher-rated functioning, with a similar trend for symptom severity.

**Meaning:**

These findings suggest that because medication was similar from 12 months onwards, better longer-term functioning could reflect the learning experience of a guided tapering attempt, rather than direct medication effects.

## Teaser

This randomized clinical trial compares early dose reduction or discontinuation vs maintenance treatment of antipsychotic medication for patients remitted from first-episode psychosis in the Netherlands.

## Introduction

After remission of first-episode psychosis (FEP), many patients have the desire to taper off antipsychotic medication, yet guidelines recommend continuation of medication for 1 to 2 years after FEP because relapse risk increases steeply on discontinuation, even with gradual tapering.^1^ In reality, 70% of antipsychotic users discontinue medication within the first year,^2^ often without clinical supervision.

Wunderlink et al^3^ randomized 128 patients with FEP to open label dose reduction or discontinuation (DRD) vs maintenance, finding no difference in functioning with more relapses after 18 months. Reevaluation 5 years later showed functional recovery of 40.4% after DRD against 17.6% after maintenance.^4^ Chen et al^5^ randomized 178 patients with FEP to 400 mg of quetiapine or placebo in a double-blind trial and recontacted patients after 10 years. In the discontinuation group, 39% had a poor outcome, opposed to 21% in the maintenance group.^6^ Neither study preregistered these long-term outcomes. In this current study, to provide conclusive evidence regarding long-term effects of early DRD in patients with FEP, 26 Dutch psychosis centers included a large FEP sample followed for 4 years.

## Methods

This randomized clinical trial was approved by the ethics committee of the University Medical Center Groningen and was preregistered (EudraCT 2017-002406-12). Reporting follows the Consolidated Standards of Reporting Trials (CONSORT) reporting guideline, and the trial protocol and statistical analysis plan is available in Supplement 1. All participants provided written informed consent and were included between September 2017 and March 2023. Included participants used antipsychotic medication; had achieved symptomatic remission for 3 to 6 months; and had a *DSM-5* diagnosis of FEP schizophrenia, schizoaffective disorder, schizophreniform disorder, brief psychotic disorder, or unspecified schizophrenia spectrum and other psychotic disorder. Exclusion criteria were dangerous behavior during FEP or coercive treatment.

Participants were randomized 1:1 to maintenance (≤25% dose reduction) or DRD (gradual hyperbolic tapering with minimal dose reduction of 25% until dose 0 or until symptom return) (eAppendices 1-3 in Supplement 2). The intervention period lasted 6 months, with a follow-up of 48 months. When patients did not follow the randomized condition, they remained in the study.

### Outcomes

The main outcome was patient-rated personal and social functioning, assessed with the World Health Organization Disability Assessment Schedule 2.0 (WHODAS-2; 36 items).^7^ Secondary outcomes included interviewer-rated global assessment of functioning (GAF), symptom severity (Positive and Negative Syndrome Scale [PANSS]),^8^ and health-related quality of life (measured using the Visual Analog Scale of the EuroQo 5-Dimension Health-Related Quality of Life scale [EQ-5D-VAS]).^9^ Relapse was defined according to Siafis et al^10^ with the addition of hospitalization for psychosis and relapse as explicitly reported by the treating clinician. Safety and tolerability outcomes were adverse events (AEs), serious AEs (SAEs), self-harm, aggressive behavior, police contact, adverse effects, and body mass index (BMI).

### Statistical Analysis

Data were primary analyzed according to the intention-to-treat principle, applying generalized linear mixed modeling (continuous outcomes) and logistic models (binary outcomes), with random effects for repeated assessments nested in clinical teams and fixed effects for condition, time, and condition × time. Data were also analyzed per protocol. Subanalyses for sex at birth and for only patients with schizophrenia or schizoaffective disorder were performed. All analyses were performed with baseline score as a separate covariate to examine the impact of baseline imbalances. Analyses were conducted using R 4.3.2 (R Project for Statistical Computing) via RStudio version 2023.12.1.402 (Posit), and a 2-sided *P* < .05 was considered significant.

## Results

A total of 453 patients signed informed consent, and 347 patients were included in the analysis (mean [SD] age, 27.9 [8.7] years; 241 male [69.5%]) and randomized to DRD (168 patients) or maintenance (179 patients) (eAppendix 4 in Supplement 2). Baseline demographics are provided in Table 1, and medication characteristics are in eAppendix 5 in Supplement 2. At the end of the intervention period, mean (SD) olanzapine equivalents were 3.8 (7.5) mg per day for DRD and 6.9 (6.1) mg per day for maintenance. In the DRD group, 110 participants (65.5%) had completely discontinued compared with 49 participants (27.4%) for maintenance. There were 133 patients (79.2%) in the DRD group and 108 patients (60.3%) in the maintenance group who adhered to protocol. For GAF and PANSS scores, a nonlinear effect of time was shown, with lines crossing at 24 months for GAF and at 36 months for PANSS, indicating better outcomes in the DRD condition from 3 years onwards (Figure). Mean (SD) outcome measures per condition and timepoint linear mixed-effects models for primary and secondary outcomes are shown in eAppendix 6 in Supplement 2. Significant interactions were found for EQ-5D-VAS at 6 months (worse outcome for DRD: β = −3.31; 95% CI, −6.34 to −0.29; *P* = .03), proportion relapsed at 12 months (worse for DRD: odds ratio, 2.84; 95% CI, 1.08 to 7.66; *P* = .04), GAF at 36 months (better outcome for DRD: β = 3.61; 95% CI, 0.28 to 6.95; *P* = .03), and GAF 48 months better outcome for DRD: (β = 6.13; 95% CI, 2.03 to 10.22; *P* = .003) (Table 2). Symptoms severity showed a similar trend towards better outcome for DRD (PANSS) at 48 months (β = −3.02; 95% CI, −6.17 to 0.13; *P* = .06).

**Table 1.** Baseline Demographic and Clinical Characteristics

Characteristic	Participants, No. (%)		
	Dose reduction or discontinuation (n = 168)	Maintenance (n = 179)	Overall (N = 347)
Age, mean (SD), y	27.9 (8.2)	27.9 (9.1)	27.9 (8.7)
Sex at birth			
Male	119 (70.8)	122 (68.2)	241 (69.5)
Female	49 (29.2)	57 (31.8)	106 (30.5)
*DSM-5* diagnosis			
295.40 Schizophreniform disorder	45 (26.8)	49 (27.4)	94 (27.1)
295.70 Schizoaffective disorder	35 (20.8)	46 (25.7)	81 (23.3)
295.8 Brief psychotic disorder	6 (3.6)	8 (4.5)	14 (4.0)
295.90 Schizophrenia	81 (48.2)	76 (42.5)	157 (45.2)
298.9 Unspecified schizophrenia spectrum and other psychotic disorder	1 (0.6)	0	1 (0.3)
Years of education, mean (SD)	14.2 (2.4)	13.8 (2.5)	14.0 (2.5)
World Health Organization Disability Schedule 2.0, mean (SD)	57.4 (18.8)	52.3 (15.4)	54.8 (17.3)
Global assessment of functioning, mean (SD)	64.2 (12.4)	65.5 (11.4)	64.8 (11.9)
Positive and Negative Syndrome Scale, mean (SD)	45.5 (10.3)	44.0 (9.7)	44.7 (10.0)
EQ-5D-VAS, mean (SD)	69.2 (15.2)	71.3 (13.3)	70.2 (14.3)
Body mass index, mean (SD)^a^	25.2 (4.6)	25.0 (4.1)	25.1 (4.3)
Olanzapine equivalents, mean (SD), mg	9.3 (5.8)	9.1 (5.5)	9.2 (5.6)
Soft and hard drug use	31 (18.5)	35 (19.6)	66 (19.0)
Duration of psychosis, mean (SD), d	203 (477)	223 (598)	213 (529)

Abbreviation: EQ-5D-VAS, Visual Analog Scale of the EuroQo 5-Dimension Health-Related Quality of Life scale.

^a^ Calculated as weight in kilograms divided by height in meters squared.

**Figure.**
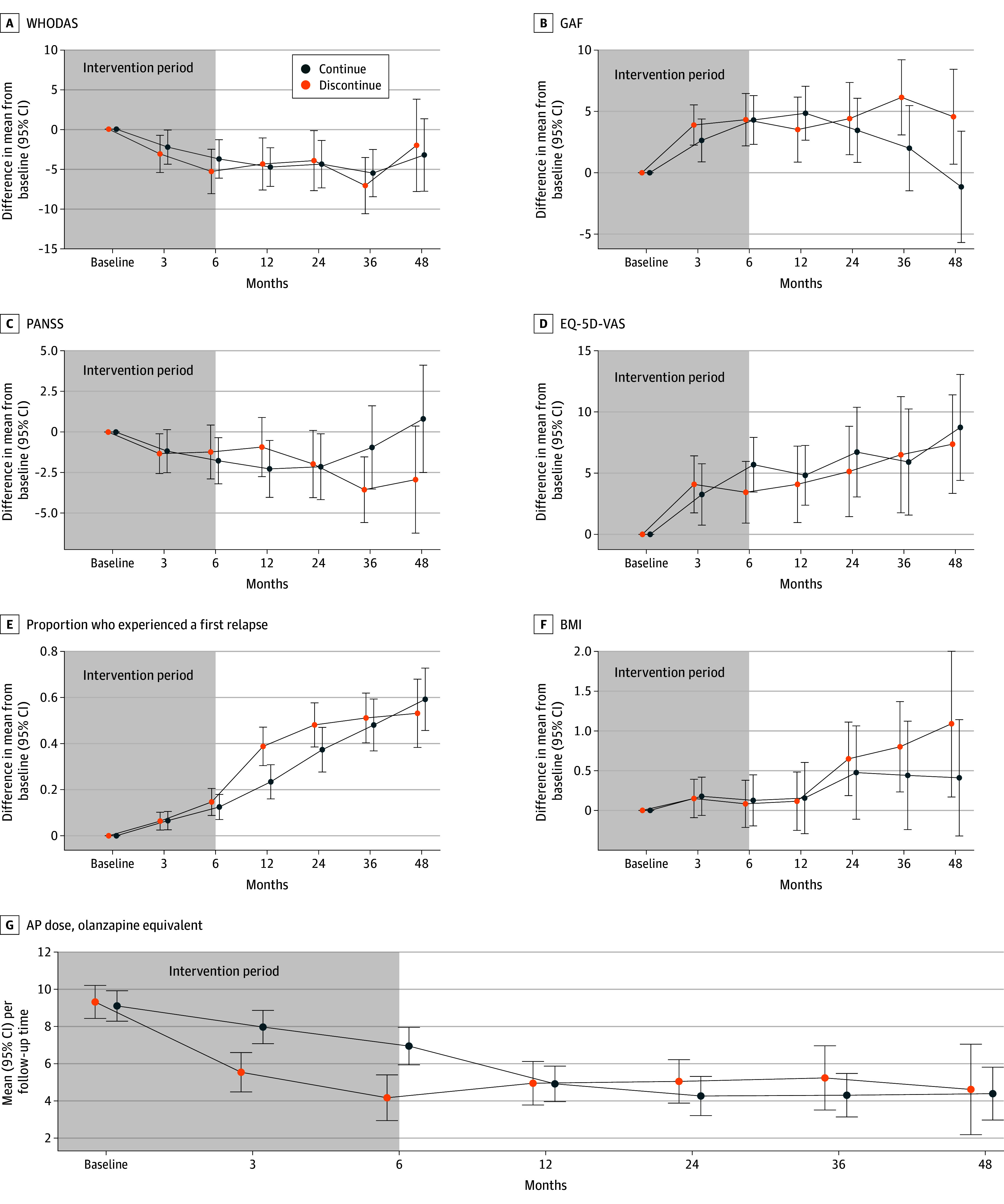
Mean Differences From Baseline Mean differences from baseline for the discontinuation or dose reduction group and the maintenance group for primary and secondary outcomes, plus olanzapine equivalences in the intention-to-treat analyses. AP indicates antipsychotic; BMI, body mass index; GAF, global assessment of functioning; PANSS, Positive and Negative Syndrome Scale; EQ-5D-VAS, Visual Analog Scale of the EuroQol 5-Dimension Health-Related Quality of Life scale; WHODAS-2, World Health Organization Disability Assessment Schedule 2.0.

**Table 2.** Outcomes of the Mixed Model for the Intention-to-Treat Analyses

Measure	Outcome, β (95% CI)					
	3 mo	6 mo	12 mo	24 mo	36 mo	48 mo
WHODAS	β = 1.20 (−1.65 to 4.06)	β = 0.15 (−2.79 to 3.10)	β = 1.37 (−1.69 to 4.43)	β = 1.15 (−2.22 to 4.53)	β = 0.41 (−3.29 to 4.12)	β = 0.05 (−4.47 to 4.57)
*P* value	.41	.92	.38	.50	.83	.98
PANSS	β = 0.83 (−1.14 to 2.80)	β = 1.07 (−0.97 to 3.10)	β = 1.86 (−0.25 to 3.97)	β = 0.51 (−1.81 to 2.83)	β = −1.71 (−4.26 to 0.84)	β = −3.02 (−6.17 to 0.13)
*P* value	.41	.30	.08	.67	.19	.06
GAF	β = 0.76 (−1.79 to 3.31)	β = −0.48 (−3.11 to 2.16)	β = −1.73 (−4.45 to 1.00)	β = 0.93 (−2.07 to 3.94)	β = 3.61 (0.28 to 6.95)	β = 6.13 (2.03 to 10.22)
*P* value	.56	.72	.22	.54	.03	.003
EQ-5D-VAS	β = 0.58 (−2.38 to 3.54)	β = −3.31 (−6.34 to −0.29)	β = −2.99 (−6.31 to 0.33)	β = −3.31 (−6.82 to 0.21)	β = −0.91 (−5.17 to 3.36)	β = −1.20 (−5.90 to 3.51)
*P* value	.70	.03	.08	.07	.68	.62
Body mass index	β = −0.02 (−0.48 to 0.44)	β = −0.14 (−0.61 to 0.33)	β = 0.09 (−0.39 to 0.58)	β = 0.33 (−0.21 to 0.87)	β = 0.51 (−0.08 to 1.11)	β = 0.28 (−0.41 to 0.96)
*P* value	.94	.57	.70	.23	.09	.43
Proportion relapse^a^	1 [Reference]	1.77 (0.64 to 5.07)	2.84 (1.08 to 7.66)	2.56 (0.98 to 6.83)	2.13 (0.82 to 5.71)	1.85 (0.71 to 4.97)
*P* value	NA	.28	.04	.06	.12	.21
Olanzapine equivalent	β = −2.42 (−3.49 to −1.35)	β = −2.77 (−3.83 to −1.7)	β = −0.82 (−1.89 to 0.25)	β = −0.19 (−1.26 to 0.87)	β = −0.24 (−1.31 to 0.83)	β = −0.28 (−1.35 to 0.79)
*P* value	<.001	<.001	.13	.72	.66	.60

Abbreviations: EQ-5D-VAS, Visual Analog Scale of the EuroQo 5-Dimension Health-Related Quality of Life scale; GAF, Global Assessment of Functioning; NA, not applicable; PANSS, Positive and Negative Syndrome scale; WHODAS, World Health Organization Disability scale.

^a^ Values for this category are presented as odds ratios with 95% CIs.

### Safety and Tolerability

Both groups had similar distributions in self-harm, violent behavior, police contacts, and neurological adverse effects (eAppendix 7 in Supplement 2). In the DRD group, 5 deaths, 32 hospitalizations, and 3 other SAEs occurred compared with 1 death, 42 hospitalizations, and 1 other SAE for the maintenance group. The DRD group had 3 confirmed deaths by suicide, while the maintenance group had 1 death by suicide.

### Per-Protocol Analyses

The DRD group had higher GAF scores at 6, 24, 36, and 48 months. The DRD group had lower PANSS scores at 6, 24, 36, and 48 months, with lower BMI at 12 and 24 months (eAppendix 8 in Supplement 2).

### Sex at Birth Subanalyses

Men in the DRD group had lower quality of life at 6 and 12 months and better GAF scores at 48 months. Women in the DRD group had better GAF scores at 36 and 48 months (eAppendix 9 in Supplement 2).

### Sensitivity Analysis Including Only Schizophrenia or Schizoaffective Diagnoses

The DRD group had more relapse at 12 and 24 months. The DRD group also had better GAF scores at 48 months, with higher BMI at 24 and 36 months (eAppendix 10 in Supplement 2).

## Discussion

This randomized clinical trial compared early DRD with maintenance treatment in 347 remitted patients with FEP. Patient-rated functioning (WHODAS-2) showed no difference between conditions. Quality of life was lower for those in the DRD group, with more relapse in the first year. The number of SAEs was similar, but 3 confirmed deaths by suicide occurred in the DRD group vs 1 in the maintenance condition.

At 36 months, GAF was better for the DRD group, an effect that became even larger at 48 months, which parallels Wunderink et al.^4^ A similar trend was observed for symptom severity.

The per-protocol analyses showed no disadvantages and additional benefits for DRD. For women, DRD had fewer disadvantages and more benefits, which could reflect relative overdosing of female patients.

Beneficial longer-term effects for the DRD condition cannot be attributed to direct effects of antipsychotic medication because doses were similar from 12 months onwards. Rather, a guided tapering attempt may strengthen therapeutic alliance and disease insight. Symptom return after guided DRD can help patients accept antipsychotic medication as a resource supporting mental stability, a crucial step in coping with psychotic vulnerability. Patients from a Danish tapering clinic reported more positive attitudes toward medication after discontinuation and reinstalling medication on relapse.^11^ The potential learning and empowering element of DRD needs to be weighed carefully against short-term risks. For a well-balanced decision, sex at birth could be relevant because women experienced fewer negative effects and more benefits of DRD.

### Limitations

This study has limitations. Patient-rated functioning (WHODAS-2) was affected by unrealistic scoring of participants experiencing challenges in recognizing social and communicative aspects of their condition.^12^ In the maintenance group, 40% tapered medication against protocol, which reflects clinical practice where a similar percentage of patients with FEP is nonadherent.^13^ Our findings cannot be extrapolated to multiepisode patients because their situation may be quite different. The trial by Liu et al^14^ randomized stable multiepisode patients to dose reduction (but not discontinuation) or maintenance treatment and found no difference in social functioning. The trial by Moncrieff et al^15^ also showed no benefit of DRD over maintenance in social functioning in multiepisode patients, with more relapse after DRD.

## Conclusions

In this randomized clinical trial, while our primary outcome showed no difference, we found lower quality of life and more relapse at 12 months and better researcher-rated functioning at 3- and 4-year follow-up for the DRD condition. Because doses were similar from 12 months onwards, we interpret these long-term benefits as reflecting an empowering and insightful experience, rather than direct effects of antipsychotic reduction.

## Notes

Supplement 1.
Trial Protocol and Statistical Analysis Plan

Supplement 2. **eAppendix 1.** Study Design and Randomization **eAppendix 2.** Definition of Cross-Overs and Tapering Procedure **eAppendix 3.** Calculation of Olanzapine Equivalents **eAppendix 4.** CONSORT Flow Diagram **eAppendix 5.** Baseline Medication Use **eAppendix 6.** Mean (SD) Scores on Outcome Measures per Condition and Timepoint **eAppendix 7.** Safety and Tolerability: Detailed Information on SAE, AE, Self-Harm, Violence, Police Contact and Neurological Side-Effects **eAppendix 8.** Per Protocol Analyses: Generalized Mixed Models **eAppendix 9.** Sex-at-Birth Subanalyses: Generalized Mixed Models **eAppendix 10.** Sensitivity Analysis Including Only Patients With Diagnosis of Schizophrenia and Schizo-Affective Disorder

Supplement 3.
Nonauthor Collaborators

Supplement 4.
Data Sharing Statement
