# Supplementary material for: Early Dose Reduction or Discontinuation vs Maintenance Antipsychotics After First Psychotic Episode Remission: A Randomized Clinical Trial
Source: JAMA Psychiatry. 2025 Oct 1;83(1):68–73. doi: 10.1001/jamapsychiatry.2025.2525 (PMC12489793; doi:10.1001/jamapsychiatry.2025.2525)
Supplement: Supplement 1. — Trial Protocol and Statistical Analysis Plan [file jamapsychiatry-e252525-s001.pdf]

HAMLETT

Handling Antipsychotic Medication: Long-term Evaluation of Targeted Treatment

A pragmatic single-blind randomized controlled trial of continuation versus discontinuation/ dose reduction of antipsychotic medication in patients remitted after a first episode of psychosis

Research Protocol

Dossier nr 80-84800-98-41015

Hoofdaanvrager: Prof. dr. I.E.C. Sommer

Universitair Medisch Centrum Utrecht

April, 2023

**Protocol ID**	ABR62202
**Short title**	**HAMLETT**
**EudraCT number**	**[2017-002406-12]**
**Version**	**1.11**
**Date**	**April, 2023**
**Coordinating investigator/ project leader**	**Dr. M.J.H. Begemann**<br>M.J.H.Begemann@umcutrecht.nl<br>University Medical Center Utrecht<br>Department of Psychiatry<br>Phone: +31 88 75 69599
**Principal investigator**	**Prof. Dr. I. E. C. Sommer**<br>i.e.c.sommer@umcg.nl<br>University Medical Center Groningen<br>Department of Psychiatry<br>Phone: +31 50- 3614106
**Sponsor (in Dutch: verrichter/opdrachtgever)**	University Medical Center Groningen<br>Department of Neuroscience<br>Phone: +31 50- 3614106
**Subsidising party**	**ZonMw**<br>The Netherlands Organisation for Health Research and Development<br>www.zonmw.nl
**Independent expert**	**Prof. Dr. R.C. Oude Voshaar**<br>r.c.oude.voshaar@umcg.nl<br>University Medical Center Groningen<br>Department of Psychiatry<br>Phone: +31 50- 3615719
**Laboratory sites**	**Mrs. A. Bos**<br>a.bos02@umcg.nlUniversity Medical Center Groningen<br>Laboratory for Clinical Trials<br>Phone: +31 50- 3613201<br><br>**Mrs. L. Delhaas**<br>L.Delhaas@umcutrecht.nl

	University Medical Center Utrecht<br>Laboratory for Clinical Chemistry and Haematology<br>Phone: +31 88 75 51557
**Pharmacy**	*No study medication will be dispensed.*
**Coordinator study monitoring**	**Mrs. D. Maily**<br>d.maily@umcg.nl<br>University Medical Center Groningen<br>Phone: +31 50- 3615598

PROTOCOL SIGNATURE SHEET

Name	Signature	Date
**Head of Department:**<br><br>**Prof. Dr. R. A. Schoevers**<br>**Head of Department of Psychiatry in the UMC Groningen, psychiatrist**		**March, 2023**
**Principal Investigator:**<br><br>**Prof. Dr. I.E.C. Sommer**<br>**Neuroimaging Centre UMC Groningen, psychiatrist**		**March, 2023**

TABLE OF CONTENTS

1. INTRODUCTION AND RATIONALE ... 12
   1.1 Problem definition ... 12
   1.2 Review of the literature ... 12
2. OBJECTIVES ... 14
   2.1 Objective 1: Trial-based Hypotheses Testing ... 14
   2.2 Objective 2: Ecological Momentary Assessment ... 14
   2.3 Objective 3: Health-Economic Evaluation and Prognostic Modelling ... 14
   2.4 Objective 4: Implementation ... 15
3. METHOD ... 16
4. STUDY POPULATION ... 17
   4.1 Population ... 17
   4.2 Inclusion criteria ... 17
   4.3 Exclusion criteria ... 17
   4.4 Sample size calculation ... **Error! Bookmark not defined.**
5. TREATMENT OF SUBJECTS ... 18
   5.1 Continuation condition ... 18
   5.2 Discontinuation/dose reduction condition ... 18
6. METHODS ... 19
   6.1 Study parameters/endpoints ... 19
   6.2 Randomization and blinding ...
   6.3 Study procedures ... 21
   6.4 Measurements ... 23
   6.5 Ecological Momentary Assessments ... 31
   6.6 BeHapp ... 33
   6.7 Raclopride PET ...
   6.8 MRI and OCT study ...
   6.9 Personal recovery and qualitative interviews ...
7. SAFETY REPORTING ... 37
   7.1 AEs, SAEs and SUSARs ... 37
   7.2 Annual safety report ... 37
   7.3 Follow-up of adverse events ... 38
   7.4 Data Safety Monitoring Board ... 38
8. STATISTICAL ANALYSIS ... 39
   8.1 Lost to follow-up and data-management ... 39
   8.2 Interim analyses ... 39
   8.3 Analyses of clinical outcome ... 40
   8.4 Health-economic evaluation ... 40
   8.5 Prognostic modelling ... 43
9. OPTIMISING IMPLEMENTATION DURING THE TRIAL ... 44
   9.1 Implementation and implementation research ... 44
   9.2 Tasks and responsibilities ... 44
   9.3 Measurements, measures and analyses implementation research ... 45

9.4 Deliverables implementation research ... 48
10. FEASIBILITY ... 49
11. ETHICAL CONSIDERATIONS ... 50
11.1 Regulation statement ... 50
11.2 Recruitment ... 50
11.3 Benefits and risks assessment, group relatedness ... 50
11.4 Compensation for injury ... 50
11.5 Incentives ... 51
12. ADMINISTRATIVE ASPECTS, MONITORING AND PUBLICATION ... 51
12.1 Handling and storage of data and documents ... 51
12.2 Monitoring and Quality Assurance ... 51
12.3 Amendments ... 52
12.4 Annual progress report ... 52
12.5 End of study report ... 52
12.6 Public disclosure and publication policy ... 52
13. REFERENCES ... 53

LIST OF ABBREVIATIONS AND RELEVANT DEFINITIONS

ABR	ABR form, General Assessment and Registration form, is the application form that is required for submission to the accredited Ethics Committee (In Dutch, ABR = Algemene Beoordeling en Registratie)
AE	Adverse Event
Aggression and self-harm	Questionnaire about aggression and self-harm
AGE	Advanced Glycation End products
AHA/NHLB	American Heart Association/National Heart, Lung and Blood Institute
BACS	Brief Assessment of Cognition in Schizophrenia
BARS	Barnes Akathisia Rating Scale
BeHapp	Smartphone Application
BIA	Budget Impact Analysis
BMI	Body Mass Index
BRS	Brief Resilience Scale
BSS	Bristol Stool Scale
CART	Classifications and Regression Tree Analysis
CASH	Comprehensive Assessment of Symptoms and History
CBS	Statistics Netherlands; in Dutch: Centraal Bureau voor de Statistiek (CBS)
CBT	Cognitive Behavioural Therapy
CEA	Cost-Effectiveness Analysis
CFIR	Consolidated Framework of Implementation Research
CHEERS	Consolidated Health Economic Reporting Standards
CONSORT	Consolidated Standards of Reporting Trials
CRP	C-Reactive Protein
CSFQ	Changes in Sexual Functioning Questionnaire
CTQ-SF	Childhood Trauma Questionnaire-Short Form
CUA	Cost-Utility Analysis
DDD	Daily Defined Dosage
dL	Deciliter
DNA	Desoxyribo Nucleic Acid
DSMB	Data Safety Monitoring Board
ECG	Electrocardiography

**eCRF**	**Electronic Case Report Form**
**EDTA**	**Ethylenediaminetetraacetic acid**
**HDL-C**	**High-Density Lipoprotein Cholesterol**
**EMA**	**Ecological Momentary Assessment**
**EQ-5D-5L**	**EuroQol**
**ERB**	**Ethical Review Board**
**EudraCT**	**European drug regulatory affairs Clinical Trials**
**EULAST**	**European Long-acting Antipsychotics in Schizophrenia Trial**
**FEP**	**First Episode Psychosis**
**FFQ**	**Food Frequency Questionnaire**
**GAF**	**Global Assessment of Functioning**
**GCP**	**Good Clinical Practice**
**GSRS**	**Gastro-intestinal Symptom Rating Scale**
**HAMLETT**	**Handling Antipsychotic Medication: Long-term Evaluation of Targeted Treatment**
**HDL-C**	**High Density Lipoprotein-C**
**HRV**	**Heart Rate Variability**
**IC**	**Informed Consent**
**ICER**	**Incremental Cost-Effectiveness Ratio**
**ICH-GCP**	**International Conference on Harmonisation-Good Clinical Practice**
**INBRA**	**Incremental Net-Benefit Regression Analysis**
**IPS**	**Individual Placement and Support**
**ITT**	**Intention To Treat**
**LOCF**	**Last-Observation Carried Forward**
**LTE**	**List of Threatening Experiences**
**METC**	**Medical research ethics committee (MREC); in Dutch: Medisch Ethische Toetsings Commissie (METC)**
**mg**	**Milligrams**
**MINI-Screener**	**Mini-International Neuropsychiatric Interview Screener**
**MSPSS**	**Multidimensional Scale of Perceived Social Support**
**NB**	**Net-Benefit**
**NBA**	**Net-Benefit Analysis**
**OPTiMiSE**	**Optimization of Treatment and Management of Schizophrenia in Europe**
**PANSS**	**Positive And Negative Symptom Scale**
**PAS**	**Premorbid Adjustment Scale**

PICOT	Population, Intervention, Comparator, Outcomes, Time horizon
PSS-10	Perceived Stress Scale
PsyMod	Psychological Modification
QALY	Quality Adjusted Life Year
RAS	Recovery Assessment Scale
RCT	Randomized Controlled Trial
(S)AE	(Serious) Adverse Event
SD	Standard Deviation
SES	Socioeconomic Status
SHRS	St. Hans Rating Scale
Sponsor	The sponsor is the party that commissions the organisation or performance of the research, for example a pharmaceutical company, academic hospital, scientific organisation or investigator. A party that provides funding for a study but does not commission it is not regarded as the sponsor, but referred to as a subsidising party.
SRA	Subjective Reaction on Antipsychotics
SST	Serum Separator Tube
STG	Superior Temporal Gyrus
Stroop	Interference Test
SUSAR	Suspected Unexpected Serious Adverse Reaction
SWN	Subjective Wellbeing on Neuroleptics Scale
TiC-P	Trimbos/iMTA Questionnaire on Costs associated with Psychiatric Illness
UMCG	University Medical Center Groningen
UMCU	University Medical Center Utrecht
V	Visit
WHO-DAS-II	World Health Organization’s Disability Asesseement Schedule
WiFi	Wireless Fidelity
WMO	Medical Research Involving Human Subjects Act (in Dutch: Wet Medisch-wetenschappelijk Onderzoek met Mensen)
WP	Work Package
WTP	Willingness To Pay

113

SUMMARY

Rationale: Antipsychotic medication is effective for symptomatic remission in schizophrenia-spectrum disorders. After remission, studies comparing antipsychotic continuation to discontinuation find lower relapse rates and lower symptom severity in the continuation group. Therefore, guidelines recommend treatment continuation with standard dose antipsychotic medication for at least one year. Recently, these guidelines have been questioned. Long-term outcome in terms of recovery includes social functioning and quality of life, in addition to relapse prevention and lower symptom severity. Recovery may not benefit from continuation treatment with standard dose antipsychotic medication. Until now, only one randomized controlled study with a long follow-up time has been conducted and showed that patients who discontinued or reduced their antipsychotic medication in the early phase after remission more often reached recovery (40.4%) than those who continued treatment (17.6%). In response to this finding, many clinicians now recommend discontinuation following remission of first episode psychosis, thereby diverging from current guidelines. Moreover, many patients express the wish to discontinue medication at an early stage. Furthermore, what factors influence long-term prognosis (within the crucial first 10 years) after a first psychotic episode and in which way are not sufficiently known.

Objective: At present, patients, their family as well as clinicians need to know whether continued standard dose antipsychotic medication is beneficial or harmful after remission of first episode psychosis. This question not only concerns the short-term (i.e. after 1 or 2 years), but also the more relevant long-term (after 3 and 4 years) outcome. Furthermore, the unique longitudinal data from a large group of patients from disease onset covering the whole critical period of ten years after onset provides key information about risk factors and outcome.

Study design: We propose a pragmatic single blind superiority trial & cohort study.

Study population: Participants (n=512) aged 16-60 years, who are in stable remission from a first episode of psychosis for 3-6 months and for whom psychosis was not associated with life threatening self-harm or violence. Recruitment will take place at 22 Dutch sites.

Intervention: Patients are randomized 1:1 to

1. *Treatment as usual:* continuation of antipsychotic medication (original dose allowing a 25% reduction or other antipsychotic drug in similar dose range) until at least 1 year after remission.
2. *Dose reduction/discontinuation:* gradual dose reduction according to a tapering schedule of approximately 12 weeks until medication is maximally reduced or completely stopped.

Eligible patients who have already discontinued their medication can participate without randomization. Measurements will be conducted at baseline, and at 3 and 6 months post baseline. Further follow-ups are scheduled over 10 consecutive years in cycles of one year.

Main study parameters/endpoints:

The primary outcome measure is social recovery (World Health Organization’s Disability Assessment Schedule (WHO-DAS–II)). Secondary outcome measures include: side effects of medication use, personal wellbeing, quality of life, symptom severity, physical health (e.g. body mass index, somatic comorbidity including metabolic syndrome), aggression and self-harm, cognitive functioning, movement disorders, number and duration of psychotic relapses, number and duration of psychiatric treatments, cigarette alcohol and drug abuse, language production, skin autofluorescence indicating oxidative stress, psychological stress and stress resilience. Ecological momentary assessments (EMA), are assessed via a smartphone diary app in a sub-sample of 88 patients, as measure of social functioning and well-being in daily life.

We will perform prognostic modelling analyses to determine patient and treatment characteristics that predict long-term social recovery (main outcome) and sustained gains in health-related quality of life. Furthermore, we will perform cost-effectiveness and cost-utility

analyses with social recovery and quality adjusted life years (QALYs) gained as the main outcomes, respectively. Finally, implementation of the study protocol will be assessed and improved in all participating centers during the trial.

Burden and risks associated with participation, benefit and group relatedness: The number of patient visits will be limited and mainly requires time investment for a few physical examinations, questionnaires and three cognitive testing sessions. Blood will be drawn at four occasions with negligible and known risks (e.g. irritation). The burden and risks are acceptable while the benefits are expected to be considerable.

1. INTRODUCTION AND RATIONALE

1.1 Problem definition

Continuation of antipsychotic medication after remission reduces chances for relapse more than twofold (i.e. 27% relapse rate with maintenance treatment against 64% relapse without medication) and reduces symptom severity (Leucht et al. 2012). Almost all guidelines therefore recommend continuation of treatment with a standard dose antipsychotic medication for at least one year (National Institute for Clinical Excellence guidelines 2014, [UK]; Early Psychosis Guidelines Writing Group, 2010 [Australia]; Multidisciplinaire Richtlijn Schizofrenie, 2012 [the Netherlands]). In clinical practice, patients often have a strong wish to stop medication earlier. This wish partly reflects side-effects such as weight gain, anhydonia, sleepiness and parkinsonism (Longden & Read, 2016).

Recently, the guidelines have been questioned because long-term outcome in terms of social recovery may not benefit from long-term use of antipsychotic medication (Longdon & Read, 2016). Only two studies investigated the influence of maintenance therapy on level of social functioning. At the short term, both studies found no significant difference between maintenance therapy and discontinuation or dose reduction (Gaebel et al., 2011, Wunderink et al., 2007). Only the study by Wunderink (2013) had a long follow-up time. It showed that after 7 years, patients in the discontinuation condition more often reached recovery, defined as meeting criteria for both symptomatic and functional remission (40.4%) in comparison to those on maintenance treatment (17.6%). In response to this finding, many clinicians apply discontinuation following remission of psychosis, thereby diverging from current guidelines (Thompson et al., 2016). At present, patients, their family as well as clinicians need to know whether maintenance treatment is beneficial after remission of psychosis or not with respect to ‘social recovery’ – thus going well beyond symptomatic remission.

Moreover, psychotic disorders have a largely unpredictable course over the first 10 years, the so-called ‘critical period’. After the critical period, outcome in the clinical and social domain is relatively stable. Some possible predictors of outcome, such as initial clinical care received, social environment and informal help, can be modified to improve outcome. At present, the influence of these latter parameters on outcome is largely unknown.

1.2 Review of the literature

Symptomatic remission and relapse with antipsychotic use

Using PubMed and Scopus databases, a systematic review was undertaken of articles published between January 1, 2000, and May 20, 2015, that reported randomized and non-randomized prospective clinical trials on the long-term effects of antipsychotics on measures of relapse or remission in patients with First Episode Psychosis (FEP) or early schizophrenia (Karson et al., Jan 2016, see literature review attached).

Thirteen studies met inclusion criteria, reporting on long-term outcomes in patients with FEP. Antipsychotic treatment in patients with FEP produced high rates of remission in the year following treatment initiation, and untreated FEP reduced the odds of later achieving remission. Continuation of antipsychotic medication was more effective than treatment discontinuation or intermittent/guided discontinuation in preventing relapse.

Another systematic review was aimed at evaluating the effect of dose reduction or discontinuation of antipsychotic medication on relapses (Alvarez-Jimenez et al., 2016). Seven randomized controlled trials were identified; some were also included in the Karson review article (2016). The studies compared either antipsychotic medication reduction or discontinuation with maintenance treatment in patients with FEP or early schizophrenia. Indeed, a higher rate of relapse was reported in the dose reduction or discontinuation groups. Importantly, only one study had a considerably long follow-up time of 7 years (Wunderink et al., 2013), whereas the other studies had a follow-up of 1 or 2 years. Wunderink and colleagues (2013) observed that relapse rates between the two groups (dose-reduction vs.

maintenance), although different at 2 years, were equal after 3 years, which is not in line with the other, shorter, studies. This outlier effect underlines the importance of more research with a long follow-up, as the long term effects of discontinuation or dose reduction on relapse rates are still to be determined.

Social recovery, well-being and cognition with antipsychotic use

Blockade of the dopamine D_2 receptors, the main mediator of efficacy of antipsychotic medication, can produce adverse subjective experiences or neuroleptic dysphoria (Haan et al., 2000, 2003, Bressan et al., 2000, Mizrahi et al., 2007), encompassing a variety of unpleasant subjective changes in arousal, mood, thinking and motivation (Voruganti & Awad, 2004). Some 50% of men and up to 70% of women report difficulty in concentrating or tiredness after start of treatment with antipsychotic medication (Barbui et al., 2005). Severity of these mental adverse effects depends on individual sensitivity and dose of antipsychotic medication. Individuals with lower baseline dopamine function are at increased risk for dysphoric responses during treatment with dopaminergic blocking drugs (Voruganti et al., 2001). With regard to dosage of antipsychotic medication, most mental adverse effects occur at D_2 receptor occupancy higher than 65-70% (Haan et al., 2003, Nimwegen et al., 2008).

In addition to dysphoria, dopamine blockade may remarkably reduce functioning by exerting negative effects on cognition. Dopamine plays an important role in learning and motivation, as it enables associative learning, especially of aversive stimuli (Insel et al., 2014). Blockade of this system reduces the cognitive capacity to learn new associations, which may hinder study or work (Arnsten et al., 2015). Blockade of the mesolimbic reward system reduces motivation and drive, which can be expected to reduce professional success (Ostlund et al., 2012).

Mental and cognitive adverse effects associated with higher doses may explain why functional recovery can improve when patients discontinue or reduce the dose of their antipsychotic medication. However, cognitive improvement after continuation of antipsychotic treatment in patients with FEP was found in seven out of seven studies, with a sustained effect of up to 2 years (Karson et al., 2016). Importantly, long term effects on cognition are still unknown, and none of the studies investigating cognition included a discontinuation arm.

Well-being was compared in patients randomized to continued medication or intermittent treatment and found no significant difference between the groups after one year follow-up (Gaebel et al., 2011). As earlier mentioned, only one study investigated the long term effects of continued medication versus discontinued/reduced antipsychotics with regard to functional recovery and observed more recovery in the discontinuation/dose reduction group after seven years follow-up (40.4% versus 17.6%) (Wunderink et al., 2013).

2. OBJECTIVES

The objectives are and hypotheses are specified per work package.

2.1 Objective 1: Trial-based Hypotheses Testing

To provide clear information as to whether patients who have remitted from a first psychotic episode should continue to use antipsychotic medication for at least one year or whether long term recovery is better when they gradually reduce their medication. This information provides the evidence for shared, well-informed medical decision-making for a large group of patients.

We will conduct a pragmatic single blind randomized superiority trial (RCT) testing the following hypotheses:

1. Social recovery (main outcome) is higher with an effect size of at least d=0.33 in the discontinuation/dose reduction group than in the continuation group.
2. Subjective wellbeing is higher in the discontinuation/dose reduction group.
3. Somatic health, including metabolic syndrome, is better in the discontinuation/dose reduction group.
4. Relapse and rehospitalisation rates are higher in the discontinuation/dose reduction group.

2.2 Objective 2: Ecological Momentary Assessment

To produce an e-health application to monitor daily fluctuations: A. to predict relapse at an early stage in individual patients, which has potential for increasing the safety of discontinuation strategies; and B. to use Ecological Momentary Assessment (EMA) as a magnifying glass to evaluate consequences of (dis)continuation of antipsychotic medication for social functioning and well-being in daily life.

We will track individual changes in mental states, sleep, activities and wellbeing using Ecological Momentary Assessment (EMA) during baseline, during either discontinuation or continuation and during the follow-up, testing the following expectation:

1. Early fluctuations in mood, sleep and well-being accurately predict outcome and success of discontinuation in individual patients.
2. EMA can be used as a magnifying glass to reveal differences in daily life social functioning and well-being associated with either discontinuation or continuation.
3. EMA can detect early clinical changes associated with medication reduction. Also, it is possible to detect early signs of impending psychotic relapse, for example in the form of ‘critical slowing down’ of ESM-level psychotic experiences. ‘Critical slowing down’ refers to the phenomenon that deviations from baseline take progressively longer to return to their original value, indicating that the system in question is increasingly fragile and close to a critical transition such as a relapse psychotic episode.

2.3 Objective 3: Health-Economic Evaluation and Prognostic Modelling

To shed light on the cost-effectiveness and cost-utility of discontinuation versus continuation with respect to social recovery (main outcome) and QALYs gained, with the aim to inform decision-makers and financiers in health care. Prognostic modelling will offer evidence-based guidance to the appropriate selection of candidates for discontinuation, thus addressing the question who benefits most of discontinuation, with the aim to enhance precision psychiatry.

We will conduct a cost-utility analysis (CUA) and cost-effectiveness analysis (CEA) alongside the trial with quality adjusted life years (QALYs) gains and social recovery as the main outcomes, respectively and conduct a series of prognostic modelling analyses to identify patient and treatment characteristics that may act as early predictors of (1) successful personal and social recovery (2) successful discontinuation in terms of relapses, and (3)

greater net-benefits (QALY gains valued in Euro minus health care costs). CUA and CEA will be evaluating the following expectations:

1. Superior cost-utility (with incremental cost per QALY gained) in the discontinuation/dose reduction group.
2. Better cost-effectiveness (with incremental costs per recovery) in the discontinuation/dose reduction group.

Prognostic modelling is driven by the following expectations:

1. Patients with longer duration of untreated psychosis, comorbid drug abuse, male gender, lower education and earlier onset of psychosis will have poorer prognosis.
2. Discontinuation/ dose reduction will be more successful in patients who have used lower dose of medication, or have used medication with relatively low D_2 receptor affinity (clozapine, quetiapine and olanzapine).
3. Social functioning will be superior in patients who participated in psychosocial interventions such as cognitive behavioural treatment (CBT) and individual placement and support (IPS).
4. Relapse rates after discontinuation will be lower in patients who received CBT and IPS.

2.4 Objective 4: Implementation

To optimise implementation of the study during the trial and to ensure that best practice is shared among other centres, with an eye to improve sustainability beyond the trial period and for scaling up.

We will optimise implementation of the study results in the Netherlands and on an international level during and after the trial. Future implementation will be feasible and successful for the following reasons:

1. The study will inform treatment guidelines about the best treatment option (i.e. discontinuation/dose reduction or continuation).
2. The study’s dose reduction/discontinuation protocols will be made available for clinical guideline development and are helpful for clinical guidance.
3. Implementation in the Netherlands will be rapid using the online version of the guidelines, which is written by the WP leaders of this study and updated frequently.
4. The many centres, covering almost the complete catchment area of the Netherlands, that are actively engaged in the trial are able to implement findings very fast during the trial and act as teaching centers for less specialized settings.
5. International implementation follows upon publication of the results in international journals and presentations on international conferences.

In this study, each objective will be addressed in dedicated Work Packages. Hence, we have seven work packages:

Work Package 1: Trial-based Hypotheses Testing
Work Package 2: Ecological Momentary Assessment
Work Package 3: Health-Economic Evaluation and Prognostic Modelling
Work Package 4: Blood markers
Work Package 5: Personal recovery
Work Package 6: Gene-environment interactions
Work Package 7: Implementation

3. METHOD

The study is designed as a health-economic evaluation alongside a single blind pragmatic randomized superiority trial in two parallel groups with a follow-up time of 10 years.

We opted for this design as this study aims to provide clear guidance for doctors and patients on short and long-term benefits and disadvantages of continuation and discontinuation/dose reduction of antipsychotic treatment. The population and their treatment should therefore resemble the general clinical situation as much as possible to increase ecological validity and also to pave the way to future implementation. In the present clinical situation, comorbidity, drug and alcohol abuse and low IQ occur in approximately half of patients. Patients use all types of antipsychotic medication and dosages vary largely (depending on both treatment centre and patient characteristics). Finally, it frequently occurs that medication is switched, dosages are changed, other medication is added, or medication is stopped, and as a result only few patients use stable dose and type over consecutive years.

The naturalistic set-up of the study has several consequences:

1. Exclusion criteria are kept as few as possible. Only when the safety of the participants is at risk, exclusion will follow. Patients with comorbidity, drug- and alcohol abuse or low IQ will be able to participate, so that the sample will reflect the general population of patients with psychosis and the study's outcomes will be generalizable.
2. Patients can start the trial using any type of frequently prescribed antipsychotic drug at any dose (within safety ranges). Discontinuation schedules are prepared for haloperidol, risperidone, olanzapine, quetiapine, clozapine and aripiprazol and can be implemented in a flexible way. This makes it logistically impossible to use a double-blind design.
3. Patients will remain in the study and be followed-up even when they re-start antipsychotic medication (in the discontinuation arm) or significantly lower, switch or even stop medication (in the continuation arm). Cumulative dose of antipsychotic drugs will be calculated at each follow-up to compare groups.

This study will be single-blind: only the assessor who performs the visits and conducts the interviews is blind for treatment arm of the patient. Medication is prescribed (open label) by the treating clinician, a tapering off schedule has been prepared for all common antipsychotic medications (further explained in K6 H: example tapering schedule, dd February 2019) The single-blindness has the disadvantage that both clinicians and patients may change their behaviour as a consequence of knowing their treatment arm. For example, clinicians may be inclined to pay more attention and plan more frequent appointments for patients who discontinue. Patients may be more careful in terms of avoiding extra stress and illicit drugs in the discontinuation arm. On the other hand, fear for relapse may increase stress in these patients. These potential confounding factors may obscure the pharmacological effects of discontinuation, which reduces scientific quality of the study. At the same time, these confounding factors also play an important role in daily clinical practice and therefore increase the real-life clinical usefulness of the study results. Total time of treatment sessions is noted and taken into account when comparing the treatment arms.

4. STUDY POPULATION

4.1 Population

This study will include 512 patients with a first episode of schizophrenia, schizoaffective disorder, schizophreniform disorder or those classified as Other Specified Schizophrenia Spectrum and Other Psychotic Disorder.

4.2 Inclusion criteria

In order to be eligible to participate in this study, a subject must meet all of the following criteria:

Treatment with antipsychotic medication has resulted in symptomatic remission of a first psychotic episode during 3-6 months. Remission is defined as sustained improvement of psychotic symptoms to the level that any remaining psychotic symptoms (such as hallucinatory experiences, unusual thought content, conceptual disorganisation) are mild, which means (consistent with international remission criteria) that they do not interfere with behaviour and daily functioning. Exacerbation of positive symptoms is allowed only when judged to be related to single time substance use and not longer than a few days. First day of remission will be estimated by the treating clinician (sometimes retrospectively).

1. The participant has had a first episode of psychosis and uses antipsychotic medication. Eligible patients who have already discontinued their medication can participate without randomization.
2. Psychotic symptoms are in remission for 3-6 months.
3. Age 16-60 years.
4. The participant understands the study and is able to provide written informed consent.
5. HAMLETT is the only medical-scientific medication study in which the patient participates, during the first four years.
6. Sufficient command of the Dutch language.

4.3 Exclusion criteria

1. Dangerous or harmful behaviour (i.e. behaviour with a risk of severe physical injury, or actual physical injury inflicted, to self or others) occurred during the psychosis.
2. Coercive treatment (based on a judicial ruling).

All patients fulfilling the inclusion and exclusion criteria as described above may participate in the study.

5. TREATMENT OF SUBJECTS

5.1 Continuation condition

Patients randomized to the continuation condition are treated according to general Dutch guidelines, which recommend at least 1 year continuation after remission. During this year, medication will be kept within the same range, allowing a 25% dose reduction. After that first year, a shared decision is made for further continuation or gradual discontinuation based on the patients' motivation, the clinical situation (presence of symptoms and side effects) and the family's opinion. When discontinuation is considered, the tapering schedule (K6 H: example tapering schedule, dd February 2019) can be used. Patients and their treatment team may diverge from this regimen for several reasons (i.e. intolerable side effects, insufficient efficacy, strong wish of the patient) in any case the patients will remain in the study.

5.2 Discontinuation/dose reduction condition

This is a pragmatic study in which patients are included who use any frequently prescribed type of antipsychotic medication at any allowed dose range. Discontinuation schedules and tapering strips are prepared for most antipsychotic drugs available in the Netherlands (haloperidol, risperidone, quetiapine, olanzapine, clozapine and aripiprazole) and can be used flexibly. Approximately 90% of first episode patients use one of these agents. Medication is prescribed by the treating physician throughout the study. Patients using clozapine can also be included as long as inclusion criteria are met and exclusion criteria are not met. Patients with depot medication can also participate. Eligible patients who have already discontinued their medication before inclusion, can participate but will not be randomized.

The discontinuation/ dose reduction will start after 3-6 months of sustained remission from psychosis. We chose to start discontinuation not earlier than 3 months to be certain that patients have reached stable remission. The excel sheet (see further) provides guidelines for tapering off all available antipsychotic medication. The schedule provides a slow tapering schedule with a mean duration of 3 months until complete discontinuation. Patients who use higher dose at baseline will need longer until total stop. These guidelines should be followed flexibly, with the possibility to deviate from them when deemed appropriate by the patient, family or physician. Patients and their treatment team may opt to halt discontinuation at a certain dose when symptoms tend to re-appear, in which case they will remain in the study even though further discontinuation is not deemed possible. In case early warning signs occur (see safety procedures), further tapering of antipsychotic medication will be halted until early warning signs disappear. Professional caregiver will contact the patient regularly. Stress reduction will be advised. When early warning signs disappear, tapering of antipsychotic medication can be resumed. When early warning signs become more severe, the dosage of antipsychotic medication will be increased to one level higher [in other words: back to the former step] of the tapering scheme. When psychotic symptoms occur, treatment with antipsychotic medication will be restarted in the dose that patients used when the first symptomatic remission occurred.

A discontinuation schedule has been prepared for all common antipsychotic medications (further explained in K6 H: example tapering schedule, dd February 2019) and can be implemented in a flexible way, both for oral and depot antipsychotic medication. Treating physicians prescribe the tapering schedule that fits the patient's type and dose of baseline medication Duration of discontinuation thus depends on starting dose, with a maximum of 6 months for patients that use the maximal dose of an antipsychotic agent. In general, patient with a first psychotic episode use less than the mean dose, which means that their discontinuation phase is shorter than 12 weeks. The choice on how to taper off medication is made by the treating physician and the patient, not by the study team. In a letter for the treating physician, the study team provides information on the several possibilities together with instructions about their patient participating in the HAMLETT study.

6. METHODS

6.1 Study parameters/endpoints

6.1.1 Primary outcome measure

- WHO-DAS-II ability scale (Chopra et al., 2008).

As the patients who use antipsychotic medication are the main stakeholders, the patient organization Anoiksis performed a survey to investigate the outcome measure deemed most important in the decision to either continue or discontinue medication. Patients who have experienced one or more psychotic episodes and who are associated with the patient organization were invited by email to provide us with their preferences. Possible outcome measures were defined narrowly as: psychotic relapse, re-hospitalization, experiencing psychotic symptoms, having a paid job, having satisfactory social relationships, somatic health, obesity, parkinsonian side-effects, depressed mood or anxiety. Multiple outcomes could be affirmed as important. Forty-six invited members completed the survey and the two most frequently preferred outcome measures were: having satisfactory relationships and participating in society. Quantification of these preferences is best provided by using the WHO-DAS-II ability scale (Chopra et al., 2008). The WHO-DAS–II assesses disability in individuals irrespective of diagnosis across multiple life domains. The nature of disability is rated directly from individuals' responses. We will use the interview version consisting of 36 items, which covers all the relevant domains of social recovery: Cognition – understanding & communicating, Mobility – moving & getting around, Self-care – hygiene, dressing, eating & staying alone, Getting along – interacting with other people, Life activities – domestic responsibilities, leisure, work & school and Participation – joining in community activities.

6.1.2 Secondary outcome measures

- Side effects and subjective reactions to the use of mental health medications (Subjective Wellbeing on Neuroleptics Scale, SWN; Naber, 1995; Subjective Reaction on Antipsychotics, SRA; Wolters et al., 2003; Changes in Sexual Functioning Questionnaire, CSFQ (male and female version); Clayton et al., 1997).
- Physical health (body mass index and somatic comorbidity including metabolic syndrome)
- Quality of life assessed with the EuroQoL (EQ-5D-5L; Sonntag et al., 2015)
- The economic costs of health care uptake and productivity losses with the TiC-P (Hakkaart van Roijen, et al., 2002)
- Symptom severity as assessed with the Positive and Negative Symptom Scale (PANSS; Kay et al., 1987)
- The Premorbid Adjustment Scale (PAS; Cannon-Spoort et al., 1982) is a rating scale about five domains of functioning: sociability, peer relationships, scholastic performance, adaptation to school and social-sexual aspects. It covers two life periods: up to 12 and 12 to 16.
- Recovery Assessment Scale (RAS; Giffort et al., 1995)
- Multidimensional Scale of Perceived Social Support (MSPSS; Zimet et al., 1988)
- Aggression and self-harming behaviour (developed at the department of Psychiatry, UMC Utrecht).
- Personal recovery as measured by the Questionnaire Personal Recovery.
- Personal experience with discontinuation as measured by the Qualitative interview Personal Experiences of discontinuation antipsychotics medication.
- Self-esteem with (SERS-S; Lecomte et al., 2006)
- Internalised stigma of mental illness (ISMI; Ritsher et al., 2003)
- Psychosis attachment measure (PAM; Berry et al., 2008)
- Cognitive functioning as assessed with the Brief Assessment of Cognition in Schizophrenia (BACS; Keefe et al., 2004) and the Stroop task (Stroop, 1935).

- Movement disorders as assessed with the St. Hans rating scale (Gerlach et al., 1993) and Barnes Akathisia Rating Scale (BARS; Barnes, 1989)
- Clinical variables, including number and duration of psychotic relapses; number and duration of psychiatric admissions; total time spent with the treatment team
- Cigarette, alcohol and drug abuse (as assessed with the alcohol and drug section of the Comprehensive Assessment of Schizophrenia: CASH; Andreasen e.a., 1992 and the WHO assist (Humeniuk et al., 2010).
- MINI-Screener (developed at the department of Psychiatry, UMC Groningen).
- Language production. Antipsychotic medication interacts with receptors in language related areas in the brain such as Broca's area, the insula, the precentral gyrus, and the superior temporal gyrus (STG) (Buchsbaum, Hickok, & Humpries, 2001; Wildgruber, Ackermann, Klose, Kardatzki, & Grodd, 1996). Therefore, antipsychotics are likely to influence language production in schizophrenia patients. Previous studies have shown that dopamine receptor blocking drugs decrease the speed of language production (Sinha, Vandana, Lewis, Jayaram, & Enderby, 2015). By analysing speech at different time points during the study, we aim to analyse the effect of antipsychotic medication on language production in greater detail.
- Ecological momentary assessments (EMA; Bos, 2015), measuring momentary positive/negative affect, paranoia, hallucinations, sleep, social company and activities are assessed via a smartphone diary app. These assessments will be performed in a sub-sample of patients in both arms, for this purpose six centers will be asked to include the EMA package. At baseline, 6 months, 1, 2, 3 and 4 years follow-up, EMA will be completed daily for eight consecutive days at eight semi-random moments during the day, with emphasis on quantity and quality of social interactions. Also, during the discontinuation or continuation phase, a selected sub-sample of 30 patients will complete an intensive series of EMA during 16 weeks, in order to analyse within individuals to which degree early changes in the dynamic system of mental states predict future clinical change. For a detailed description, see section 6.5.
- BeHapp, measuring several aspects of daily life concerning the behaviour of humans are assessed via a smartphone application (as described in section 6.6).
- Data in the domains of work & income, health service and social support uptake, and living history (data collect from the Centraal Bureau voor de Statistiek (CBS)).
- State of the microbiome and immune system: for a subset of patients (n=40), a maximum of 4 stool samples will be collected at several timepoints. We will assess the state of their microbiome and immune system at these different intervals: preferably before discontinuing antipsychotic medication and after discontinuing antipsychotic medication. In these assessments, we will include three short self-rated questionnaires: visual assessment of the stool itself with the Bristol Stool Scale (BSS; Lewis & Heaton, 1997), gastro-intestinal complaints using the Gastro-intestinal Symptom Rating Scale (GSRS; Svendlund et al., 1988), a general overview of dietary habits using the Food Frequency Questionnaire (FFQ; Sluik et al., 2016) and a single question regarding mode of delivery (caesarean section or vaginal birth).
- We would like to ask a small number of patients (n=40) to participate in a qualitative interview with an experience worker and a researcher to investigate existential factors (i.e. mindset, personal philosophy, religion, spirituality) that may play a role in recovery. These interviews will be held four times over the course of 8 years and can be performed at the patient's home or in the hospital and will last some 2 hours.
- List of Threatening Experiences (LTE): measuring life events with considerable long-term contextual threat (Brugha et al., (1985).
- Skin autofluorescence: AGE (Advanced Glycation End products) levels in the skin measured with skin autofluorescence are a validated proxy for oxidative stress and accelerated aging, increased AGE levels have been observed in patients with recent-onset psychosis (Hagen et al., 2017).
- Psychological stress and stress resilience: Psychological stress experience over the past month will be measured with the 10-point version of the Perceived Stress Scale (PSS-10; Cohen et al., 1983). Stress resilience will be assessed with the Brief Resilience Questionnaire (BRS; Smith et al., 2008). Finally, heart rate variability (HRV) is measured using electrocardiography (ECG). HRV is a non-invasive

physiological measure of parasympathetic nervous system activation, which is a validated proxy for stress resilience (Perna et al., 2020). Participants will be asked to wear a chest belt with an ECG sensor, and resting state HRV will be measured for ~10 minutes while the participant relaxes in a sitting position with their eyes closed. PSS-10 and BRS are short questionnaires which are added to each visit. The HRV assessments will only be performed for a subset of patients, in the visits where blood is drawn and/or stool samples are collected. This is due to the aim to relate levels of stress and stress resilience to gut microbiota and immune markers.

6.2 Randomization and blinding

6.2.1 Randomization

Randomization of study participants will be performed by the Research Data Support of the University Medical Center Groningen. A web-based application will be used, randomization will be stratified according to the collaborating centres Randomization outcome is communicated directly to the treating physician together with a suggestion for discontinuation/dose reduction schedule if patients are randomized to this group. The assessor who performs the visits is not informed about the results of randomization. Eligible patients who have already discontinued their medication, can participate without randomization.

6.2.2 Blinding

The assessor will be kept blind to treatment allocation of the patient. The assessor is not involved in the treatment of the participants and asks the patient on each visit not to disclose his/her treatment allocation. When blinding is broken, for example because a patient talks about his medication use or discontinuation, the assessor is replaced by another, who is still blind. The assessor team will consist of one post-doc experienced with clinical trial management (for supervision, training and logistics) and three junior assessors, so that ample replacement is possible.

6.3 Study procedures

6.3.1 Informed consent procedure

To avoid selection bias, all patients with first episode psychosis upon referral to their treatment team(s) (using initials and date of birth) will be noted in a list by the dedicated includer, as described below (see 6.3.3). Patients who are thought to meet the inclusion criteria are approached, their treating physician will be notified.

In each center, a local dedicated includer appointed by the HAMLETT study (for a detailed description see 6.3.3 and noted in the delegation log) provides information to the patient and when possible his family (the information letter including the informed consent form, and the short information brochure). The patient and his family are given 2 weeks (or longer if needed) to consider participation. A second appointment is made (vis a vis) in which extra questions can be answered and in which oral and written information is provided. The patient is encouraged to council his significant others and the independent physician for advice. Additional questions are answered and if the patient opts to participate, the informed consent is signed by both the patient and the dedicated includer or a member of the central study team. The treating physician decides if the patient is able to make a well-informed decision. For participants under the age of 18, both parents will be asked to write an assent in addition to the patient’s consent. Before signing of the informed consent form, no other study procedures will be executed, the dedicated includer arranges a first visit with the central study team.

6.3.2 Study phases

The study is divided into two phases:

Phase 1: the experimental phase, which lasts 6 months. In this phase, patients in the discontinue group will follow a discontinuation schedule, which takes approximately 3 months. All patients will have 4 visits: the screening visit, the baseline measurement, and visits after 3 and after 6 months.

Phase 2: the follow-up phase, which lasts 9.5 years. During the follow-up phase participants will be assessed annually, during ten visits. In this phase some of the patients from the continuation group may discontinue their medication if they have reached at least one year maintenance treatment. The long follow-up time is deemed necessary and relevant to obtain information on personal and social functioning, including cognition (understanding and communication), mobility (moving and getting around), self-care (hygiene, dressing, eating, staying alone), social functioning (interacting with other people), life activities (domestic responsibilities, leisure, work and school), participation (joining community activities) – as per the WHO-DAS-II.

FEP is usually diagnosed during adolescence, with a largely unpredictable course over the first 10 years, the so-called ‘critical period’. Social recovery varies between adequate functioning with friends, work, a partner and a family - to total social isolation. Finally, even when clinical outcome is poor, existential factors affect life fulfillment, which varies largely from valuable engagement to the experience of emptiness and uselessness. While outcome after the critical period can be good, poor outcome causes low quality of life for patients and their families, risk for dangerous situations (aggressive or suicidal acts) and high costs for health services and loss of productivity. After the critical period, outcome in the clinical and social domain is relatively stable, while even for patients with poor outcome, improvement in the existential domain can still be made. Longitudinal follow-up is necessary to provide a comprehensive impression of personal and social functioning well into adulthood, as well as to collect factors likely to predict course and outcome of psychosis (i.e. symptomatic remission, relapse, persistent non-remission, and disability level in personal and social functioning) over the critical 10-year period. A previous study (Wunderink et al., 2013) showed significant benefit of discontinuation only on the long-term, so this follow-up period is deemed essential.

Prolongation of the follow-up. extend the follow-up period with six years. During this period, patients will be contacted once a year for a visit. These visits are similar to the visits in year 2, 3 and 4 and consist of an interview lasting either 90 or 140 minutes tapping into general functioning, symptom severity, cognitive functioning, speech and use of medical care.

6.3.3 Personnel

<u>The central assessment team</u>

A central assessment team consisting of an experienced post-doc and three junior assessors perform all visits during phase 1 and phase 2. Logistics and timing of visits is also coordinated by the assessment team, as is training, inter-rated reliability, regular check of adhesion to protocol. The assessor is to remain blind to treatment allocation. We choose to use a central inclusion team for three reasons:

1. to decrease heterogeneity of multiple rater scores
2. to guarantee optimal training and inter-rater reliability of the assessors
3. to facilitate the single blindness, which is easier if assessors are not stationed at the inclusion sites. These assessors can conduct the informed consent procedure as well as all protocol procedures for recruited patients. The use of a central inclusion team with few well trained assessors largely reduces variability.

<u>Training of assessors</u>

An investigators' meeting will be arranged for the introduction of the study protocol and corresponding training in the use of these scales. Proper conduct of the assessment of the primary outcome measure with the WHO-DAS-II ability scale, and the secondary outcome measures including the PANSS interview, the assessment of movement disorders and the cognitive test battery, will be taught by experts using instructional videos and checked via the assessment of a test video. In addition, the team of assessors have twice per year meetings in which inter-rater reliability is assessed, new assessors are trained, intervision is given and protocol adherence is checked. The postdoc supervises the visit team during these meetings, necessary protocol amendments and changes to the Standard Operating Procedures.

<u>Dedicated includers</u>

Participating centres will employ a *dedicated includer*, which can be a nurse, psychologist or doctor working at the centre who is well involved in the treatment teams. These *includers* have a (part-time) function to facilitate inclusion and help doctors remind to invite patients who fulfil inclusion criteria. The *dedicated includers* will note all patients with first episode psychosis upon referral to their treatment team(s) (using initials and date of birth) in a list and follow up regularly to see which patient fulfils the inclusion criteria and willing to participate. The dedicated includers can also obtain informed consent.

Moreover, dedicated includers will also assess antipsychotic treatment during the trial when needed, regarding type, dosage, period of use and any switch of antipsychotic medication that will be documented during study visits. Medication use will be listed in a table, evaluating type of medication, reason for use, dosage (average dose per period), start and stop date, reason for stopping/switching of medication. Total history of psychopharmaca will be recorded, other co-medication will only be noted for the past two weeks. The dedicated includer will also be the coach to promote implementation of preliminary results during the trial. For that purpose, the coordinator and central study team will work together with these dedicated includers to start first and second implementation rounds.

6.4 Measurements

Study examinations scheduled in the course of the trial are listed in Table 1 below. Patients will travel to the study centers for each visit. If preferred by the patient and if study procedures allow it, an experienced researcher can visit the patient at home.

Table 1: Patient visits and examinations specified per visit

		Month	Informed consent, in-/exclusion criteria, diagnosis (CASH and MINI-Screener), demographic and clinical variables (CASH)	Medication use, adverse events, subjective reaction (SWN, SRA, CSFQ), physical health	WHO-DAS–II	EQ-5D-5L, TiC-P, PANSS, GAF, PAS, RAS, MSPSS, aggression and self-harm, language production, List of threatening experiences, PSS-10, BRS	Blood samples	Cognitive assessment (BACS, Stroop)	Movement disorders (SHRS, BARS)	Clinical variables (i,e,relapses/ hospitalisation, time spent with treatment team), Drug/alcohol use (CASH, WHO-assist)	EMA *(subgroup of patients)*	BeHapp	Childhood trauma (CTQ-SF), PAS, Hearing impairment, Questionnaire Personal Recovery,Childhood Bullying Questionnaire	Prognostic variables: SERS-S, ISMI, PAM	Skin autofluorescence	Qualitative interview Personal Experiences of discontinuation	Qualitative interview to investigate existential factors recovery.	Stool samples**, HRV measurement***
**PHASE I**	V1 Screening	-3 to 0	X															
	V2 Baseline	0		X	X	X	X	X	X	X	X	X		X	X**			X
	V3 Midterm	3		X	X	X				X			X					
	V4 Close-out	6		X	X	X	X	X	X	X	X			X		X		X
**PHASE II**	V5 Follow-up 1	12		X	X	X			X	X	X	X						
	V6 Follow-up 2	24		X	X	X	X		X	X	X						X	X
	V7 Follow-up 3	36		X	X	X			X	X	X	X						
	V8 Follow-up 4	48		X	X	X	X	X	X	X	X				X		X	X
	V9 Follow-up 5	60		X	X	X			X	X								
	V10 Follow-up 6	72		X	X	X			X	X								
	V11 Follow-up 7	84		X	X	X		X	X	X					X		X	
	V12 Follow-up 8	96		X	X	X			X	X								
	V13 Follow-up 9	108		X	X	X			X	X								
	V14 Follow-up 10	120		X	X	X		X	X	X					X		X	
Early termination visit*				X	X	X	X	X	X	X	X							

* Will be performed if a patient prematurely discontinues the study.
** Will be performed at a later visit (V3-V8) in subjects already included in the study.
*** Will also be administered during a different visit (V3-V14) in subjects for which a blood sample will be collected.

CASH = Comprehensive Assessment of Symptoms and History; MINI-Screener = Mini-International Neuropsychiatric Interview Screener; SWN = Subjective Wellbeing on Neuroleptics Scale; SRA = Subjective Reaction on Antipsychotics; WHO-DAS–II = World Health Organization's Disability Assessment Schedule; EQ-5D-5L = EuroQoL; PANSS = Positive And Negative Symptom Scale; GAF = Global Asssessment of Functioning; PAS = Premorbid Adjustment Scale; RAS = Recovery Assessment Scale; MSPSS = Multidimensional Scale of Perceived Social Support; Aggression and self-harm = Questionnaire about aggression and self-harm; BACS = Brief Assessment of Cognition in Schizophrenia; Stroop = interference test; SHRS = St. Hans Rating Scale, BARS = Barnes Akathisia Rating Scale; BMI = Body Mass Index; EMA = Ecological Momentary Assessments; BeHapp = smartphone application; CTQ-SF= Childhood Trauma Questionnaire-Short Form. SERS-S = Self-esteem Rating Scale Short form, ISMI = Internalized Stigma of

Mental Illness, PAM = Psychosis Attachment Measure, CSFQ = Changes in Sexual Functioning Questionnaire.

Visits phase I:

- V1 Screening: Patients will be screened for eligibility to the study, after informed consent is completed. Diagnosis will be checked using the Comprehensive Assessment of Symptoms and History (Andreasen et al., 1987) and using the MINI Screener (developed at the department of Psychiatry, UMC Groningen) Several demographical and clinical variables will be assessed, including date of birth, sex, educational level, zip code, living alone/together, profession, prior psychiatric disorders, date of diagnosis (CASH), premorbid functioning (PAS) and duration of untreated psychosis (retrospectively, as defined by fulfilling a score of ≥ 4 on one of the positive items of the PANSS during at least one whole day, until start of antipsychotic medication [adapted from Marshall et al., 2005]). Medication use (including antipsychotic use, hormonal contraception and other medication) will be evaluated, in addition to side effects and subjective reactions to these medications (SRA, SWN, CSFQ). Physical health will be checked in a standard physical examination (height, weight, waist circumference, Body Mass Index, blood pressure, pulse, EPS), in addition to medical history.
- V2 Baseline examination: At baseline, the patient will be randomized, unless the patient has already discontinued their medication. When necessary, clinical data (duration of untreated psychosis, duration and type of treatment, dose and type of previous medication used) are (re)assessed for prognostic modelling. In addition, prognostic modelling requires data collection on (early changes in) self-esteem (SERS-S), self-stigma (ISMI), and attachment (PAM). For the health-economic evaluation the EQ-5D-5L for assessing health-related quality of life and the TiC-P will be used for assessing the costs of health care uptake and productivity losses at baseline. At baseline, blood and stool samples will be taken and substance abuse will be measured using the appropriate section of the CASH. The use of concomitant medication and medical conditions/adverse events will be recorded. In addition, the standard measurements (rating scales, cognition, movement disorders and additional measures) will be used for the first time, including primary and secondary outcomes (see below for details of these scales).
- V3 (3 months post-baseline) + V4 (6 months post-baseline): The visits of phase I are scheduled 3 and 6 months after baseline. Some flexibility is allowed, as visits can be performed within a range of +/- 3 weeks. Visits will consist of all rating scales, blood and stool samples (V4) and a personal interview and questionnaires (V4): Childhood trauma (CTQ-SF), premorbid functioning (PAS), Hearing impairment, Questionnaire Personal Recovery, and the Childhood Bullying Questionnaire, and a qualitative interview (V4): Personal Experiences of discontinuation.

Visits phase II: Follow-up

Follow-up visits are scheduled after the discontinuation phase is completed, to assess the current status of the patient after that part of the study. There will be ten annual visits, the first scheduled 6 months after completion of phase 1 (thus one year after baseline), the others with intervals of one year (+/- 3 weeks). A qualitative interview to investigate existential factors of recovery is scheduled at V6, V8, V11, and V14. During the first Follow-up visit, patients will also receive a report on their individual study results that were obtained during Phase 1, including general functioning, cognition and physical health (further explained in K6.: Deelnemerrapport, dd February 2019).

6.4.1 Rating scales

- The World Health Organization's Disability Assessment Schedule (WHO-DAS-II) (Chopra et al., 2008) The WHO-DAS-II assesses disability in individuals irrespective of diagnosis across multiple life domains. The nature of disability is rated directly from individuals' responses. We will use the interview version consisting of 36 items.
- Side effects experienced by the use of mental health medications as assessed by the:
    - The Subjective Wellbeing under Neuroleptics (SWN): self-assessment scale of the subjective experience of patients during treatment with neuroleptics (Haan et al., 2002). The 20-item SWN scale has an internal consistency of 0.93 and the subscale consistencies ranged from 0.70 to 0.80. Test–retest reliabilities are observed of r = 0.70. Confirmatory factor analysis replicated the presence of a higher-order factor (general well-being) and five first-order factors (mental functioning, physical functioning, social integration, emotional regulation, and self-control).
    - The Subjective Reaction on Antipsychotics (SRA; Wolters, 2004) is a 73-item rating scale and consists of ten scales. Nine scales measures unpleasant effects: weight gain, sexual anhedonia, sedation, affective flattening, EPS, reduced sociability, increased sleep and total unpleasant effects. The last scale added the unpleasant scales, including the remaining items. The recovery scale measures the enjoyable responses attributed to the antipsychotics.
    - The Changes in Sexual Functioning Questionnaire (CSFQ, Clayton et al.,1997): while sexual dysfunctioning can be an important side effect of antipsychotic medication use, it is not often highlighted in research nor clinical practice. This 14-item self-rating questionnaire has a separate version for males and females.
- The EuroQoL (EQ-5D-5L; Sonntag et al., 2015) is a self-assessment questionnaire evaluating five health domains (mobility, selfcare, daily activities, pain and anxiety/depression) using 5 questions, which is needed for computing quality adjusted life years (QALYs).
- The economic costs of health care uptake, informal care, and productivity losses with the Trimbos/iMTA Questionnaire on Costs associated with Psychiatric illness (TiC-P; (Hakkaart - van Roijen et al., 2002).
- Internalized Stigma of Mental Illness (ISMI). Sub scales: alienation, stereotype endorsement, discrimination experience, social withdrawal and stigma resistance. Good internal consistency, test-retest reliability and construct validity. (Ritsher et al., 2003).
- Self-esteem Rating Scale- Short form (SERS-S; Lecomte et al., 2006)
- Psychosis Attachment Measure (PAM) is a 16-item self-rating scale measuring attachment anxiety and attachment avoidance and is a meaningful variable in people with psychosis and may be an important predictor of symptoms, interpersonal problems and difficulties in therapeutic relationships over and above severity of illness (Berry et al., 2008).
- Positive and Negative Syndrome Scale (PANSS) (Kay et al., 1987): this is a 30-item rating scale designed to measure severity of psychopathology in adult patients with schizophrenia. Five components have been reported: positive, negative, depression, agitation-excitement, and disorganisation.
- General functioning will be assessed using the GAF questionnaire (Jones et al., 1995).
- The Premorbid Adjustment Scale (PAS; Cannon-Spoor et al., 1982) is a rating scale about five domains of functioning: sociability, peer relationships, scholastic performance, adaptation to school and social-sexual aspects. It covers two life periods: up to 12 and 12 to 16.

- The Recovery Assessment Scale (RAS; Giffort et al., 1995): this is a 30-item rating scale designed to measure recovery. How people feel about themselves and their lives.
- The Multidimensional Scale of Perceived Social Support (MSPSS; Zimet, Dahlem, Zimet, & Farley, 1988) is a 12-item instrument that measures perceived social support. Four scales have been reported: significant other, family, friends and total scale.
- Aggressive and suicidal behaviour will be evaluated using a self-composed list of self-report questions (developed at the department of Psychiatry, UMC Utrecht.
- Cigarette, alcohol and drug abuse (as assessed with the alcohol and drug section of the Comprehensive Assessment of Schizophrenia: CASH; Andreasen e.a., 1992 and the WHO assist (Humeniuk et al., 2010))
- The MINI-Screener is a self-report questionnaire and consists of fife sections: generalized anxiety disorder, panic disorder, social anxiety disorder, obsessive compulsive disorder and post-traumatic stress disorder (developed at the department of Psychiatry, UMC Groningen).
- Childhood trauma will be evaluated by asking subjects to fill-in a retrospective self-report questionnaire, the Childhood Trauma Questionnaire-Short Form (CTQ-SF; Bernstein et al. 2003). The CTQ-SF is very short and easy to fill-in in approximately 5 minutes.
- Language production will be assessed by an automatic analysis of spoken language. Patients will be asked to answer questions on neutral topics; their answers will be recorded using a head-worn microphone. Patients will be asked to talk for approximately five minutes, with a maximum of ten minutes minutes. A set of open-ended questions will be used to elicit speech. If a patient does not wish to answer a particular question, it will be skipped. The speech recordings will be converted to writing using automatic speech recognition. The recordings will be automatically parsed and annotated using computer learning language systems.
- List of Threatening Experiences (LTE): a subset of 12 life event categories with considerable long-term contextual threat (Brugha et al., (1985). This list is very short and easy to fill-in in approximately 5 minutes.

6.4.2 Cognitive assessment

Neurocognitive functioning will be assessed at the baseline visit and at 6, 48, 84, and the 120 months visit with the BACS (Keefe et al., 2004) and the Stroop task (Stroop, 1935). The BACS is derived from larger neuropsychological batteries and provides a valid estimate of general cognitive ability. Those aspects of cognition found to be most impaired and most strongly correlated with outcome in patients with schizophrenia are assessed. The BACS has an expected administration time of 35 minutes and consists of the following subtasks:

- *Verbal memory - List learning:* Patients are presented with 15 words and then asked to recall as many as possible, which will be repeated five times. Measure: number of words recalled per trial, in any order.
- *Working memory - digit sequencing task:* Patients are presented with clusters of numbers of increasing length and are asked to tell them in order from lowest to the highest. Measure: number of correct responses.
- *Motor speed - Token motor task:* Patient are given 100 tokens and are asked to place them in a container as quickly as possible. Measure: number of tokens correctly placed into the container.
- *Verbal fluency - Category instances:* Patients are asked to name as many words in a certain category in 60 seconds (supermarket items, tools). Measure: number of unique and appropriate answers per category.

- *Verbal fluency - Controlled oral word association test:* Patients are asked to generate as many words as possible that begin with a given letter in 60 seconds. Measure: number of unique and appropriate answers per category.
- *Attention and speed of information processing - Symbol coding:* Timed paper-and-pencil test in which respondent uses a key to write digits that correspond to nonsense symbols. A sheet with a 9 item key is provided, pairing digit 1–9 with a unique symbol; below are rows of numbers with blank squares beneath. The subject pairs each number with its unique symbol. Measure: number of correct number-symbol pairs completed in 90 seconds.
- *Executive functions - Tower of London:* Patients are shown two pictures simultaneously with 3 pegs uniquely arranged in each picture. Patients are asked to give the number of times the balls in one picture need to be moved in order to make the arrangements identical on both pictures. There are 20 trials in with variable difficulty. Measure: number of correct answers.

In addition, the Stroop task will be administered, which measures executive functioning (verbal inhibition) and takes 3-5 minutes. Patients are shown three cards on which words are depicted in a 10x10 matrix. Card 1 contains color words (red, blue, yellow, green) that are printed in black ink in random order. Card 2 displays solid color patches in one of these four basic colors. Card 3 again contains color words, but these are printed in a incongruous ink color. Individuals were instructed to read the words (card 1), name the colors (card 2) and, finally, name the ink color of the printed words (card 3) in three subsequent sessions. Measure: reaction time and number of errors.

6.4.3 Movement disorders

Patients are examined for movement disorders during baseline, the close-out visit (6 months) and during each follow-up visit. A standard protocol will be used, as described by Van Harten and colleagues (1996). Patients are barefooted and seated in a chair without armrests. The researcher asks detailed questions about (i) use of chewing gum or candy at the moment of assessment as well as (ill-fitting) dentures, as both may be misdiagnosed as orofacial movement disorders, and (ii) subjective akathisia. The patient performs different tasks to assess the existence of movement disorders and to provoke abnormal movements. Thus, the following positions are adopted in succession: resting arms on the lap in different positions, arms hanging aside, stretching arms, making fast alternating hand and foot movements, opening the mouth, showing the tongue, rising from chair, and walking. Additionally, posture, rigidity and balance are assessed. Tongue dyskinesia is provoked by fingertip movements, and objective akathisia by talking conversationally while the patient is standing.

- Dyskinesia (American Psychiatric Association, 1992) is defined as hyperkinetic choreiform involuntary movements which often fluctuates in severity. Tardive dyskinesia is assessed with the St. Hans Rating Scale (SHRS; Gerlach et al., 1993) and case definition is based on Schooler and Kane criteria (1982), requiring (i) the presence of moderate dyskinesia in at least one body area or mild dyskinesia in at least two body parts, and (ii) the absence of other conditions resulting in abnormal involuntary movements.
- Parkinsonism is also assessed with the SHRS (Gerlach et al., 1993). A case definition of parkinsonism is based on (i) mild expression of rest-tremor or rigidity as both are typical of parkinsonism, and (ii) if no tremor or rigidity is rated, the cut-off point is one rating of moderate or two ratings of mild on items of bradykinesia and postural stability. The more stringent criteria for items of bradykinesia and postural stability are chosen as these symptoms may be part of psychiatric syndromes or sedation.
- Dystonia is defined as a syndrome of sustained muscle contraction, frequently causing twisting and repetitive movements or abnormal postures (Harten & Kahn,

1999). Tardive dystonia is diagnosed, following Burke's criteria (Burke, 1992), if one body area attracted a rating of at least mild or if two or more body areas attracted a rating of slight on the SHRS (Gerlach et al., 1993). As frequent eye-blinking (rating of 'mild' on the item 'eye') has many causes, case definition of tardive dystonia required a rating of at least moderate (blepharospasm) when 'eye' is the only symptom area.

- Akathisia (Factor et al., 2005) is defined as both subjective inner feelings of restlessness and objective motor (leg) movements. A case definition of akathisia is based on a rating of at least 'mild' on the global akathisia item. Akathisia is assessed with the Barnes Akathisia Rating Scale (BARS) comprising an objective and a subjective item (Barnes, 1989).

6.4.4 Additional measures

- Weight will be measured during each visit. In addition, abdominal and hip circumference, height and body mass index (BMI) will be noted. Blood pressure and pulse will be determined. This way, presence and severity of metabolic syndrome can be evaluated, as defined by the American Heart Association/National Heart, Lung and Blood Institute (AHA/NHLB; Grundy et al., 2005). The definitions and reference ranges for metabolic syndrome are:
  - Abdominal obesity (waist circumference) men ≥ 102 cm, women ≥ 88 cm
  - Triglycerides ≥ 150 mg/dL
  - High density lipoprotein (HDL-C) men < 40 mg/dL, women < 50 mg/dL
  - Blood pressure systolic ≥ 130 or diastolic ≥ 85 mmHg
  - Fasting glucose ≥ 100 mg/dL

According to the AHA/NHLB definitions, a diagnosis of metabolic syndrome is established if at least three of the above risk factors are present. Waist circumference, Body Mass Index and blood pressure will be measured at baseline, after 1 and 6 months and at the end (12 months) of treatment. Blood samples will be taken on these occasions to monitor triglycerides, HDL-C and fasting glucose.

- Medication use will be listed in a table, evaluating type of medication, reason for use, dosage (average dose per period), start and stop date, reason for stopping/switching of medication. Total history of psychopharmaca will be recorded by asking the patient, and if needed, complemented by the dedicated includer, other co-medication will only be noted for the past two weeks. Moreover, the treating pharmacy will be contacted for an overview of used pharmaceutical components during the duration of the study. Instead of contacting each pharmacy individually, we request data via the Foundation for Pharmaceutical Statistics (SFK).
- Use of psychoactive substances will be recorded. Frequency, quantity of use and start date of alcohol and cannabis will be scored. For other psychoactive substances only frequency will be scored.
- The assessor also calculates the total time the patient has spent with the treatment team since the last visit from the clinical file of the patient. These data are necessary for the health-economic evaluation, but they will also be used to control for potential bias of the single blind design, as treating teams may be inclined to spend more time and care with patients who are tapering off their medication, which may obscure effects of pharmacotherapy.
- The number of hospitalisations and relapses are also taken from the patient's file. Using information of the pharmacy, the mean cumulative dose of antipsychotic medication taken since baseline is calculated. Relapse is defined as clinical deterioration during at least 1 week, having consequences (augmentation of antipsychotic dosage, hospital admission, or more frequent consultations), reported by the clinician and subsequently confirmed by PANSS positive subscale item scores assessed by a research team member, of at least one score of 5 (moderately severe) (Wunderink, 2007). In contact with justice: number of times is noted.

- Involuntary admission for treatment: number of times and duration is noted.
- Skin autofluorescence: AGE (Advanced Glycation End products) levels in the skin will be measured four times, at V2 baseline (or during a later visit [V3-V7] in subjects already included), V8 (48 months), V11 (84 months), V14 (120 months). At each time point, a non-invasive autofluorescence measurement of the non-dominant forearm is performed three times. The total duration of the measurements is estimated at 2 minutes.
- Stool samples will be accompanied by three short questionnaires: the Bristol Stool Scale (BSS; Lewis & Heaton, 1997), the Gastro-intestinal Symptom Rating Scale (GSRS; Svendlund et al., 1988), the Food Frequency Questionnaire (FFQ; Sluik et al., 2016) and a single question regarding mode of delivery (caesarean section or vaginal birth).
- Psychological stress (PSS-10) and resilience (BRS) measurements are conducted at each visit, HRV measurement will be done during the visits in which blood and/or stool samples are collected.

6.4.5 Blood samples

Blood samples will be drawn at V2, V4, and follow-up V6 and V8. Refusal of providing blood samples is no reason for drop out of the study. During each occasion, one 6 mL EDTA will be drawn and frozen for determination of blood levels of the most frequently used antipsychotic medications (haloperidol, risperidone, olanzapine, quetiapine, clozapine and aripiprazol) and hormones, such as prolactin. This measurement provides a reliable indication of the treatment compliance of individual patients, although high-metabolisers of antipsychotics may show low levels. In addition, 1 lithium heparin tube (LHT; 3 to 4,5 mL) will be collected at these time points to assess the level of triglycerides, HDL-C (high-density lipoprotein cholesterol), fasting glucose, cholesterol, LDL and C-reactive protein (CRP) (an additional Sodium-Fluoride 2 mL will be drawn in the UMC Groningen or during home visits, to measure fasting glucose). As explained to the participants, these lab results are also sent to the treating physician and the general practitioner because the parameters may be clinically relevant, together with physical measures such as blood pressure (accompanying letter is added in F4: Brief Labuitslagen, dd February 2019). Also at V2, V4, V6 and V8 one tube (5 mL) for aliquotation of serum one will be drawn and at V2, V4, V6 and V8 one tube (3 ml) RNA stabilizing tube ("Tempus Tube", Fisher Scientific) will be drawn. Serum samples will be used to assess predictive risk profiles for relapse using proteomics analyses.
Furthermore, at baseline, one EDTA tube (10 mL) will be drawn and stored to enable DNA isolation. These tubes will be prepared by the laboratory of the relevant study centers and aliquots will be stored at -80^{0}C. After completion of the sample, genetic risk profiles will be assessed and used as predictors of relapse/successful discontinuation.
Participants are separately requested consent for storage of their DNA and blood for these future analyses, refusal of this blood storage will not prohibit further study participation (see Informed Consent form). Participants' blood products will be coded based on the participant identification number as used throughout the study. These codes will not be based on participants' dates of birth or initials, but instead are composed of two letters and three digit HAMLETT-specific code that cannot be traced back to the patient by outsiders. DNA and serum samples will be kept at the UMCU and UMCG in the research labs until completion of the study and specific analyses. Portions of serum and DNA may be shared with national or international research groups for specific analyses, when these analyses are not available in UMCU/UMCG.

6.4.6 Clinical safety measures

- *Clinical monitoring*: Professional caregivers will have regular contact with patients in both treatment conditions during inclusion in the trial. Patients, family members will be advised to contact professional caregivers as soon as possible even when they are

only slightly worried. Professional caregiver or someone who can replace him or her will be available to see the patient at least within two days after someone communicates his/her concerns (responsibility lies with the treating physician). When necessary, evaluation on the same day will be arranged.

- *Screening of early warning signs*: Directly after inclusion in the trial, professional caregiver together with patient and family members will be advised to create a personal patient profile in which individual early warning signs of impeding relapse are described. These signs are the individual prodromal signs patient experienced before their first psychotic episode. Patients and family members will be instructed to contact professional caregivers in case of occurrence of early warning signs. Patients also receive an information booklet about discontinuation of medication (further explained in K6.: Afbouwgids, dd February 2019)
- *Involving a family member or other informal caregiver:* If the patient has an involved family member, partner or roommate, he/she is invited to join the patient on the visits and his/her opinion is asked as to whether the patient is stable, getting better or worsening. In the case of worsening, treatment will be intensified and medication dose may be increased.
- *Treatment modification in case of occurrence of early warning signs or other indication of clinical worsening*: In case early warning signs occur, further tapering of antipsychotic medication will be halted until early warning signs disappear. Professional caregiver will contact the patient regularly. Stress reduction will be advised. When early warning signs disappear, tapering of antipsychotic medication can be resumed. When early warning signs become more severe, the dosage of antipsychotic medication will be increased to one level higher [in other words: back to the former step] of the tapering scheme. When psychotic symptoms occur, treatment with antipsychotic medication will be restarted in the dose that patients used when the first symptomatic remission occurred. When dangerous or disruptive behavior occurs, clinical treatment may be necessary.
- *Documentation of occurrence and severity of signs and symptoms and treatment modification*: Will be assessed during each visit.
- *Dispensing medication*: Antipsychotic medication will be prescribed by the treating psychiatrist for a maximum duration of 4 weeks. Patients and their involved family member will be advised on the tapering or continuation scheme [depending on the condition and depending on occurrence of early warning signs or psychotic relapse] each 4 weeks.

6.5 Ecological Momentary Assessments

Ecological Momentary Assessments (EMA) is a structured diary method in which individuals are asked in normal daily life to report their thoughts, feelings and symptoms, as well as the context (e.g. location, company, activity, substance use) and the appraisal of the context (e.g. stress) (Myin-Germeys et al., 2009). When the diary is filled out several times a day over several consecutive days, a detailed ‘film’ of daily life can be captured, consisting of an intensive time series of psychopathology, subjective well-being and social functioning in relation to antipsychotic medication and a range of contextual influences (Os et al., 2013; 2014a). In this study, EMA will be used in two ways:

1) EMA as a measure of social functioning and well-being in daily life

The key question of this project is whether discontinuation of antipsychotic medication will lead to better long-term social functioning. The in-the-moment, multiple, real world EMA is likely to give insight in objective and subjective social functioning in daily life in a way that is not captured in questionnaires addressing aspects of functioning retrospectively over a certain period of time (Bos et al., 2015; Os et al., 2014b). Therefore, EMA will be used as a secondary outcome measure of social functioning and well-being.

Design

Momentary positive/negative affect, self-esteem, subjective well-being, paranoia, hallucinations, sleep, and frequency, type and appraisal of social company and activities are assessed on a 1-7 scale via a smartphone diary app. At baseline and after 6 months, 1, 2, 3 and 4 years follow-up, patients of both arms will perform EMA for eight consecutive days at eight semi-random moments during the day.

Objectives

i) To investigate whether discontinuation of antipsychotic medication is related to increase in social activities, more positive appraisal of social interactions and higher level of well-being; ii) to determine whether discontinuation is associated with better short- and long-term social functioning and well-being than treatment as usual.

Statistical analysis and sample size:

Mean scores of the separate EMA items will be compared between the discontinuation and the TAU groups at each time point of follow-up, taking into account the multilevel structure of the data (repeated measures within individuals at multiple time points). As there are no longitudinal EMA discontinuation studies of antipsychotic medication, the best available estimates of differences in EMA mean scores between both groups may be differences in EMA item scores between psychotic patients and remitted patients. A previous EMA study found effect sizes between 1.3 (for momentary paranoia) and 0.5 (for event stress), with an average standard deviations (SD) around 0.85 (Collip et al., 2011). With a power of 80% and type I error of 0.05, a sample size of 90 (45 in each group) is required to detect an effect of 0.5. This multilevel study contains 288 measurements per patient (6x8x6), of which we estimate that 65% will be completed. Assuming 187 measurements per patient and an intra-class correlation of 0.8 within individuals, the sample size can be reduced to an effective n of 80. We aim to include an extra 10% to adjust for drop-out, thus 88 patients in total. In order to recruit this sample, six centers will be asked to include the EMA package.

2) EMA as 'magnifying glass' for detection of early clinical changes during discontinuation.

Using EMA it is possible to detect early clinical changes associated with medication reduction. Also, it is possible to detect early signs of psychotic relapse before the actual occurrence, for example in the form of 'critical slowing down' of EMA-level psychotic experiences. 'Critical slowing down' refers to the phenomenon that deviations from baseline take progressively longer to return to their original value, indicating that the system in question is increasingly fragile and close to a critical transition such as a relapse psychotic episode. Statistically, these early changes are apparent in changes in the autocorrelation between successive observations in the intensive time series. Previous studies have shown that this is possible for the reappearance of depressive symptoms (Leemput et al., 2013; Wichers et al., 2016).

Design

A sample will be recruited, consisting of 30 patients wishing to discontinue their medication and at currently at a sufficiently low dose to do so within the proposed period, who are motivated to monitor their momentary daily life experiences using a smartphone EMA app 10 times a day (in order to build the required intensive time series) for a period of 16 weeks, over which the moment of initiation of tapering is started after 4-7 weeks of EMA (depending on patient preference) whilst on medication. Momentary mental states will be assessed in terms of psychotic experiences, positive affect, negative affect, stress-reactivity, reward experience, social interactions, activities and substance use.

Objectives

i) to determine the clinical effects of dose reduction of antipsychotic medication under longitudinal self-monitoring by EMA, capturing intensive within-person time series of mental states and social context in the flow of daily life; ii) to analyse to what degree early changes in the dynamic system of mental states predict future clinical change (improvement or deterioration); iii) to meta-analyse results of 30 n=1 experiments, yielding knowledge at the group level that can potentially be used at the level of general guidelines; and iv) to develop a feedback tool that allows patients and clinicians to collaboratively titrate individual optimal dosages in normal clinical practice.

Data points and Power-analysis

Each patient will provide EMA data for a total of 16 weeks. Since there will be 10 measurement occasions per day, in principle this implies a total of 16×7×10=1120 measurements per patient. Prior EMA studies with psychosis patients (n=226) indicate that about 70-75% of beeps are responded to in time. We conservatively estimate that, on average, 728 measurements (65%) will be available per patient. The same prior EMA data can also be used to get an indication of the within-subject variability and autocorrelation in the data. Focusing on negative affect (NA) as the outcome measure (the average of 6 items, each measured on a 1-7 scale), we find a within-subject SD of 0.63 and an autocorrelation of 0.26. We distinguish the phase prior to the dose reduction and the phase after dose reduction starts. On average, the first phase will be about 5.5 weeks long (dose reduction will start after 4-7 weeks) and therefore contains 5.5x7x10x.65=250 measurements. On average, the phase after the dose reduction starts will be 10.5 weeks long and therefore contains 10.5x7x10x.65=478 measurements per subject.

The goal is to detect a change in trend and overall level from one phase to the next. For the power calculations below, we assume that NA levels are, on average, stable prior to the dose reduction phase, but the crucial aspect here is the change in the angle of the slope. Furthermore, we assume that once the dose reduction starts, either an increasing or decreasing trend manifests itself in the data. Such a shift in trend can be captured via an appropriately formulated linear model, allowing for auto-correlated residuals. We assume an increase/decrease in the slope by .001 points during the dose reduction phase. Over the 10.5 weeks, this would imply a total increase/decrease in NA levels by 10.5×7×10×.001=.735 points or a change of .37 in the overall level (mean) when comparing the pre- to the post-reduction phase. In terms of an effect size, the change in the overall level corresponds to a standardized mean change of .37/.63≈.58 (i.e., mean change relative to the within-person SD), which can be considered a medium-sized effect.

6.6 BeHapp

General description

BeHapp is a smartphone application which is developed for the direct day-to-day registration of several aspects of daily life concerning the behaviour of humans. The application continuously monitors communication and exploration patterns in patients and controls as a function of social acts, environment social density measurements, gps location updates and general smartphone usage. This method circumvents the issue of subjectivity given that no active input is required from the participant, other than installing the application and providing some baseline information.

Methods

The application, once installed and initialized, passively collects (meta) data about phone call activity, bluetooth devices and WiFi access points in the direct vicinity of the participant, location updates and mobile application usage. By means of integrating this data a multidimensional behavioral profile of the participating individuals can be obtained.

The application is currently only compatible with the Android platform. Each participant will be asked if they use an Android smartphone and if they consent to this part of the study. After thoroughly explaining what the application does, the participant will be provided with an e-mail message containing instructions on how to obtain and initialize the application. Initialization consists of entering a (single-use) shortkey to identify oneself to the application, this key is also provided in the aforementioned e-mail message. If preferred by the participant this process can be completed with our help as well.

Privacy
Several measures are taken so as to protect the privacy of our participants:

- As a base principle, no directly identifiable information about any participant is stored within any system that is part of the BeHapp service. Participants are represented by unique id's, which are associated with identifiable information in decentralised third party information systems.
- In order to decrease the attack potential ('attack surface') of the BeHapp service, the publically accessible centralised software components are designed and implemented to work with least privileges and least programming logic. By this we aim to prevent retrieval of participant data through the application layer of our service.
- Monitoring data is encrypted before being stored locally on the device of a participant and after each successful upload, all of the local monitoring data is cleared from the device. Bother measures are in place to maximally reduce risk of revealing privacy-sensitive information to third parties in case of loss or theft of the smartphone.
- All communication streams between the mobile applications and our centralized data storage components will go through secured (encrypted) channels using modern industry standards.
- Information gathered about identities of third persons, e.g. contacts of participants, are obfuscated on the phone before being sent to the central data store. Obfuscation is performed using so called one-way-hashing / encryption techniques. This allows the researcher to determine whether the same instance (person or device) has been recorded more than once while preventing them from directly identifying the recorded instance.
- The BeHapp service resides within the Google Cloud Platform which employs a security model built on 15 years of experience in protecting customer data. The platform is compliant with HIPAA, ISO 27001 and the EU Data Protection Directive. Furthermore, all data stored is encrypted by default ensuring that only researchers part of the BeHapp team are able to access the data.
- In order to reduce the attack potential ('attack surface') of the BeHapp service, the publically accessible centralised software components are designed and implemented to work with least privileges and least programming logic. By this we aim to prevent retrieval of participant data through the application layer of our service.

Duration of measurement
The duration of measurement, i.e. the time that BeHapp actively collects and uploads data on the participant, is variable and may depend on the specific strategy and aims of the study it is employed in.
At any time during the course of the study, you can decide to stop BeHapp from collecting information by stopping the application in the application manager (and re-activating it when desired) or removing the application permanently from your device.

6.7 Raclopride PET

Discontinuation of antipsychotic medication after the use of these drugs for several months may render patients especially vulnerable to relapse. Potential mechanism behind this

vulnerability could be increased density of postsynaptic dopamine D2 receptors in the striatum. To date, there are no clinical studies examining whether relapse in FEP patients following antipsychotic withdrawal is linked to dopamine D2 receptor changes. An animal study conducted by Joyce (2001), who treated rats with haloperidol for 9 months showed that an increase in D 2 receptors persisted for at least 2 months after withdrawing from antipsychotic medication. In human equivalent, this would imply a period of increased vulnerability to develop psychotic symptoms following discontinuation of antipsychotic medication for more than 1 year (Quinn, 2005). Male FEP patients participating in the HAMLETT study will be asked to participate in this PET (positron emission tomography) study. A total of 30 patients will be included. The aim of this study is to investigate the presence of dopaminergic abnormalities, as measured with [11C]raclopride, in relation to antipsychotic medication discontinuation in patients remitted after a first-episode psychosis (FEP).

The subjects will undergo a PET scan with [11C]raclopride, which is a marker for the availability of dopamine D2 receptors in the striatum. A separate protocol for this study will be submitted to the METC Groningen.

6.8 MRI and OCT study

Previous research has reported a negative association between cumulative dose of antipsychotic medication and brain volume in patients with schizophrenia. A problem with the studies reporting on cumulative dose of antipsychotic medication and brain volume loss, is that patients with severe symptoms tend to need a higher dose than patients with mild symptoms. As patients with mild symptoms have a better outcome, the association is likely to be confounded by disease severity. The aim of the proposed study is to investigate the effects of annual cumulative antipsychotic dose on specific structures such as hippocampus, thalamus, caudate, parietal and prefrontal cortex. We will investigate gender effects and effects of type of medication. Furthermore, we will associate brain volume loss between the two scans to short and long-term clinical outcome of the patients.

In addition, Optical coherence tomography (OCT) will be investigated. OCT has become the most important instrument for the assessment of the retinal nerve fiber layer (RNFL) thickness, which is relevant to common eye diseases like glaucoma. OCT is also emerging as a biomarker for neurodegeneration in general. With the development of advanced segmentation algorithms, it is now possible to assess the thickness of all individual retinal cell layers (RNFL, ganglion cells, amacrines, bipolar cells, horizontal cells, photoreceptors, and pigment epithelium) in a few seconds. This has furthered the understanding of eye diseases tremendously, and offers opportunities to better understand other diseases that affect the nervous system. OCT uses a weak, infrared light source that doesn’t harm the eye or even causes discomfort. Making an OCT scan resembles making a picture of the retina, but without the annoying light flash needed for conventional photography. The subject will be asked to place their head on a chin-rest in front of the OCT system, and then to focus on a fixation cross. Two pictures will be taken of the subjects retina, one of the fovea and one of the optic nerve head. These images will show the thickness of the subjects’ retina. Thinning of the retina is indicative of glaucoma. All measurements will be performed using non-contact methods. No eye drops will be provided to subjects or patients. An OCT measurement itself takes only a few seconds. Including instructions and explanation, it takes less than 10 min. This includes three images for each eye of which the median value for each parameter will be used for analysis

FEP patients participating in the HAMLETT will be asked to participate in this MRI and OCT study. A total of 150 patients (75 in each arm) will be included and scanned twice with 11 months in between measurements. Between the two scans, half of these patients have tapered of medication gradually, the other half have continued on medication. The main study parameter is brain volume measured by MRI and thickness of the subjects’ retina as measured by OCT. A separate protocol for this study will be submitted to the METC Groningen.

6.9 Personal recovery and qualitative interviews

Well-being is more than the absence of psychiatric symptoms and involves positive aims and contentment, meaningful engagement (personal relationships) and contribution to society (social engagement). Because of a psychotic disorder, people often have to adjust their aims and perspective of the future changes. How this process of recovery develops is very personal, as is the support and treatment given by professionals. When it comes to symptomatic recovery and societal recovery, more and more interventions are available. This is less clear when it comes to existential recovery. Some patients report existential recovery without having clinical and societal recovery, while other patients function at a high level and experience few or no clinical symptoms but still do not report personal recovery. There is hardly any knowledge about this connection and factors that can influence existential recovery in particular. Some studies have touched upon this topic, without performing longitudinal measurements. For example, Whitley and Drake describe existential recovery as having a sense of hope, empowerment agency and spiritually well as wellbeing (Whitley et al., 2010). These factors may allow a person to feel more in contact with their lives and less subject of their illness. Data on course and outcome in the domain of existential recovery is completely absent, despite this being a crucial domain for patients (Boevink et al., 2016, 2017). The aim of adding attention to existential recovery is to explore the lived experiences on how existential recovery is shaped over time in patients after a first episode psychosis and what aspects were hindering and stimulating this process. Based on these insights we can improve daily treatment and develop adequate interventions. The software program ATLAS.ti, version 8.1.0 (ATLAS, 2019) is used as a tool for analyzing the data.

A subset of patients will be asked to participate in an interview 4 times during a period of 8 years, 2 years after the first psychosis, 4 years, 7 years and 10 years after the first episode psychosis. We will follow 40 patients for this part of the study (approximately 1 or 2 from each participating center); this seems justifiable because it is a very intensive method focusing on exploration and not on generalizability. We will vary as much as possible with regard to gender, age, educational level, area (rural/urban), and ethnicity in order to include a patient group that is as heterogeneous as possible. We think we need about 20-25 patients to achieve data saturation, because of the long follow-up of ten years we calculate a drop-out of about 40% of the patients. The research team consists of a PhD student who will be assisted by an expert by experience. An expert by experience is part of the research team to examine the focus and content of the kit for the probe, to discuss insights and to think about the interpretation. An experiences researcher on qualitative research will supervise this part of the study. All interviews will be audiotaped and transcribed verbatim. Using member check, the obtained data is presented to the client in order to increase reliability. We will use thematic analysis to analyze subtle nuances in meaning from the qualitative data (Braun & Clarke, 2006). Bracketing by maintaining a reflective log file is common in the phase of data analysis. During this phase, peer debriefing will be conducted following each step in the coding process. The thematic analysis is carried out using an inductive (data-driven rather than theoretical) approach. Each theme will be described in the findings, and notable quotes will be used to clarify the findings.

7. SAFETY REPORTING

This study will be performed according to the Declaration of Helsinki (64th WMA general assembly; October 2013) and the International Conference on Harmonisation – Good Clinical Practice (ICH-GCP). The definitions of adverse events and serious adverse events described in these guidelines will be used for the present study. In accordance to section 10, subsection 1, of the WMO, the investigator will inform the subjects and the reviewing accredited Ethical Review Board (ERB) if anything occurs, on the basis of which it appears that the disadvantages of participation may be significantly greater than was foreseen in the research proposal. The study will be suspended pending further review by the accredited ERB, except insofar as suspension would jeopardise the subjects' health. The investigator will take care that all subjects are kept informed.

7.1 AEs, SAEs and SUSARs

Adverse events (AEs) are defined as any undesirable experience occurring to a subject during the study. All adverse events reported by the subject or observed by the investigator or his staff will be recorded.
A serious adverse event (SAE) is any untoward medical occurrence or effect that:

- results in death;
- is life threatening (at the time of the event);
- requires hospitalisation or prolongation of existing hospitalisation;
- results in persistent or significant disability or incapacity;
- Any other important medical event that may not result in death, be life threatening,

or require hospitalization, may be considered a serious adverse experience when, based upon appropriate medical judgement, the event may jeopardize the subject or may require an intervention to prevent one of the outcomes listed above.
The sponsor will report the SAEs through the web portal *ToetsingOnline* to the accredited ERB and Competent Authority that approved the protocol, within 15 days after the sponsor has first knowledge of the serious adverse reactions. Additional study sites will report SAEs in the eCRF and notify the sponsor. SAEs that result in death or are life threatening should be reported expedited. The expedited reporting will occur not later than 7 days after the responsible investigator has first knowledge of the adverse reaction. In case additional information is required, this will be provided through an update report within the next 8 days (within 15 days in total).

Hospitalisation due to exacerbation of psychosis-related symptoms is a very common occurrence during the first years of the illness. Although this is regarded as being an SAE, these hospitalisations are part of the illness course and are therefore not reported to the authorities immediately. Rather, they will be reported once a year as part of the Annual Safety Report. Immediate reporting will not have any added value for the authorities in evaluating patient's safety and will result in over-reporting.

7.2 Annual safety report

The sponsor will submit, once a year throughout the clinical trial, a safety report to the accredited ERB and competent authority.
This safety report consists of:
i) a list of all suspected (unexpected or expected) serious adverse reactions, along with an aggregated summary table of all reported serious adverse reactions, ordered by organ system, per study;
ii) a report concerning the safety of the subjects, consisting of a complete safety analysis and an evaluation of the balance between the efficacy and the harmfulness of the medicine under investigation.

7.3 Follow-up of adverse events

All AEs will be followed until they have abated, or until a stable situation has been reached. Depending on the event, follow up may require additional tests or medical procedures as indicated, and/or referral to the general physician or a medical specialist.
SAEs need to be reported until the end of the study. However, should the investigator become aware of an SAE or SUSAR that occurs within 30 days after stopping the study treatment, the event must be reported in accordance with procedures specified above.

7.4 Data Safety Monitoring Board

It has been decided not to engage a Data Safety Monitoring Board in this study. Patients will be extensively monitored during the study. In addition, interim analyses are planned to assess if one of the trial’s conditions (either discontinuation or continuation) is associated with markedly inferior outcomes. Interim analyses will be performed after 1 and 3 years by an independent statistician. In the investigator’s opinion, implementation of a DSMB will not have sufficient added value for the current study.

8. STATISTICAL ANALYSIS

8.1 Lost to follow-up and data-management

Drop-out criteria are kept as few as possible. Only patients who cannot be traced anymore, who have died or who refuse any further visits or withdraw their informed consent will be considered to have dropped out. Previously collected data from patients who drop out are kept in the analyses. Patients who can be traced on a later stage or who change their mind after leaving the study are welcome to be followed-up again.

We expect that many patients from the discontinuation arm may taper off their medication more slowly or rapidly than indicated in the schedule. After medication is stopped, they may restart medication or start another antipsychotic drug for a short or longer duration to prevent relapse at some point during the follow-up.

Likewise, in the continuation group, we expect several patients to switch to another antipsychotic drug, change dose or stop medication earlier than indicated (i.e. before the first year is finished). As this is a pragmatic trial, we keep all patients in the follow-up and analysis, even if they no longer meet the requirements for the condition towards which they were randomized. This has the advantage of providing information on all patients that follow any possible route and thus reflecting daily practice. It also provides the power needed to make a final recommendation on the (dis)advantages of discontinuation of maintenance treatment. This strategy will have the disadvantage that the differences between the two arms may be smaller. We will control for this by calculating the mean cumulative dose of antipsychotic medication used in each arm during the total period of the study. The intention to treat analyses also provides the possibility to asses feasibility of the discontinuation and continuation interventions.

We expect a retainment rate of 90%. The visit team will invest on personal contact (visits with same researcher, extra time to improve personal contact) to maximize the retainment rate.

8.2 Interim analyses

Interim analyses are planned to assess if one of the trial's conditions (either discontinuation or continuation) is associated with markedly inferior outcomes. Interim analyses will be performed after 1 and 3 years by an independent statistician. Prof. dr. Pal Czobor (Semmelweis University, Department of Psychiatry and Psychotherapy, Budapest) kindly agreed to assume this role. The interim analyses will be conducted for the primary efficacy end point of the study obtained from patients in the target population. The statistical analyses will be carried out at the two-sided overall alpha-level of 0.05. The Type I error boundaries for statistical significance will be adjusted for multiple comparisons (i.e., total number of analyses=3). A design based error spending function using the O'Brien-Fleming boundaries will be applied (O'Brien & Fleming, 1979): A multiple testing procedure for clinical trials (Biometrics, 35: 549-556). The O'Brien-Fleming plan allocates the alpha error conservatively across the interim and final analyses in the study. At the first interim analysis, a two-sided p-value will be declared significant if it is less than 0.0021; at the second interim analysis, the respective alpha error boundary will be 0.0105. At the final analysis, the two-sided p-value will be declared significant if it is less than 0.025. Based on the outcome at the interim stage (i.e., if $p<0.0021$ or $p<0.0105$, for the two interim analyses, respectively), the study can be stopped for overwhelming evidence of group difference.

In order to perform the interim analyses, the study management provides the independent statistician with a clean copy of the relevant clinical data in the form of a downloadable static version of the database. Since the interim analysis may lead to termination of a trial, the data on which this decision is based will be as clean as possible to avoid any question of an erroneous basis for decisions. At the planned date stipulated for the interim analysis, the statistician who conducts the analysis will download an electronic version of the database. Furthermore, the statistician receives a copy of the treatment assignment code directly from the independent person who stores and maintains the treatment assignment codes for the

study. The statistician applies the code to the pertinent data from the trial and conducts the analyses described above. The database used for the analysis must be archived to provide a clear audit trail as well as to allow for future re-analyses for regulatory purposes. The interim report will not include quantitative information summarized by treatment group (not even masked generically as group A or B) nor any other information that would compromise the blinded-analyses. The statistician has an advisory role; s/he is not empowered to stop the trial. Ultimately, it is the PI' responsibility to decide whether to continue or discontinue a trial based on the results of the interim analysis. Communication between the study management and the independent statistician will be documented, and full details of the analyses will be included in the final report of the study for a subsequent regulatory assessment of the trial.

8.3 Analyses of clinical outcome

Linear mixed modelling (for continuous outcomes) and multilevel linear mixed-effects logistic regression modelling (for binary outcomes) will be used to test the hypotheses mentioned under Objective 1, while adhering to the intention to treat principle and taking into account the clustered data structure of patients being nested in treatment centres. These analyses will be conducted for both the primary outcome of the trial (social recovery), secondary outcomes (subjective wellbeing) and possibly adverse outcomes (metabolic syndrome; aggressive incidents; psychotic relapse; hospital admission). Outcomes will be reported as stipulated by the CONSORT statement. As we use a single-blind design, it is possible that one arm (the one tapering off medication) will receive more care than the other one (as physicians may be worried about this group). To investigate such potential bias, the linear mixed model will use total clinical care received (in # hours) as a covariate.

8.4 Health-economic evaluation

The health-economic evaluation will be conducted in four different but complementary ways, each addressing a distinct research question.

1. A *cost-effectiveness analysis* (CEA**)** will be conducted with costs stemming from health service uptake and productivity losses and recovery as the central outcome.
2. A *cost-utility analysis* (CUA) with incremental costs per quality adjusted life year (QALY). QALYs will be based on the EQ-5D-5L and Dutch tariffs. The CEA and CUA will be conducted from both the health care and societal perspective as outlined by relevant CONSORT and CHEERS guidelines i.e. following the intention-to-treat principle and discounting costs and effects when the time horizon exceeds 1 year. Stochastic uncertainty will be handled using 5000 non-parametric bootstraps and by plotting the simulated incremental cost-effectiveness ratios (ICERs) on the ICER plane. For decision-making purposes, the ICER acceptability curve will be graphed along a range of willingness-to-pay (WTP) ceilings in order to judge if dose reduction and discontinuation is acceptable from a cost-effectiveness point of view.
3. An *incremental net-benefit regression analysis* (INBRA) will be used for prognostic modelling of net-benefits defined as Net-Benefit = Effect x WTP – Costs. INBRAs will be bootstrapped and will be repeated across a range of WTP values.
4. Finally, *budget impact analysis* (BIA) will address the question how the public purse and health care insurers' budgets will be affected when the intervention is implemented in 25, 50, 75 and an extreme 100% of the intended target group. For this, we will use our Markov model for first psychosis, PsyMod, which can conduct BIAs at a national level over a time horizon of up to 10 years.

COST-EFFECTIVENESS AND COST-UTILITY ANALYSES

CEA and CUA: General considerations

Both the CEA and the CUA will be conducted in agreement with the latest Dutch guideline on health-economic evaluation (Zorginstituut, 2016), hence from the societal perspective and in agreement with the intention to treat principle. Sensitivity analyses will be directed at uncertainty in the main cost-drivers and outcomes.

PICOT

P: Population: patients with a first-episode psychotic episode in stable symptomatic remission over 3 months on antipsychotic medication.

I: Intervention: well-tapered dose reduction possibly leading to discontinuation of antipsychotics.

C: Comparator: usual care consisting of continuation of antipsychotic medication over at least one year post symptomatic remission as per the current clinical guidelines.

O: Outcomes: In the cost-effectiveness analysis, CEA, the outcome is functional recovery as measured with the WHO-DAS-II. In the cost-utility analysis, CUA, the outcome is quality adjusted life years (QALYs) based on the EQ-5D-5L using Dutch tariffs.

T: Time horizon: The CEA and CUA are conducted alongside the randomized trial which has measurements up to five years post baseline.

CEA and CUA: Cost calculations

We will consider four types of costs: (1) intervention costs, (2) costs stemming from health care uptake, (3) patients' and their family's costs for travel and informal care, (4) costs stemming from productivity losses due to absenteeism and lesser efficiency while at work and owing to changes in the contractual number of work hours per week, both in paid work and volunteer jobs.

Data on resource use (health care uptake), informal care, travel distances to health services, and productivity losses will be collected with the latest version of the Trimbos/iMTA Questionnaire on Costs associated with Psychiatric illness, TiC-P, (Hakkaart - van Roijen et al., 2002). The TiC-P is the most widely used health service receipt interview for economic evaluations in the Netherlands. Total costs will be estimated using a bottom-up (or micro-costing) approach, where units of health service are multiplied by their appropriate unit cost price and summed to provide an overall total cost estimate (Drummond, Sculpher et al., 2005). We shall make use of the standard unit cost prices as reported in the latest Dutch guideline for health economic evaluation (Zorginstituut Nederland, 2016, or later editions). Costs of medication (and dispensing costs) will be calculated using prices based on Daily Defined Dosage (DDD) taken from the Dutch Pharmacotherapeutic Compass (www.farmacothera-peutischkompas.nl and www.medicijnkosten.nl), indicating the mean medication usage per adult a day including claw back (by the government imposed discount for patients, paid by the pharmacy). In the context of the dose-reduction trial, we will include the costs of tapering strips and the pharmacist's cost of distributing these to the patients, plus the costs of psychiatrists for clinical management of dose reduction and discontinuation. Productivity losses will be based on the friction cost method as per the Dutch guideline. Furthermore, the manual recommends that prices of informal care will be based on shadow prices for unpaid work (meaning a standard cost price based on general hourly wages). Costs of transport will be calculated as the mean distance per destination multiplied by standard cost prices. All costs will be expressed in 2017 euros. If necessary, existing cost prices will be updated to 2017 using the consumer price index.

CEA and CUA: Outcomes

The CEA and CUA will be conducted alongside the randomized trial. For the CEA, the central clinical end-term will be functional recovery as assessed by the WHO-DAS-II. It is worth noting that for the CEA's sensitivity analysis we will also define functional recovery more stringently, i.e. with the additional provision that patients who made it through functional recovery on the WHO-DAS-II should not experience significant deterioration on the following of trial's secondary clinical outcomes: SWNS, BACS and St Hans rating scale. For the CUA, the Dutch tariffs (utility weights) of the EQ-5D-5L will be used for computing QALYs (cf. www.euroqol.org). The EQ-5D-5L is chosen because it is a widely used quality of life instrument, both nationally and internationally. The EQ-5D-5L contains 5 dimensions of health-related quality of life: mobility, self-care, daily activities, pain/discomfort and depression/anxiety. Each dimension can be rated at five levels: from no problems to major

problems. The 5 dimensions can be summed into a health state. Utility values can be calculated for these health states, using preferences elicited from the Dutch population (Versteegh et al., 2016).

CEA and CUA: Analysis

The comparability of groups at baseline will be assessed for both costs and outcomes. When necessary, methods will be applied to control for baseline differences (Manca et al., 2005; Asselt et al., 2009). Missing cost and outcome data will be imputed using estimation-maximization (EM), regression imputation (RI) and last-observation carried forward (LOCF) for intention-to-treat (ITT) analysis. Since the trial's follow-up measurements exceed one year, both costs and effects will be discounted by 4% and 1.5%, respectively. Cumulative costs and health gains over the study's follow-up period will be computed with the area under the curve method to obtain an estimate for WHO-DAS-II recovery and cumulative QALY health gains as accrued over the measurements up to five years post baseline. The incremental cost-effectiveness ratio (ICER) will be computed to obtain the incremental costs per functional recovery and the incremental costs per QALY gained. Stochastic uncertainty will be handled using 2,500 non-parametric bootstraps and by plotting the simulated ICERs on the ICER plane. For decision-making purposes, the ICER acceptability curve will be plotted for various willingness-to-pay (WTP) ceilings for making judgements whether discontinuation offers good value for money relative to routine medical care. One-way sensitivity analyses directed at uncertainty in the main cost drivers (e.g. costs of hospital re-admissions after psychotic relapse) and outcomes (e.g. under different imputations) will be performed to assess the robustness of our findings. Both the analysis and reporting of the research findings will conform to the (extended) CONSORT and CHEERS statements (Schulz et al., 2010; Campbell et al., 2012; Huserau et al., 2013).

BUDGET IMPACT ANALYSIS

BIA: General considerations

The budget impact analysis (BIA) will be conducted as outlined by the ISPOR Task Group i.e. Mauskopf et al. (2007) and Sullivan et al. (2014) to assess how health care budgets change when antipsychotic dose reduction/discontinuation is offered over a range of implementation levels. The BIA will be conducted from various perspectives: (1) the societal perspective, i.e. the costs of (informal) care plus costs stemming from productivity losses; (2) the perspective of the public purse (in Dutch: Budgettair Kader Zorg); and (3) the perspective of the health care insurer. In each perspective the following scenarios will be assessed: a scenario in which the intervention is offered to 40%, 60% and 80% of the target group and an extreme scenario in which 100% of the target group will be receiving the dose reduction intervention. These scenarios will be compared with a base-case scenario where 0% of the target group is offered dose reduction / discontinuation (reflecting the current guidelines for psychosis).

BIA: Modelling approach

The BIA will be based on a health-economic simulation model for psychosis (PsyMod; Thielen et al., in prep.), which is an incidence-based Markov cohort model recently developed at Trimbos Institute. The development of the health economic model was based on modelling techniques outlined in Briggs et al. (2006) and in accordance with the recommendations from ISPOR-SMDM Modeling Good Research Practices Task Force-2 (Roberts et al., 2012), starting off with scoping the problem, conceptualizing the model, gathering evidence from the relevant sources and testing the model with the model users and stakeholders. PsyMod compares costs between two scenario's: a base-case scenario representing usual/guideline congruent pharmacological antipsychotic maintenance therapy and an alternative scenario representing well-tapered dose-reduction/discontinuation. Cost data will be extracted from the trial. Costs will be modelled out over the short term (12 months) and longer-term (36 months) as required by the Dutch guideline for health-economic evaluation (Zorginstituut Nederland, 2016). Long-term costs will be discounted according to the Dutch guidelines. PsyMod can conduct extensive uncertainty analyses over all cost and discounting parameters simultaneously and these will be reported accordingly.

NET-BENEFIT ANALYSIS FOR PROGNOSTIC MODELLING

NBA: General considerations

The intervention might be more cost-effective in some population segments, but less so in other segments. For example, smaller treatment effects and lesser cost-effectiveness of the intervention might be expected in patients presenting with higher levels of paranoia, comorbid axis-2 disorders and in ethnic minorities. Moderator analysis (of effect modification) is often used to shed light on heterogeneity in treatment response owing to patient population diversity. In the context of health-economic evaluations, moderator analysis is conducted using incremental net-benefit regression analysis (INBRA) to address the question "Who benefits most?" Answering this question helps to optimise matching the dose-reduction intervention to patients in a cost-effective way.

NBA: Prognostic modelling of net-benefits

INBRA relies on the same data as the CEA and CUA. INBRA is essentially a regression analysis were (1) the treatment dummy, (2) a prognostically relevant population characteristic (e.g. Dutch vs non-Dutch cultural background) and (3) their interaction are regressed on net-benefits (Hoch, 2009). Net-benefits, NB, are defined as, $NB = (E * \lambda) – C$, where E are effects (changes in QALY), λ is generally an unknown quantity representing the willingness to pay (WTP) for one unit of health gain (such as gaining one additional QALY) and C are the per-patient sum of health care costs required for producing an additional QALY. Because λ is usually an unknown quantity, we will use a range of plausible WTP levels. In our analysis we will use €20K, €50K and €80K for gaining one QALY (Zwaap, 2015). Within the regression framework, net-benefits are evaluated as a function of the intervention, patient characteristics and interaction terms (for effect modification) and thus helps to answer the question: who benefits most? – or more precisely, are there any subgroups in which the intervention is particularly cost-effective? This information may guide decisions about referring or not referring certain patients to the intervention such that referred patients may match better with the intervention and derive benefit from the intervention in a cost-effective way. We want to take the standard INBRA methodology one step further by using NBs as the dependent variable not only in a regression framework, but in a prognostic modelling framework (i.e. bootstrap aggregated CART analysis, random forest modelling, and machine learning) for identifying patient profiles where dose reduction is particularly cost-effective. This helps to select those patients where the health-economic benefits are likely to be most pronounced. Finally, we will also employ NBA to assess the impact of treatment characteristics (such as early application of cognitive behavioural therapy, adjunctive individual placement and support, or specific choices of antipsychotic medication) on net-benefits. The information generated by these analyses may help to improve the effectiveness and cost-effectiveness of the intervention.

8.5 Prognostic modelling

We are particularly interested in highly favourable versus unfavourable combinations of patient and treatment characteristics (listed under Objective 3) that predict long-term recovery (main outcome), successful discontinuation and sustained gains in health-related quality of life. Robust statistical techniques will be used such as bootstrap aggregated CART analysis (classification and regression tree analysis) and random forest analyses alternatingly using randomly drawn subsets to calibrate and validate the models. Determination of the final models will be made under consideration of the type, ease of measurement, and potential for early recognition and intervention of the predictors, as well as maximizing the combination of sensitivity, specificity, negative predictive value, and positive predictive value. Analysis will be conducted in data mining packages such as R, Python, SPSS Modeller and Stata.

9. OPTIMISING IMPLEMENTATION DURING THE TRIAL

9.1 Implementation and implementation research

The term ‘implementation’ requires clarification. We distinguish the (clinical) *intervention* (the C/D protocol) which has to be implemented, the *implementation strategy* which is the way the intervention is being implemented usually on the basis of an *implementation plan* detailing the responsibilities of actors, the required actions, the relevant time-paths, logistics, communications and other tools that are directed at achieving the desired *implementation goals*. Enhancing the implementation of an intervention in a clinical trial contributes to treatment integrity in the first place, but also offers an important opportunity to explore implementation issues. It is worth noting that *implementation research* monitors the execution of the implementation plan and identifies (contextual) factors that facilitate or impede achievement of the implementation goals. Implementation research is about evaluating the impacts of the implementation strategy; not primarily about the evaluation of the effectiveness of the intervention. The evaluation of the intervention indicates to what degree the intervention is (cost-)effective, whereas implementation research sheds light on (1) the impact of implementation strategies on the uptake of interventions in routine healthcare practice and (2) the characteristics of the implementation strategies that were used and (3) determinants (barriers and facilitators) of uptake such as (a) the *acceptability* of the intervention (the degree by which patients find the intervention desirable, acceptable and do comply with the intervention), (b) the *appropriateness* of the intervention (the degree by which the clinicians find the intervention relevant, practical and safe for their patients and can adhere to the D/D protocol), and ultimately (4) the *sustainability* of the intervention (the degree by the management of health services perceive the successful integration in existing health services and medical routines given a wide range of contextual factors (psychological, financial, organisational, legal, etc.). One of the main deliverables of implementation research is a newly developed and adapted implementation plan (elaborated as an implementation guide) to consolidate the integration of the new intervention in clinical practice and for scaling up.
In conclusion, once the evaluation of the intervention has demonstrated the effectiveness and cost-effectiveness, implementation research has helped to pave the way for integrating the intervention in existing clinical routines with the aim to sustain the intervention beyond the trial period and for scaling up.

9.2 Tasks and responsibilities

There are three activities that are crucial for implementation in the context of the proposed trial: (1) implementation of the intervention, (2) the evaluation of the implementation, (3) the development of an adapted implementation plan for future use. As member of the General Assembly, Prof. Wensing will be overseeing all implementation activities during the trial.

1. *Implementation of the intervention* will be led by Harm Gijsman (Pro Persona). They will be drawing up the implementation plan, securing engagement of all parties involved and are responsible for the execution of the implementation plan within the context of the trial.
2. *Evaluation of the implementation* will be carried out by the Implementation Centre of Trimbos Institute under coordination of Danielle van Duin (Trimbos Institute and Phrenos) to critically evaluate the degree by which the implementation goals are being achieved. To that end she will set up a monitoring system with indicators of critical factors and (intermediate) outcomes. This will feed into relevant focus groups of patients, clinicians and management, and presented to the General Assembly for review and ‘on the road’ decision-making regarding the implementation strategy.
3. Finally, further *development and adaptation of the implementation plan* will be the joint responsibility of all people and focus groups that were involved in the implementation. They will write a new and adapted implementation plan/guide for future use based on the experiences and the evaluation of the current one, with a specific focus on (amenable) factors that facilitate or impede the implementation of the C/D intervention.

9.3 Measurements, measures and analyses implementation research

The execution of the implementation plan will be monitored closely during the trial to draw lessons about facilitating and inhibiting factors as perceived by patients, clinicians and management at the participating centres. This is done with the aim to learn lessons as to pave the way for future scale-up of the C/D intervention once proven effective and cost-effective. To that end the following aspects of the intervention's implementation will be monitored for feasibility during the trial: (1) the impact of implementation on quality of care, (2) the characteristics of implementation strategies, (3) determinants (barriers and facilitators) of uptake and (4) the sustainability of the intervention.
For measures at different measurement points: see table 2.

1) The impact of implementation on quality of care

The impact of implementation efforts on quality of care will be monitored by measuring clinicians' adherence to (different aspects of) the discontinuation protocol (e.g. clear instruction, shared decision making, duration and timing of dose-reduction schedules, use of tapering strips, monitoring). All major deviations from the C/D protocol will be listed and used as input for systematic review regarding the feasibility, acceptability, appropriateness and sustainability of the original C/D protocol. For this purpose, professionals are asked to complete a questionnaire consisting of a fidelity scale and other quality indicators. Descriptive quantitative analyses will be performed on the self-report scores. Based on the results of this questionnaire semi-structured interviews will be conducted with professionals and clients for validation and interpretation of these results. Interviews with clients (around 5-10 questions) will be integrated in regular patient visits for the trial. All interviews will be recorded and analysed qualitatively.

2) Best practices and characteristics of implementation strategies

The content and appreciation of implementation processes and strategies will be monitored; How are tasks for implementation of the intervention planned and scheduled? To what degree and in what way is engaging of relevant individuals carried out? Which specific implementation strategies were used (for different subgroups of clients?) and how are they evaluated? For this purpose the existing implementation plan(s) of the trial will be analysed. Based on this plan(s) a semi-structured group interview will be conducted, using a domain of the CFIR for monitoring the general implementation process and asking for best practices and obstacles for monitoring specific implementation strategies. All interviews will be recorded and analysed qualitatively.

3) Facilitators and barriers, including acceptability and appropriateness

Facilitators and barriers, including appropriateness and acceptability, will be monitored by using some constructs of the Consolidated Framework of Implementation Research (CFIR).

- *Appropriateness.* Have clinicians been adequately informed about the intervention and its relative merits and risks? Do the clinicians regard the protocol appropriate (fitting, relevant, practical and safe) for their patients? What are first experiences regarding aspects of daily use such as: duration and timing of dose-reduction schedules, use of tapering strips? Have they been trained and do they feel competent in administering the intervention? Were any adverse outcomes reported (psychotic relapse, hospital readmission, suicidal behaviors) prompting deviations from the protocol and suggesting a need to make amendments to the protocol or to increase its flexibility and safety? What are first experiences about the appropriateness of using some research methods (EMA and prognostic modelling) in clinical practice?
- *Acceptability.* Have patients been adequately informed about the intervention and its relative merits and risks? Do patients find the intervention acceptable? How is their involvement and interest in the intervention? To what degree are they compliant with the D/D protocol? Are there any patient groups that did not receive the intervention? Have adverse outcomes been reported and critically reviewed? Can the C/D protocol be

tailored to individual needs and preferences to increase its acceptability and to improve compliance?

- *Other facilitators and barriers.* Other facilitating and inhibiting factors for implementing (different aspects of) the intervention and clinical use of research methods (e.g. EMA and prognostic modelling) will be monitored, such as: implementation climate in participating centres, external incentives, and self-efficacy and individual stage of change of professionals.

For this purpose professionals are asked to complete a questionnaire consisting of domains of the Consolidated Framework of Implementation Research (CFIR; Damschröder et al., 2009). Based on the results of this questionnaire semi-structured interviews will be conducted for validation and interpretation of these results. The interviews will be recorded and analysed qualitatively. For measuring acceptability by clients, a short interview (around 5-10 questions) will be integrated in regular patient visits for the trial.

4) The sustainability of the intervention

- *Sustainability.* Information of 4-year follow-up data on protocol adherence (fidelity) will be combined with experiences according to the management of the participating centres: can the C/D protocol be successfully integrated in the context of existing clinical routines and workflows? To what extend is the intervention offered to the intended target group? Were low SES segments in the patient population under-served? Other equity issues? What were the factors that furthered or impeded the intervention’s integration within clinical settings and routines? Are there any financial, regulatory and managerial obstacles to its continuation? Does management at the participating treatment centres intend to continue the intervention after the trial?

For this purpose professionals are asked to complete the questionnaire on fidelity and other quality indicators, as at other moments. In addition a semi-structured group interview will be conducted with managers from participating centres to discuss their experiences on sustainability of (different aspects of) the intervention. The interviews will be recorded and analysed qualitatively.

Table 2: measures and measurements implementation research

**Study duration**	**Patient visit**	**Professionals + management (in 26 centers)**<br><br>***(continuous tally number of patients discontinuing medication within complete case load)***		**Patients participating in the trial**	
Baseline		*fidelity measure (aspects of) intervention	*questionnaire (quality indicators and fidelity scale)		
	Close-out<br>(6 months)			*fidelity measure (aspects of) intervention<br>*acceptability (aspects of) intervention	*interview (fidelity scale and CFIR)
2 years<br><br>(*deliverable 1* available after this measure: *feedback to* participating sites)	Follow-up 2<br>(24 months)	*fidelity measure (aspects of) intervention<br>*implementation process/strategy<br>*appropriateness (aspects of) intervention + EMA + prognostic modelling + other inhibiting/ facilitating factors	*analyzing implementation plan(s) for study<br>*questionnaire (quality indicators, fidelity scale and CFIR)<br>*semi-structured group interview based on questionnaire	*fidelity measure (aspects of) intervention<br>*acceptability (aspects of) intervention	*interview (fidelity scale and CFIR)
4 years<br><br>(*deliverable 2:* after this measure: *short written report*)	Follow-up 4<br>(48 months)	*fidelity measure (aspects of) intervention	*questionnaire (quality indicators and fidelity scale)<br>*semi-structured group interview based on questionnaire	*fidelity measure (aspects of) intervention<br>*acceptability (aspects of) intervention	*interview (fidelity scale and CFIR)
6 years<br><br>*(deliverable 3* after this measure (6-7 year): *adaption of implementation plan)*	Follow-up 6<br>(72 months)	*fidelity measure (aspects of) intervention<br>*parts of CFIR evaluated by managers *(sustainability)*	*questionnaire (quality indicators and fidelity scale)<br>*semi-structured group interview based on questionnaire	*fidelity measure Cohort study<br>*acceptability Cohort study	*interview (fidelity scale and CFIR)
8 years<br><br>*(deliverable 4* after this measure *Relapse-*	Follow-up 8<br>(96 months)	*fidelity measure (cohort study)<br>*implementation process/strategy cohort study<br>*appropriateness	*analyzing implementation plan(s) for cohort study<br>*questionnaire (quality		

risk calculators made available)		(aspects of) cohort study + prognostic modelling + other inhibiting/ facilitating factors	indicators and fidelity scale)<br>*semi-structured group interview based on questionnaire		
10 years<br><br>(deliverable 5 after this measure: *initial prognostic models)*	Follow-up 10 (120 months)	*fidelity measure (cohort study)<br>*implementation adaptations process/strategy cohort study<br>*appropriateness (aspects of) cohort study + prognostic modelling + other inhibiting/ facilitating factors	*analyzing adapted implementation plan(s) for cohort study<br>*questionnaire (quality indicators and fidelity scale)<br>*semi-structured group interview based on questionnaire	*fidelity measure Cohort study<br>*acceptability Cohort study	*interview (fidelity scale and CFIR)
12 years<br><br>(deliverable 6 after this measure: *the final prognostic models)*		*fidelity measure (aspects of) cohort study<br>*parts of CFIR evaluated by managers *(sustainability)*	*questionnaire (quality indicators and fidelity scale)<br>*semi-structured group interview based on questionnaire	*fidelity measure Cohort study<br>*acceptability Cohort study	*interview (fidelity scale and CFIR)

9.4 Deliverables implementation research

The implementation research will provide the following deliverables:

- *After 2 years:* Feedback will be provided to participating sites on protocol adherence (fidelity measure), content and appreciation of implementation strategies, applicability and appropriateness of (aspects of) the intervention/research methods and other facilitators and barriers. Best practices will be shared among participating centers and implementation experts will provide advice concerning tailored selection of implementation strategies matching existing barriers and facilitators.
- *After 4 years:* A short written report will be provided to participating centers to be aware of changes in protocol adherence/fidelity within its own center and in relation to other centers. When prognostic modelling analyses show clear advantages of specific factors for well-defined patients, we will start the implementation of this approach immediately, beginning with the centres of our own consortium. The first step in scientific dissemination is by means of journal articles and congress presentations.
- *After 6 years:* Adaptation of implementation plan, in the form of a practical implementation guide with tools as smart cards of aspects of the intervention, quality indicators, advices on tailored implementation strategies. Best practices will be made transparent for all relevant centers in Dutch mental health care, complemented with advice of implementation experts concerning tailored selection of implementation strategies, in order to improve sustainability and scaling up beyond the trial period.
- *After 8 years:* Relapse-risk calculators will be made available for use by patients and professionals. The first findings of long-term follow-up will become available. We now

need to re-balance and discuss with patients, family and society how to weight short and long-term goals in functional, social end existential recovery.

- *After 10 – 12 years*: We will make the final prognostic models and use different outcomes. These models will be translated into lay people language and shared as short interactive video's with FEP patients, family and their informal helpers. The full collection of data is now open for sharing with other groups from outside the Netherlands.

10. FEASIBILITY

The project is led by the Psychosis Consortium and the patient organization Anoiksis. The Psychosis Consortium is an existing collaboration between more than 60 clinicians and researchers specialized in the treatment of psychosis, the patient organization Anoiksis and the family organization Ypsilon. The consortium was started in 2012 and has performed over 20 studies together, including drug trials, studies investigating psychosocial interventions and trials using focal brain stimulation. The consortium was initiated to facilitate participation in clinical relevant research for both patients and clinicians, to apply for grants together and to implement research findings as fast as possible. The consortium meets twice a year to discuss ongoing and new studies and has several regional meetings to discuss treatment of individual patients and new research findings. All university centers and most large mental health centers in the Netherlands are associated and have a dedicated person (often a psychiatry trainer or professor) to attend meetings and coordinate trials

This study aims to include 512 patients in 3 years. To allow this, we will work with 26 teams covering the Netherlands. Larger centers such as Parnassia, GGZ Centraal, Arkin, Lentis and Altrecht have multiple teams specialized in psychosis and will therefore include at multiple sites. Each team treats between 50 and 200 patients with a first psychosis yearly. To achieve 512 inclusions in 3 year, each site will need to include a mean number of 6 patients annually. Given that inclusion criteria for this study are very broad and given the sparse exclusion criteria, this is feasible. We expect that approximately 1 in 10 eligible patients will indeed be included in the trial, thus centers that see 200 patients annually may be expected to include 20 patients, while those seeing 50 patients are expected to include 5. Each participating center will have "dedicated includers" which may increase the percentage of 10% eligible patients to be included.

If for some reason inclusion may fall below these numbers, we will activate "rescue centers" in Belgium and Germany to increase inclusion rates. These centers are already involved in other European trials (OPTiMiSE and EULAST) and are willing to participate in the current trial and can be added when deemed necessary. The independent interim analysis after 1 year will provide information whether or not to add these centers.

11. ETHICAL CONSIDERATIONS

11.1 Regulation statement

The study will be conducted in accordance with this protocol as well as the principles of the Declaration of Helsinki (64th WMA general assembly; October 2013), the ICH-GCP guidelines and other applicable laws and regulations.

11.2 Recruitment

22 different specialized Early Psychosis Units (4 from university centres and 18 from large mental health services, covering the Netherlands) will collaborate to include a total of 512 patients over a three years period. We expect each team to include a mean of 8 patients annually. Given that psychosis teams treat an annual number of 50-200 first episode patients and the majority of these patients reach symptomatic remission, less than 10% of potential participants need to participate in order to fulfil inclusion norms. Each participating centre has a PI (a member of the psychosis consortium and member of the general assembly of this study) to promote and implement the study. Each site also has a (part-time) dedicated includer (i.e. a team member, for instance a nurse) located in the centre with the task to facilitate inclusion to assist clinicians in selecting and inviting potential participants. To avoid selection bias, every patient with first episode psychosis that is referred to the treatment team will be noted in a list using initials and date of birth. The dedicated includer will follow-up on this list regularly, to see who fulfils including criteria (i.e. being 3-6 months in remission) and can be informed about potential participation in the study. Reasons for non-participation will be noted.

11.3 Benefits and risks assessment, group relatedness

The number of patient visits will be limited and mainly requires time investment for few physical examinations, questionnaires and cognitive testing sessions. Blood samples will be collected at several occasions by experienced researchers or lab technicians, so health risk attributable to this procedure is minimal as well.

Potential benefits of participation concern that routine care consists of less extensive monitoring of symptom change and functioning compared to the current trial, so all patients may benefit from the thorough examinations during study participation. In addition, the current study will provide clear information as to whether patients who have remitted from a first psychotic episode should continue to use antipsychotic medication for at least one year or whether long term recovery is better when they gradually reduce their medication. This information provides the basis for shared, well-informed medical decision-making for a large group of patients, could demonstrate improved functioning. This could prevent or shorten hospitalizations, with the associated high costs for society as well as the negative impact on the patients' lives.

In the face of the limited additional burden for the patient when participating in the current trial as compared to routine treatment, and the possible positive outcome for future treatment, offering participation to selected patients appears to be justified.

11.4 Compensation for injury

The sponsor/investigator has a liability insurance which is in accordance with article 7, subsection 6 of the WMO.

The sponsor (also) has an insurance for participants in accordance with the legal requirements in the Netherlands (Article 7 WMO and the Measure regarding Compulsory Insurance for Clinical Research in Humans of 23th June 2003). This insurance provides cover for damage to research subjects through injury or death caused by the study.

1. € 650.000,-- (i.e. four hundred and fifty thousand euro) for death or injury for each subject who participates in the Research;

2. € 5.000.000,-- (i.e. three million five hundred thousand euro) for death or injury for all subjects who participate in the Research;
3. € 7.500.000,-- (i.e. five million euro) for the total damage incurred by the organisation for all damage disclosed by scientific research for the Sponsor as 'verrichter' in the meaning of said Act in each year of insurance coverage.
The insurance applies to the damage that becomes apparent during the study or within 4 years after the end of the study.

11.5 Incentives

Participants will receive a monetary reward of 50 euros for each visit during the study (per June 1, 2023). When a patient refuses to participate in specific study procedures, this will be taken into account with regards to the monetary reward. If subjects wish to abort the study before completion, payment will be in proportion to the visits that have been completed. Any travel expenses are reimbursed. Each extra visit will also be rewarded with 50 euros.

12. ADMINISTRATIVE ASPECTS, MONITORING AND PUBLICATION

12.1 Handling and storage of data and documents

Privacy laws and regulations will be adhered to during the complete study. The collection and processing of participants' personal information will be limited to what is necessary to ensure the study's scientific practicability, the evaluation of efficacy, adherence, side effects and the investigational product's safety. Information collected about participants during this clinical investigation will be treated confidentially. The investigator or her co-workers will collect data and transfer it without recording the patient's name or date of birth. Instead data will be coded with a participant identification number.
The file with the key to the code will be managed by one person. The source documents will be kept in a locked file cabinet with limited access of the research personnel. In accordance with national laws and guidelines and the specifications of the ICH-GCP guidelines, the investigators are obligated to archive all documents pertaining to the study for the legally required time period.
The acquired data and examination results will be entered into an electronic case record form (eCRF) that is accessible via the internet. Investigators will receive personal user names and passwords for this purpose, and data will be encrypted for transfer. It will be agreed before the start of the study which documents serve as source documents for all data entered into the eCRF. More details on the handling and storage of collected data can be found in the separate Data Management Plan (K6 I, dd June 7 2017).

12.2 Monitoring and Quality Assurance

Associated investigators will be carefully selected and comprehensively informed and trained regarding Good Clinical Practice (GCP), all study procedures and the required examinations and documentation. The quality of data acquisition will be confirmed by regular monitoring visits as described in the Monitoring Plan (K6 G, dd June 7 2017). The monitor in the central study site (UMCG) is independent from the study team and is not involved in the inclusion of participants and the design and implementation of the study. Other study sites performing study visits will be monitored according to the Monitoring Plan by a UMCG HAMLETT study team member who is trained as a monitor. All monitoring activities will be in line with national laws and guidelines and the specifications of the ICH-GCP guidelines. Reports will be filed in the local Investigator Site Files (UMC Utrecht: in CC send to the PI of the study in Utrecht and head of the department prof.dr. F.Scheepers and to humanresearchbrain@umcutrecht.nl).
Study monitors will visit the study site at regular intervals to monitor the execution of the study. Monitors will have access to all documents that are needed to perform their task according to the above-mentioned guidelines. Monitors will check whether requirements to conduct the study are met and study procedures are followed correctly, and will check the

study site's documentation, the participants' source data, eCRF entries, and the correct maintenance of the Investigator Site File. Investigators will permit trial-related monitoring, audits, ERB reviews and regulatory inspections, providing direct access to source data and study documents.

12.3 Amendments

A 'substantial amendment' is defined as an amendment to the terms of the ERB application, or to the protocol or any other supporting documentation, that is likely to affect to a significant degree:

- the safety or physical or mental integrity of the subjects of the trial;
- the scientific value of the trial;
- the conduct or management of the trial; or
- the quality or safety of any intervention used in the trial.

All substantial amendments will be submitted for approval to the ERB and to the competent authority.
For non-substantial amendments, only a notification will be send to the accredited ERB, which will be recorded and filed by the sponsor.

12.4 Annual progress report

The sponsor/investigator will submit a summary of the progress of the trial to the accredited ERB once a year. Information will be provided on the date of inclusion of the first subject, numbers of subjects included and numbers of subjects that have completed the trial, serious adverse events/serious adverse reactions, other problems, and amendments.

12.5 End of study report

The sponsor will notify the accredited ERB and the competent authority of the end of the study within a period of 90 days. The end of the study is defined as the last patient's last visit.
In case the study is ended prematurely, the sponsor will notify the accredited ERB and the competent authority within 15 days, including the reasons for the premature termination.
Within one year after the end of the study, the investigator/sponsor will submit a final study report with the results of the study, including any publications/abstracts of the study, to the accredited ERB and the Competent Authority.

12.6 Public disclosure and publication policy

The results of the study will be submitted for publication in an international peer-reviewed journal adhering to applicable privacy laws and regulations. Publication strategy will be determined by the principal investigator. No treatment group information will be made available until after study completion.

13. REFERENCES

Alvarez-Jimenez, M., O'Donoghue, B., Thompson, A., et al. (2016). Beyond clinical remission in first episode psychosis: Thoughts on antipsychotic maintenance vs. guided discontinuation in the functional recovery era. *CNS Drugs, 30*(5), 357-368.

Andreasen, N. C., Flaum, M., & Arndt, S. (1992). The comprehensive assessment of symptoms and history (CASH): An instrument for assessing diagnosis and psychopathology. *Archives of General Psychiatry, 49*(8), 615-623.

Arnsten, A. F., Wang, M., & Paspalas, C. D. (2015). Dopamine's actions in primate prefrontal cortex: Challenges for treating cognitive disorders. *Pharmacological Reviews, 67*(3), 681-696.

Ashoorian, D. M., Davidson, R. M., Rock, D. J. T., Dragovic, M., & Clifford, R. M. (2015a). A clinical communication tool for the assessment of psychotropic medication side effects. *Psychiatry Research, 230*(2), 643-657.

Asselt, A. D. I. van., Mastrigt, G. A. P. G. van., Dirksen, C. D., Arntz, A., Severens, J. H., & Kessels, A. G. H. (2009). How to deal with cost differences at baseline. *Pharmacoeconomics, 27*(6), 519-528.

Barbui, C., Nosè, M., Bindman, J., et al. (2005). Sex differences in the subjective tolerability of antipsychotic drugs. *Journal of Clinical Psychopharmacology, 25*(6), 521-526.

Barnes, T. R. (1989). A rating scale for drug-induced akathisia. *The British Journal of Psychiatry, 154*(5), 672-676.

Bernstein, D. P., Stein, J. A., Newcomb, M. D., et al. (2003). Development and validation of a brief screening version of the childhood trauma questionnaire. *Child Abuse and Neglect, 27*(2), 169-190.

Berry, K., Barrowclough, C., & Wearden, A. (2008). Attachment theory: A framework for understanding symptoms and interpersonal relationships in psychosis. *Behaviour Research and Therapy*, *46*(12), 1275-82. doi:10.1016/j.brat.2008.08.009.

Bos, F. M., Schoevers, R. A., & Rot, M. aan het. (2015). Experience sampling and ecological momentary assessment studies in psychopharmacology: A systematic review. *European Neuropsychopharmacology, 25*(11), 1853-1864.

Bressan, R. A., & Pilowsky, L. S. (2000). Imaging the glutamatergic system in vivo-relevance to schizophrenia. *European Journal of Nuclear Medicine, 27*(11), 1723-1731.

Briggs, A., Claxton, K., & Sculpher, M. (2006). *Decision modelling for health economic evalutation.* Oxford: Oxford University Press.

Brugha, T., Bebbington, P., Tennant, C., & Hurry, J. (1985). The List of Threatening Experiences: a subset of 12 life event categories with considerable long-term contextual threat. *Psychological medicine*, *15*(1), 189–194.

Buchsbaum, B., Hickok, G., & Humpries, C. (2001). Role of left posterior superior temporal gyrus in phonological processing for speech perception and production. Cognitive Science, 25(5), 663–678.

Burke, R. E., Fletcher, N. A., Harding, A. E., et al. (1992). Letters to the editor: Idiopathic torsion dystonia. *Movement Disorders, 7*(4), 387-391.

Campbell, M. K., Piaggio, G., Elbourne, D. R., & Altman, D. G. (2012). CONSORT 2010 statement: Extension to cluster randomised trials. *British Medical Journal, 345*(e5661), 1-21.

Cannon-Spoor, H. E., Potkin, S. G., & Wyatt, R. J. (1982). Measurement of premorbid adjustment in chronic schizophrenia. *Schizophrenia Bulletin, 8*, 470-484.

Chopra, P., Herrman, H., & Kennedy, G. (2008). Comparison of disability and quality of life measures in patients with long-term psychotic disorders and patients with multiple sclerosis. *International Journal of Rehabilitation Research, 31*(2), 141-149.

Clayton, A.H, McGarvey, E.L. & Clavet, G.J. (1997). The Changes in sexual Functioning Questionnaire (CSFQ): development, Reliability and Validity. *Psychopharmacology Bulletin, 33(4),* 731-745.

Cohen, S., Kamarck, T., & Mermelstein, R. (1983). A global measure of perceived stress. *Journal of Health and Social Behavior*, *24*(4), 385–396.

Collip, D., Oorschot, M., Thewissen, V., Os, J. van, Bentall, R., & Myin-Germeys, I. (2011). Social world interactions: how company connects to paranoia. *Psychological Medicine, 41*(5), 911-921.

Drummond, M. F., Sculpher, M. J., Torrance, G. W., O'Brien, B. J., & Stoddart, G. L. (2005). *Methods for the economic evaluation of health care programmes* (3nd ed.). Oxford: Oxford University Press.

Early Psychosis Guidelines Writing Group (2010). *Australian Clinical Guidelines for Early Psychosis. A brief summary for practitioners* (2nd ed.). Orygen Youth Health, Melbourne.

Factor, S., Lang, A., & Weiner, W. (Ed.) (2005). *Drug Induced Movement Disorders* (2nd ed.). United Kingdom: John Wiley and Sons.

Gaebel, W., Riesbeck, M., Wölwer, W., et al. (2011). Relapse prevention in first-episode schizophrenia--maintenance vs intermittent drug treatment with prodrome-based early intervention: results of a randomized controlled trial within the German Research Network on Schizophrenia. *Journal of Clinical Psychiatry, 72*(2), 205-218.

Gerlach, J., Korsgaard, S., Clemmesen, P., et al. (1993). The St. Hans Rating Scale for extrapyramidal syndromes. *Acta Psychiatrica Scandinavica, 87*(4), 244-252.

Giffort, D., Schmook, A., Woody, C., Vollendorf, C., & Gervain, M. (1995). *Construction of a Scale to Measure Consumer Recovery.* Springfield, IL: Illinois Office of Mental Healt.

Grundy, S. M., Cleeman, J. I., Daniels, S. R., et al. (2005). Diagnosis and management of the metabolic syndrome: An American Heart Association/National Heart, Lung, and Blood Institute Scientific Statement. *Circulation, 112*(17), 2735-2752.

Haan, L. de, Bruggen, M. van, Lavalaye, J., Booij, J., Dingemans, P. M., & Linszen, D. (2003). Subjective experience and D2 receptor occupancy in patients with recent-onset schizophrenia treated with low-dose olanzapine or haloperidol: a randomized, double-blind study. *The American Journal of Psychiatry, 160*(2), 303-309.

Haan, L. de, Lavalaye, J., Linszen, D., Dingemans, P. M., & Booij, J. (2000). Subjective experience and striatal dopamine D(2) receptor occupancy in patients with schizophrenia stabilized by olanzapine or risperidone. *The American Journal of Psychiatry, 157*(6), 1019-1020.

Haan, L. de, Weisfelt, M., Dingemans, P. M., Linszen, D. H., & Wouters, L. (2002). Psychometric properties of the Subjective Well-Being Under Neuroleptics scale and the Subjective Deficit Syndrome Scale. *Psychopharmacology, 162*(1):24-28.

Hagen, JM, Sutterland AL, Koeter MW, Lutter R, Cohen D, de Haan L (2017). Advanced glycation end products in recent-onset psychosis indicate early onset of cardiovascular risk. Journal of Clinical Psychiatry, 78(9):1395-1401, 10.4088/JCP.16m10972

Hakkaart-van Roijen, L. et al. (2002). Trimbos/iMTA: Questionnaire costs associated with psychiatric illness (TiC-P). Rotterdam: iMTA.

Harten, P. N. van, & Kahn, R. S. (1999). Tardive dystonia. *Schizophrenia Bulletin, 25*(4), 741-748.

Harten, P. N. van, Matroos, G. E., Hoek, H. W., & Kahn, R. S. (1996). The prevalence of tardive dystonia, tardive dyskinesia, parkinsonism and akathisia The Curaçao extrapyramidal syndromes study: I. *Schizophrenia Research, 19*(2-3), 195-203.

Hoch, J. (2009). Net benefit regression. In M. Kattan (Ed.), Encyclopedia of medical decision-making (pp. 806-812). Thousand Oaks, CA: SAGE Publications, Inc.

Humeniuk, R., Henry-Edwards, S., Ali, R., Poznyak, V., & Monteiro, M. G. (2010). *The alcohol, smoking and substance involvement screening test (ASSIST): Manual for use in primary care.* Geneve: World Health Organisation.

Huserau, D., Drummond, M., Petrou, S., et al. (2013). Consolidated health economic evaluation reporting standards (CHEERS) statement. *BMC Medicine, 11*(80), 1-6.

Insel, C., Reinen, J., Weber, J., et al. (2014). Antipsychotic dose modulates behavioral and neural responses to feedback during reinforcement learning in schizophrenia. *Cognitive, Affective & Behavioral Neuroscience, 14*(1),189-201.

Jones, S. H., Thornicroft, G., Coffey, M., & Dunn, G. (1995). A brief mental health outcome scale-reliability and validy of the Global Assessment of Functioning (GAF). *The British Journal of Psychiatry, 166*(5), 654-659.

Karson, C., Duffy, R. A., Eramo, A., Nylander, A. G., & Offord, S. J. (2016). Long-term outcomes of antipsychotic treatment in patients with first-episode schizophrenia: A systematic review. *Neuropsychiatric Disease and Treatment, 6*(12), 57-67.

Kay, S. R., Fiszbein, A., & Opler, L. A. (1987). The positive and negative syndrome scale (PANSS) for schizophrenia. *Schizophrenia Bulletin, 13*(2), 261-276.

Keefe, R. S., Goldberg, T. E., Harvey, P. D., Gold, J. M., Poe, M. P., & Coughenour, L. (2004). The Brief Assessment of Cognition in Schizophrenia: reliability, sensitivity, and comparison with a standard neurocognitive battery. *Schizophrenia research, 68*(2-3), 283–297.

Lecomte, T., Corbière, M., & Laisné, F. (2006). Investigating self-esteem in individuals with schizophrenia: Relevance of the self-esteem rating scale-short form. *Psychiatry Research*, *143*(1), 99-108. doi:10.1016/j.psychres.2005.08.019

Leemput, I. A. van de., Wichers, M., Cramer, A. O., et al. (2013). Critical slowing down as early warning for the onset and termination of depression. *Proceedings of the National Academy of Science of the United States of America, 111*(1), 87-92.

Leucht, S., Tardy, M., Komossa, K., Heres, S., Kissling, W., Davis, J. M. (2012). Maintenance treatment with antipsychotic drugs for schizophrenia. *The Cochrane Database of Systematic Reviews, 16*(5), 1-230.

Lewis, S.J. & Heaton, K. W. (1997). Stool form scale as a useful guide to intestinal transit time. *Scandinavian Journal of Gastroenterology*, *32*(9), 920–924.

Lipsey, M. W., & Wilson, D. B. (1993). The efficacy of psychological, educational, and behavioral treatment. Confirmation from meta-analysis. *The American Psychologist, 48*(12), 1181-1209.

Longden, E., & Read, J. Assessing and reporting the adverse effects of antipsychotic medication: A systematic review of clinical studies, and prospective, retrospective, and cross-sectional research. *Clinical Neuropharmacology,* 2016;39(1), 29-39.

Manca, A., Hawkins, N., & Sculpher, M. J. (2005). Estimating mean QALYs in trial-based cost-effectiveness analysis: The importance of controlling for baseline utility. *Health Economics, 14*(5), 487-496.

Marshall, M., Lewis, S., Lockwood, A., Drake, R., Jones, P., & Croudace, T. (2005). Association between duration of untreated psychosis and outcome in cohorts of first-episode patients. *Archives of General Psychiatry, 62*, 975-983.

Mauskopf, J. A, Sullivian, S. D., Annemans, L., et al. (2007). Principles of good practice for budget impact analysis: Report of the ISPOR Task Force on good research practices—budget impact analysis. *Value in Health, 10*(5), 336-347.

Mizrahi, R., Rusjan, P., Agid, O., et al. (2007). Adverse subjective experience with antipsychotics and its relationship to striatal and extrastriatal D2 receptors: A PET study in schizophrenia. *The American Journal of Psychiatry, 164*(4), 630-637.

Trimbos instituut. (2012). Multidisciplinaire richtlijn schizofrenie 2012. Read on June 7 2017, https://assets-sites.trimbos.nl/docs/06b5fc38-cf0b-4e43-bac9-7aef4b67a9c5.pdf

Myin-Germeys, I., Oorschot, M., Collip, D., Lataster, J., Delespaul, P., & Os, J. van. (2009). Experience sampling research in psychopathology: Opening the black box of daily life. *Psychological Medicine, 39*(9), 1533-1547.

Naber, D. (1995). A self-rating to measure subjective effects of drugs. *International Clinical Psychopharmacology, 10*(3), 133-138.

National Institute for Clinical Excellence. Psychosis and Schizophrenia in Adults: Treatment and Management. National Clinical Guideline 178. London: National Institute for Clinical Excellence; 2014.

Nimwegen, L. J. van, Haan, L. de, Beveren, N. J. van, Helm, M. van der, Brink, W. van den, Linszen, D. (2008). Effect of olanzapine and risperidone on subjective well-being and craving for cannabis in patients with schizophrenia or related disorders: A double-blind randomized controlled trial. *Canadian Journal of Psychiatry, 53*(6), 400-405.

Os, J. van, Delespaul, P., Barge, D., & Bakker, R. P. (2014b). Testing an mHealth momentary assessment routine outcome monitoring application: A focus on restoration of daily life positive mood states. *PLoS One, 9*(12), e115254.

Os, J. van, Delespaul, P., Wigman, J., Myin-Germeys, I., & Wichers, M. (2013). Beyond DSM and ICD: Introducing "precision diagnosis" for psychiatry using momentary assessment technology. *World Psychiatry, 12*(2), 113-117.

Os, J. van, Lataster, T., Delespaul, P., Wichers, M., & Myin-Germeys, I. (2014a) Evidence that a psychopathology interactome has diagnostic value, predicting clinical needs: An experience sampling study. *PLoS One, 9*(1), e86652.

Ostlund, S. B., Kosheleff, A. R., & Maidment, N. T. (2012). Relative response cost determines the sensitivity of instrumental reward seeking to dopamine receptor blockade. *Neuropsychopharmacology, 37*(12), 2653-2660.

Perna, G., Riva, A., Defillo, A., Sangiorgio, E., Nobile, M., & Caldirola, D. (2020). Heart rate variability: can it serve as a marker of mental health resilience? *Journal of Affective Disorders*, *263*, 754–761.

Ritsher, J. B., Otilingam, P. G., & Grajales, M. (2003). Internalized stigma of mental illness: Psychometric properties of a new measure. *Psychiatry Research*, *121*(1), 31-49.

Roberts, M., Russell, L. B., Paltiel, A. D., Chambers, M., McEwan, P., Krahn, M. (2012).

Conceptualizing a model: A report of the ISPOR-SMDM modelling good research practices task force-2. *Value in Health, 15*, 804-811.

Schooler, N. R., & Kane, J. M. (1982). Research diagnoses for tardive dyskinesia. *Archives of General Psychiatry, 39*(4), 486-487.

Schulz, K. F., Altman, D. G., & Moher, D (2010). CONSORT 2010 statement: Updated guidelines for reporting parallel group randomised trials. *Trials, 11*(32), 1-8.

Sheehan, D. V., Lecrubier, Y., Sheehan, K. H., et al. (1998). The Mini-International Neuropsychiatric Interview (M.I.N.I.): The development and validation of a structured diagnostic psychiatric interview for DSM-IV and ICD-10. *The Journal of Clinical Psychiatry, 59*(20), 22-33.

Sinha, P., Vandana, V. P., Lewis, N. V., Jayaram, M., & Enderby, P. (2015). Evaluating the effect of risperidone on speech: A cross-sectional study. *Asian Journal of Psychiatry*, *15*, 51–5.

Sluik, D., Geelen, A., de Vries, J. H. M., Eussen, S. J. P. M., Brants, H. A. M., Meijboom, S., … Feskens, E. J. M. (2016). A national FFQ for the Netherlands (the FFQ-NL 1.0): validation of a comprehensive FFQ for adults. *The British Journal of Nutrition*, *116*(5), 913–923.

Smith, B. W., Dalen, J., Wiggins, K., Tooley, E., Christopher, P., & Bernard, J. (2008). The Brief Resilience Scale: Assessing the ability to bounce back. *International Journal of Behavioral Medicine*, *15*(3), 194–200.

Sonntag, M., König, H. H., & Konnopka, A. (2015). The responsiveness of the EQ-5D and time trade-off scores in schizophrenia, affective disorders, and alcohol addiction. *Health and Quality of Life Outcomes,13*(114), 1-9.

Stroop, J. R. (1935). Studies of interference in serial verbal reactions. *Journal of Experimental Psychology, 18*, 643-662.

Sullivan, S. D., Mauskopf, J. A., Augustovski, F., et al. (2014). Budget impact analysis—Principles of good practice: Report of the ISPOR 2012 budget impact analysis good practice II task force. *Value in Health, 17*, 5-14.

Svedlund, J., Sjödin, I., & Dotevall, G. (1988). GSRS—a clinical rating scale for gastrointestinal symptoms in patients with irritable bowel syndrome and peptic ulcer disease. *Digestive Diseases and Sciences*, *33*(2), 129–134.

Thielen F, Lokkerbol J, Ising H, van der Gaag M, Veling W, de Haan L, Evers S. (in prep.). A health-economic model for assessing the cost-effectiveness and budget impacts of interventions aimed at preventing and treating psychosis.

Thompson, A., Singh, S., & Birchwood, M. (2016). Views of early psychosis clinicians on discontinuation of antipsychotic medication following symptom remission in first episode psychosis. *Early Intervention in Psychiatry, 10*(4), 355-361.

Versteegh, M. M., Vermeulen, K. M., Evers, S. M. A. A., Wit, G. A. de, Prenger, R., & Stolk, E. A. (2016). Dutch tariff for the five-level version of the EQ-5D. *Value in Health, 19*(4), 343-352.

Voruganti, L., & Awad, A. G. (2004). Neuroleptic dysphoria: Towards a new synthesis. *Psychopharmacology, 171*(2), 121-132.

Voruganti, L., Slomka, P., Zabel, P., et al. (2001). Subjective effects of AMPT-induced dopamine depletion in schizophrenia: Correlation between dysphoric responses and striatal D_2 binding ratios on SPECT imaging. *Neuropsychopharmacology, 25*, 642-650.

Wichers, M., Groot, P. C. Psychosystems, ESM Group, & EWS Group. (2016). Critical Slowing Down as a Personalized Early Warning Signal for Depression. *Psychotherapy and Psychosomatics, 85*(2), 114-116.

Wildgruber, D., Ackermann, H., Klose, U., Kardatzki, B., & Grodd, W. (1996). Functional lateralization of speech production at primary motor cortex: a fMRI study. *Neuroreport*, *7*(15-17), 2791–5.

Wolters, H. A. (2004). *Desired and undesired effects of antipsychotic treatment from a patient' perspective: The psychometric evaluation of a self-rating instrument.* Groningen: s.n.

Wolters, H. A., Knegtering, H., Wiersma, D., Bosch, R. J. van den. (2003). The spectrum of subjective effects of antipsychotic medication. *Acta Neuropsychiatrica, 15*, 274-279.

Wunderink, L., Nieboer, R. M., Wiersma, D., Sytema, S., & Nienhuis, F. J. (2013). Recovery in remitted first-episode psychosis at 7 years of follow-up of an early dose reduction/discontinuation or maintenance treatment strategy: Long-term follow-up of a 2-year randomized clinical trial. *JAMA Psychiatry, 70*(9), 913-920.

Wunderink, L., Nienhuis, F. J., Sytema, S., Slooff, C. J., Knegtering, R., & Wiersma, D. (2007). Guided discontinuation versus maintenance treatment in remitted first-episode psychosis: Relapse rates and functional outcome. *The Journal of Clinical Psychiatry, 68*(5), 654-661.

Zimet, G. D., Dahlem, N. W., Zimet, S. G., & Farley, G. K. (1988). The multidimensional scale of perceived social support. *Journal of Personality Assessment, 52*, 30-41.

Zorginstituut Nederland. (2016). *Bijlage 1 Kostenhandleiding: Methodologie van kostenonderzoek en referentieprijzen voor economische evaluaties in de gezondheidszorg.* Diemen: Zorginstituut Nederland. Downloaded on June 7, 2017.

Zorginstituut Nederland. (2016). *Richtlijn voor het uitvoeren van economische evaluaties in de gezondheidszorg.* Diemen: Zorginstituut Nederland. Downloaded on June 7, 2017.

Zwaap, J., Knies, S., Meijden, C. van der, Staal, P., & Heiden, L. van der. (2015). *Kosteneffectiviteit in de praktijk.* Diemen: Zorginstituut Nederland. Downloaded on 7 June, 2017, from https://www.zorginstituutnederland.nl/ publicaties/rapport/2015/ 06/26/kosteneffectiviteit-in-de-praktijk.
