# Supplementary material for: Early Dose Reduction or Discontinuation vs Maintenance Antipsychotics After First Psychotic Episode Remission: A Randomized Clinical Trial
Source: JAMA Psychiatry. 2025 Oct 1;83(1):68–73. doi: 10.1001/jamapsychiatry.2025.2525 (PMC12489793; doi:10.1001/jamapsychiatry.2025.2525)
Supplement: Supplement 2. — eAppendix 1. Study Design and Randomization eAppendix 2. Definition of Cross-Overs and Tapering Procedure eAppendix 3. Calculation of Olanzapine Equivalents eAppendix 4. CONSORT Flow Diagram eAppendix 5. Baseline Medication Use eAppendix 6. Mean (SD) Scores on Outcome Measures per Condition and Timepoint eAppendix 7. Safety and Tolerability: Detailed Information on SAE, AE, Self-Harm, Violence, Police Contact and Neurological Side-Effects eAppendix 8. Per Protocol Analyses: Generalized Mixed Models eAppendix 9. Sex-at-Birth Subanalyses: Generalized Mixed Models eAppendix 10. Sensitivity Analysis Including Only Patients With Diagnosis of Schizophrenia and Schizo-Affective Disorder [file jamapsychiatry-e252525-s002.pdf]

Supplemental Online Content

Sommer IE, de Beer F, Gangadin S, et al; for the HAMLETT-OPHELIA Consortium. Early dose reduction or discontinuation of antipsychotic medication after first psychotic episode remission. *JAMA Psychiatry*. Published online October 1, 2025. doi:10.1001/jamapsychiatry.2025.2525

eAppendix 1. Study Design and Randomization

eAppendix 2. Definition of Cross-Overs and Tapering Procedure

eAppendix 3. Calculation of Olanzapine Equivalents

eAppendix 4. CONSORT Flow Diagram

eAppendix 5. Baseline Medication Use

eAppendix 6. Mean (SD) Scores on Outcome Measures per Condition and Timepoint

eAppendix 7. Safety and Tolerability: Detailed Information on SAE, AE, Self-Harm, Violence, Police Contact and Neurological Side-Effects

eAppendix 8. Per Protocol Analyses: Generalized Mixed Models

eAppendix 9. Sex-at-Birth Subanalyses: Generalized Mixed Models

eAppendix 10. Sensitivity Analysis Including Only Patients With Diagnosis of Schizophrenia and Schizo-Affective Disorder

This supplemental material has been provided by the authors to give readers additional information about their work.

© 2025 American Medical Association. All rights reserved, including those for text and data mining, AI training, and similar technologies.

eAppendix 1. Study design and randomization

Study design and participants

HAMLETT is a multicenter, single-blind randomized controlled trial. Raters were blinded, but patients, family members and clinicians knew timing and velocity of DR/D. We used a catchment area that spanned the Netherlands, in a collaboration of 26 specialized Dutch psychosis centers (in and out-patient facilities). The full study protocol can be found elsewhere (Begemann, 2020).
Eligible patients were screened and informed by clinicians. Included participants were aged 16-60 years; used antipsychotic medication; had achieved symptomatic remission for 3-6 months (e.g. sustained improvement of psychotic symptoms, any remaining psychotic symptoms did not interfere with daily functioning); had a DSM-5 diagnosis of FEP schizophrenia, schizoaffective disorder, schizophreniform disorder, brief psychotic disorder, or unspecified schizophrenia spectrum and other psychotic disorder.
Exclusion criteria were dangerous or harmful behavior during FEP or the need for coercive treatment. Eligibility criteria were assessed with the Comprehensive Assessment of Symptoms and History interview (CASH). Diagnoses were re-assessed using information of up to one year after randomization from the clinical file, the CASH interview, the Global Assessment of Functioning scale (GAF)20 at different time points, and when needed additional information from the treating clinician.

Participants were enrolled between September 2017 and March 2023. Present analyses used data collected between September 2017 and October 2024. All participants provided written informed consent.

Conditions

Participants were randomized 1:1 to maintenance (≤25% dose reduction for at least 6 months after inclusion, i.e. 12 months after remission) or DR/D (>25% dose reduction within 6 months after inclusion i.e. 12 months after remission). Recommended tapering schedules (appendix 3) based on gradual hyperbolic discontinuation were provided to the DR/D group. When patients and clinicians decided not to follow the randomized condition, patients remained in the study as “cross-overs”.

Data sharing

The study investigators control the research data, which is still being collected and stored via a computing system maintained by University Medical Center Groningen, the Netherlands. Meta-data can be shared upon motivated request. Individual data will not be shared. The study protocol and the statistical analysis plan have been published before.

Reference

Begemann MJH, Thompson IA, Veling W, et al. To continue or not to continue? Antipsychotic medication maintenance versus dose-reduction/discontinuation in first episode psychosis: HAMLETT, a pragmatic multicenter single-blind randomized controlled trial. Trials 2020; 21: 1–19.

© 2025 American Medical Association. All rights reserved, including those for text and data mining, AI training, and similar technologies.

Figure 1: Randomization procedure

Prior to randomization

All inclusion and exclusion criteria were thoroughly checked, and all baseline assessments were completed.

Randomization

Randomization was stratified per treatment center, with a 1:1 allocation ratio. Unblinded study team members (EvtH, MB, and SG) performed all steps of the randomization procedure, using a web-based application (https://www.aleaclinical.eu/). Study team members performing the assessments are blind to allocation. When blinding is broken, for example, because a patient communicates about his medication use or discontinuation, the assessor is replaced by another rater who is still blind.

Notification of the participant

After randomization, unblinded study team members contacted participants by phone within two weeks to inform them of their assigned group. During this call, if a participant expressed strong reluctance to follow the assigned treatment condition, they were noted as a 'cross-over'. This means that the participant, together with the treating phycisian, decided whether the dose would be continued or reduced, and to what extent. Participants randomized in the discontinuation condition were pointed to the HAMLETT tapering guide (available via www.HAMLETT.nl).

Notification of treating physicians

Treating physicians were informed of the randomized condition and asked to provide written confirmation that they had read and understood the treatment protocols. For the discontinuation group, physicians were instructed to begin tapering the medication within the **next four weeks**. The preference for a cross-ove was also communicated to the treating physician.

Clinical consultation

Discontinue

The participant was provided with a physical copy of the **HAMLETT Tapering Guide***. Key topics discussed included the benefits and risks of tapering,recommended tapering schedu les, and the importance of having a close support person involved. Together with the participant, potential warning signs were documented

Continue

Maintenance of the current dose untill 12 months after remission was discussed. In case of a preference for cross-over, the physician explored the underlying reasons and tried to motivate the participant to follow the randomized treatment condition.

Follow-up with treating physicians

Discontinue

Three weeks after randomization, physicians were asked to confirm whether tapering had commenced, or whether both participant and physician still intended on reducing the dose before the three-month follow-up (V3). In case of early-warning signs, the dose reduction was paused. When symptoms persevered, the dose was increased and the occurrence of psychotic relapse was reported to the study team. In case of subsiding early warning signs, dose reduction was cautiously resumed.

Continue

Two months after randomization, physicians were asked to confirm that the participants were still on the same dose relative to baseline, and that any tapering did not exceed 25% of the original dosage. Switching to a different medication is allowed, on the condition that the new medication's dosage is equivalent to the original. Physicians were also requested to pro-actively report future deviations from the protocol within the 12 months after remission. Exceeding the 25% dose-reduction cut-off was noted as a cross-over at V3.

Monitoring randomization adherence

At the three-month (V3) and six-month (V4) follow-up points, self-reports from participants were reviewed to ensure adherence to their randomized condition. If a participant was found to be non-adherent, the treating physician was asked for confirmation. If non-adherence was confirmed, the participant was noted as a cross-over at V3 and/or V4.

Confirming followed protocol (for per protcol analyses)

Based on pharmacy data from the Dutch Foundation for Pharmaceutical Statistics, the adhered treatment groups were confired. A 25% dose-reduction relative to the dose at baseline was taken as a cut-off value. These 'as actually treated' groups were used for the per protocol analyses.

© 2025 American Medical Association. All rights reserved, including those for text and data mining, AI training, and similar technologies.

eAppendix 2: Definition of cross-overs and tapering procedure

Patients are randomized (1:1) to one of two treatment arms:

1. Continuation: Continuation of antipsychotic medication until at least one year after remission. A maximum reduction of 25% relative to dose at baseline assessment, or switching to another antipsychotic at a similar dose, was allowed.
2. Dose Reduction/Discontinuation (DR/D): Gradual dose reduction of at least 25%. Participants could taper down to complete discontinuation, though this was not required. Tapering schedules were suggested to both the participant and clinician.

If a participant expressed strong reluctance to follow the assigned treatment condition upon first notification of the treatment allocation, they were noted as an 'initial cross-over'. This indicated that the participant, together with the treating physician, decided whether the dose would be continued or reduced, and to what extent.

Adherence to the assigned treatment conditions was tracked for all participants during the first phase of trial participation (6-month follow up), based on both self-reported medication use and pharmacy dispensation data. When information was insufficient to determine randomization-adherence, the treating physician was contacted for confirmation.

Crossovers were defined as non-adherence to the assigned treatment conditions. More specifically:

1. Cross-over while randomized to continuation: Reducing more than 25% of the dose at baseline assessment within the first 12 months after randomization.

Cross-over while randomized to DR/D: Reducing less than 25% of the dose at baseline assessment within the first 12 months after randomization.

HAMLETT tapering procedure

The HAMLETT tapering guide was provided to participants in the DR/D group by their treating physician during the first clinical consultation after randomization. The guide was provided in physical form, and also available online at *www.HAMLETT.nl*. Key topics discussed included the benefits and risks of tapering, recommended tapering schedules, and the importance of having a close support person involved. Together with the participant, potential warning signs were documented. The same procedure was provided to the maintenance group when tapering was considered at a later timepoint (i.e. after 12 months).

General information on tapering
The tapering guide restates the goal and study procedure of the HAMLETT study and provides information on the risks and benefits of tapering. The guide repeatedly stresses the importance of good and timely communication with the treating physician during tapering. Participants were also informed about what to expect during tapering, including the time needed to readjust. Withdrawal symptoms can occur while tapering, which makes it important to taper slowly. Additionally, as the protective effects of antipsychotics diminish during tapering, the risk of rebound or relapse increases. To help prepare participants, information was provided on early warning signs.

Early warning signs
The early warning signs as described by Orygen (Creek et al., 2015) were translated into Dutch. Participants could indicate whether the early warning sign was present before or during their first psychotic episode. Participants were encouraged to also include the perspective of a close support person who was in frequent contact with them during the prodromal phase. Based on this information, an early warning action plan was devised in collaboration with the participant, a close support person and the treating physician. In addition, the participant could also mark down in the guide whether a specific early warning sign occurred during tapering. It was strongly emphasized that participants

© 2025 American Medical Association. All rights reserved, including those for text and data mining, AI training, and similar technologies.

should contact their treating physicians if early warning signs occurred. The guide included a diary template for participants to record their daily dose and how they felt during the tapering period.

Tapering schedules
The tapering schedules started from the maximal recommended dose according to the Summary of Product Characteristics (SPCs) of the antipsychotics, to provide comprehensive information. In practice, remission was frequently reached on lower doses, and consequently, the tapering schedules lasted shorter than described on the next page. Following recommendations later published by Horowitz et al. (2021), we ensured that the final steps in the tapering schedules were as gradual as possible, considering the available dosages. See also Table S2.

Social support
After signing informed consent, HAMLETT participants were asked to designate a specific close support person the role of a 'tapering buddy'. This person served as a contact for both the study and clinical teams and was aware of when and how the participant would taper off medication. Participants received two copies of the tapering guide: one for themselves and one for their close support person. The guide also provided information for the tapering buddy on how to support the participant during tapering. For example, the guide facilitated monitoring of the early warning signs by the tapering buddy.

References:
Creek R, Fraser S, O'Donoghue B, Hughes F, Crlenjak C. A shared understanding: psychoeducation in early psychosis. Orygen, The National Centre of Excellence in Youth Mental Health, 2015.
Horowitz MA, Jauhar S, Natesan S, Murray RM, Taylor D. A Method for Tapering Antipsychotic Treatment That May Minimize the Risk of Relapse. Schizophr Bull. 2021 Jul 8;47(4):1116-1129. doi: 10.1093/schbul/sbab017.

© 2025 American Medical Association. All rights reserved, including those for text and data mining, AI training, and similar technologies.

Table 2: Tapering schedules provided as guidelines for gradual discontinuation per antipsychotic drug based on regular available doses.

	Max. dose SPC	Available doses	Start tapering	After 2w	After 4w	After 6 w	After 8w	After 10w	After 12w	After 14w	After 16w	After 18w	After 20w	After 22w	After 24w	After 26w	After 28w	After 30w
Risperidone	10	0.5, 1, 2, 3, 4,6	10	8	6	5	4	3	2	1.5	1	0.5	0.5	0.25	0.25	0.25**	0.25**	stop
Olanzapine	20	2.5, 5, 10, 15, 20	20	17.5	15	12.5	10	7.5	5	5	2.5	2.5	1.25	1.25	1.25**	1.25**	stop	stop
Quetiapine	800	25, 100, 200, 300	800	600	500	400	300	200	150	100	75	50	25	12.5	12.5	stop	stop	stop
Aripiprazole	20	5,10, 15	20	17.5	15	12.5	10	10	7.5	7.5	5	5	2.5	2.5	2.5**	2.5**	stop	stop
Haloperidol	20	1, 5, 10	16	12	10	8	6	4	3	2	1	1	0.5	0.5	0.5**	0.5**	stop	stop
Zuclopenthixol	40	2, 10, 25	40	32	28	24	20	16	12	8	6	4	2	1	1	1**	1**	stop
Sulpiride	800	400, 50*	800	600	500	450	400	350	300	250	200	150	100	50	50	50**	50***	stop
Paliperidone	12	3*,6*,9*	12	12**	9	9**	9**	6	6	6**	3	3	3**	3**	3***	stop	stop	stop
Pimozide	20	1, 4	20	16	12	10	8	6	4	3	2	1	1	0.5	0.5	0.5**	0.5**	stop
Lurasidone	148	18.5, 37, 74	148	111	92.5	92.5	74	74	55.5	55.5	37	37	18.5	18.5	9.25	9.25	9.25**	stop
Clozapine	900	12.5*, 25, 100, 200	900	700	500	400	350	300	250	200	150	100	50	25	25	12.5	12.5	stop
Amisulpride	800	50, 100, 200, 400	800	700	600	500	400	350	300	250	200	150	100	50	25	25	25**	stop
Brexpiprazole	4	4*,3*,2*,1*	4	3	2	2	1	1	1**	1**	1***	1***	1***	stop	stop	stop	stop	stop
Flupentixol	18	0.5, 1, 3, 5	18	15	12	9	6	4	3	1.5	1	1	0.5	0.5	0.25	0.25	0.25**	stop
Cariprazine	6	1.5*, 3*, 4.5*, 6*	6	4.5	4.5	3	3	1.5	1.5	1.5**	1.5**	1.5***	1.5***	1.5***	stop	stop	stop	stop

w = weeks

* Undividable

** 1 dose on alternate days

*** 1 dose every 4 days

© 2025 American Medical Association. All rights reserved, including those for text and data mining, AI training, and similar technologies.

eAppendix 3: Calculation of olanzapine equivalents

Medication use was evaluated by means of a self-report questionnaire, and detailed data on medication dispense were provided by the Foundation for Pharmaceutical Statistics, from which olanzapine equivalents were calculated following the methods of Leucht et al. 2021.

The maximum effective dose method (Leucht et al., 2020) was used to calculate the equivalents of amisulpride, aripiprazole, brexpiprazole, cariprazine, haloperidol, lurasidone, olanzapine, paliperidone, quetiapine and risperidone. Such equivalents were not available for all other antipsychotics. Therefore, we used the minimum effective dose method (Leucht et al. 2014) for clozapine, and the Defined Daily Dose method (Leucht et al., 2016) for flupenthixol, penfluridol, pimozide, sulpiride, and zuclopenthixol.

References:

1. Leucht, S., Bauer, S., Siafis, S., et al. Examination of dosing of antipsychotic drugs for relapse prevention in patients with stable schizophrenia: a meta-analysis. JAMA psychiatry, 2021, 78(11), 1238-1248.
2. Leucht S, Crippa A, Siafis S et al. Dose-response meta-analysis of antipsychotic drugs for acute schizophrenia. Am J Psychiatry 2020;177:342-53.
3. Leucht S, Samara M, Heres S et al. Dose equivalents for second-generation antipsychotics: the minimum effective dose method. Schizophr Bull 2014;40:314-26.
4. Leucht S, Samara M, Heres S et al. Dose equivalents for antipsychotic drugs: the DDD method. Schizophr Bull 2016;42(Suppl. 1):S90-4.

© 2025 American Medical Association. All rights reserved, including those for text and data mining, AI training, and similar technologies.

eAppendix 4. CONSORT flow diagram

Figure 4: CONSORT diagram of participant flow throughout the trial

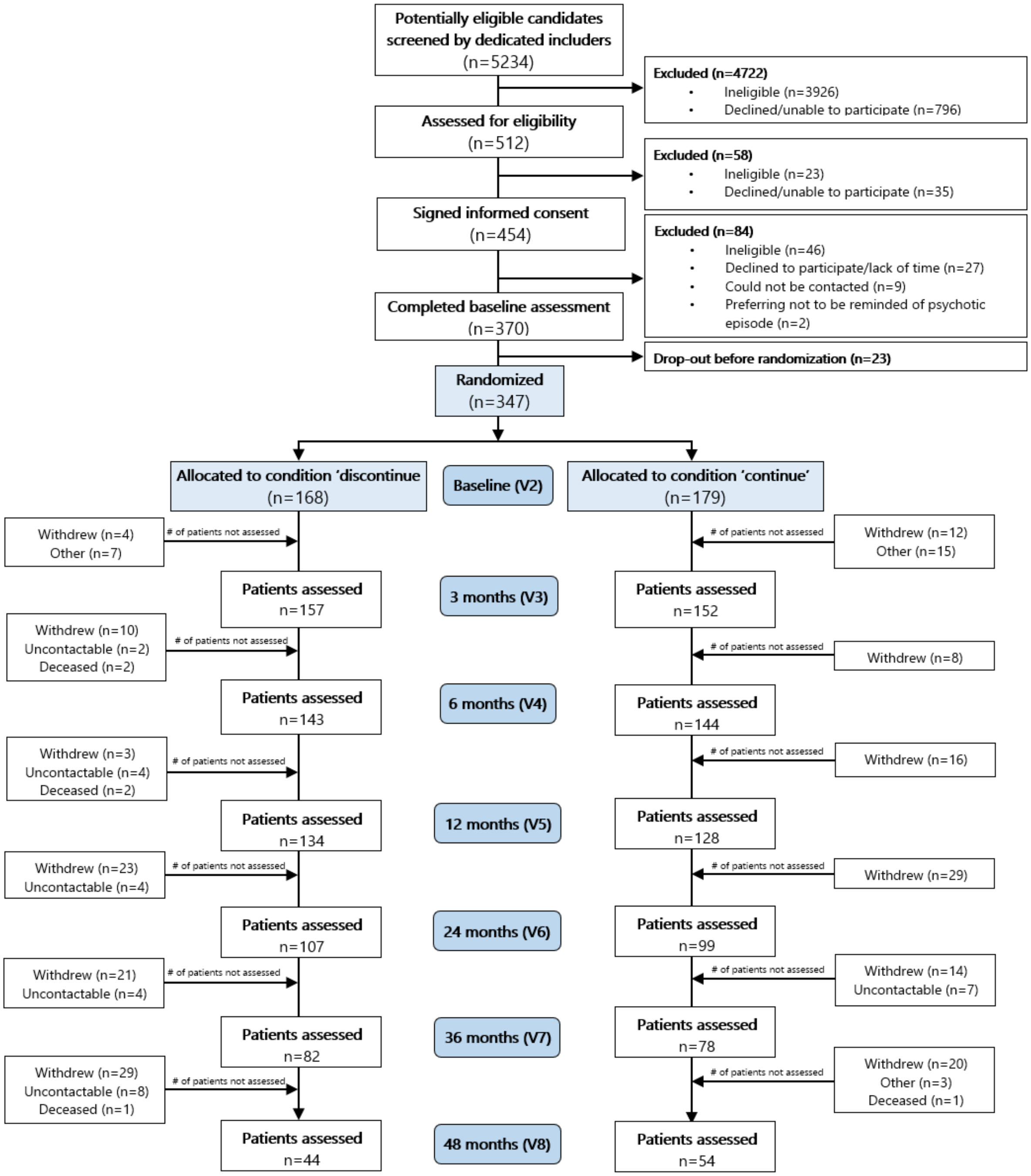

Figure 4. legend: Patients were kept in follow-up if they missed a visit. Numbers are therefore not cumulative, as continued efforts were made to follow up participants who did not participate in earlier follow-up assessments.

© 2025 American Medical Association. All rights reserved, including those for text and data mining, AI training, and similar technologies.

eAppendix 5. Detailed information on baseline medication use

Table 5: Baseline types and doses of antipsychotic medication per condition

	DR/D			Maintenance		
**AP medication**	**N**	**Mean**	**SD**	**N**	**Mean**	**SD**
AMISULPRIDE	2	125.00	35.36	5	390.00	174.64
ARIPIPRAZOLE	44	12.70	25.48	52	8.28	4.10
BREXPIPRAZOLE	0	0.00	0.00	2	2.50	0.71
CLOZAPINE	4	200.00	70.71	1	300.00	0.00
FLUPENTIXOL	2	6.00	5.66	3	2.17	1.61
HALOPERIDOL	13	5.46	13.41	20	4.05	5.21
LURASIDON	1	75.00	0.00	1	55.00	0.00
OLANZAPINE	71	11.95	24.20	66	8.70	4.96
PALIPERIDONE	3	4.75	2.17	2	4.00	1.41
QUETIAPINE	9	309.72	196.83	16	273.44	221.45
RISPERIDONE	18	2.17	1.06	17	2.09	0.97
SULPIRIDE	1	400.00	0.00	4	162.50	110.87
ZUCLOPENTIXOL	1	200.00	0.00	1	16.00	0.00

© 2025 American Medical Association. All rights reserved, including those for text and data mining, AI training, and similar technologies.

eAppendix 6: **Mean (SD) outcome measures per condition and timepoint linear mixed effects models for primary and secondary outcomes**

Table 6: mean (SD) scores on outcome measures per condition and timepoint linear mixed effects models for the primary and secondary outcomes

	Baseline		3 months		6 months		12 months		24 months		36 months		48 months	
	M n=179	DR/D n=168	M n=152	DR/D n=157	M n=144	DR/D n=143	M n=128	DR/D n=134	M n=99	DR/D n=108	M n=79	DR/D n=86	M n=54	DR/D n=47
**WHODAS-2**	52.3 (15.4)	57.4 (18.8)	49.7 (14.65)	54.4 (17.9)	48.7 (13.9)	52.1 (17.2)	48.9 (13.9)	52.8 (17.2)	48.6 (11.7)	52.4 (19.5)	46.8 (10.2)	50.8 (16.8)	50.4 (15.4)	54.9 (20)
**GAF**	65.5 (11.5)	64.2 (12.4)	67.9 (12.2)	68 (14.4)	69.7 (13)	68.5 (15.5)	70.4 (13.4)	68 (15.6)	69.6 (14)	69.7 (15.5)	69.8 (13.2)	71.6 (15.2)	67.9 (16.7)	72.7 (15.2)
**PANSS**	44 (9.7)	45.5 (10.3)	42.3 (9.5)	44.2 (12)	41.9 (9.5)	44 (12.6)	41.7 (10.2)	44.4 (12.6)	41.2 (9.1)	42.4 (11.4)	41.7 (10.6)	41.7 (11.2)	43.1 (12.7)	42 (12.5)
**EQ-5D-VAS**	71.3 (13.3)	69.2 (15.2)	74 (13.6)	73.5 (15.1)	77.3 (11.6)	72.4 (15.6)	76.3 (10.9)	72.3 (15.4)	78.2 (12.4)	72.9 (15.8)	77.6 (11.4)	74.8 (15.6)	79.1 (10)	76.2 (12.6)
**Prop. relapsed**	0 (0)	0 (0)	0.1 (0.3)	0.1 (0.3)	0.04 (0.2)	0.1 (0.3)	0.1 (0.3)	0.2 (0.4)	0.2 (0.4)	0.1 (0.3)	0.1 (0.3)	0.02 (0.2)	0.1 (0.3)	0 (0)
**Olanzapine Equivalents**	9.1 (5.5)	9.3 (5.8)	8 (5.6)	5.5 (6.7)	7 (6.1)	4.2 (7.5)	4.9 (5.4)	5 (6.9)	4.3 (5.3)	5.1 (6.1)	4.3 (5.2)	5.2 (8.1)	4.4 (5.2)	4.6 (8.3)
**BMI**	25 (4.1)	25.2 (4.6)	24.9 (4)	25.2 (4.2)	25.2 (4.6)	25.5 (4.7)	24.8 (4.4)	25 (4.5)	25 (4.1)	25.8 (4.8)	24.9 (3.8)	26.2 (5.8)	24.4 (4.1)	25.7 (5.4)
**Nr. patients medication free (%)**	0	0	9%	20%	20%	49%	35%	43%	44%	42%	44%	48%	43%	55%

WHODAS: WHO disability scale, GAF: global assessment of functioning, PANSS: positive and negative symptom scale, EQ-5D-VAS: visual analogue of the quality-of-life scale, BMI: body mass index

© 2025 American Medical Association. All rights reserved, including those for text and data mining, AI training, and similar technologies.

eAppendix 7. Safety and tolerability: detailed information on SAE, AE, self-harm, violence police contact and neurological side-effects

Table 7A: rates of self-harm, violent behaviour and contact with the police

	Baseline		3 months		6 months		12 months		24 months		36 months		48 months	
	M N=179	DR/D N=168	M N=152	DR/D N=157	M N=144	DR/D N=143	M N=128	DR/D N=134	M N=99	DR/D N=108	M N=79	DR/D N=86	M N=54	DR/D N=47
**Harm**														
No	157 (87.7%)	154 (91.7%)	128 (84.2%)	135 (86.0%)	126 (87.5%)	121 (84.6%)	106 (82.8%)	115 (85.8%)	75 (75.8%)	91 (84.3%)	64 (81.0%)	73 (84.9%)	46 (85.2%)	41 (87.2%)
Yes	2 (1.1%)	3 (1.8%)	5 (3.3%)	4 (2.5%)	0 (0%)	2 (1.4%)	5 (3.9%)	8 (6.0%)	2 (2.0%)	5 (4.6%)	3 (3.8%)	0 (0%)	2 (3.7%)	1 (2.1%)
Missing	20 (11.2%)	11 (6.5%)	19 (12.5%)	18 (11.5%)	18 (12.5%)	20 (14.0%)	17 (13.3%)	11 (8.2%)	22 (22.2%)	12 (11.1%)	12 (15.2%)	13 (15.1%)	6 (11.1%)	5 (10.6%)
**Violence**														
No	158 (88.3%)	154 (91.7%)	130 (85.5%)	134 (85.4%)	124 (86.1%)	119 (83.2%)	112 (87.5%)	114 (85.1%)	73 (73.7%)	94 (87.0%)	63 (79.7%)	69 (80.2%)	46 (85.2%)	41 (87.2%)
Yes	1 (0.6%)	3 (1.8%)	1 (0.7%)	5 (3.2%)	2 (1.4%)	5 (3.5%)	0 (0%)	7 (5.2%)	5 (5.1%)	2 (1.9%)	4 (5.1%)	4 (4.7%)	2 (3.7%)	1 (2.1%)
Missing	20 (11.2%)	11 (6.5%)	21 (13.8%)	18 (11.5%)	18 (12.5%)	19 (13.3%)	16 (12.5%)	13 (9.7%)	21 (21.2%)	12 (11.1%)	12 (15.2%)	13 (15.1%)	6 (11.1%)	5 (10.6%)
**Police**														
No	151 (84.4%)	151 (89.9%)	125 (82.2%)	129 (82.2%)	122 (84.7%)	116 (81.1%)	103 (80.5%)	115 (85.8%)	67 (67.7%)	85 (78.7%)	64 (81.0%)	69 (80.2%)	45 (83.3%)	41 (87.2%)
Yes	7 (3.9%)	4 (2.4%)	6 (3.9%)	8 (5.1%)	3 (2.1%)	7 (4.9%)	4 (3.1%)	6 (4.5%)	8 (8.1%)	8 (7.4%)	2 (2.5%)	3 (3.5%)	3 (5.6%)	0 (0%)
Missing	21 (11.7%)	13 (7.7%)	21 (13.8%)	20 (12.7%)	19 (13.2%)	20 (14.0%)	21 (16.4%)	13 (9.7%)	24 (24.2%)	15 (13.9%)	13 (16.5%)	14 (16.3%)	6 (11.1%)	6 (12.8%)

M: Maintenance, DR/D: Dose reduction/Discontinuation

© 2025 American Medical Association. All rights reserved, including those for text and data mining, AI training, and similar technologies.

Table 7B: Patients with AE, number of AE events by category and treatment group

Treatment group	AE category	Patients with AE	Number of AEs
M	Fatigue or sleep problems	20	23
	Gastrointestinal complaints or nausea	6	6
	Headache or dizziness	10	10
	Itching or rash	4	4
	Other (related to psychosis)	97	137
DR/D	Fatigue or sleep problems	33	37
	Gastrointestinal complaints or nausea	7	9
	Headache or dizziness	7	7
	Itching or rash	4	4
	Other (related to psychosis)	104	175

AE: adverse events, M: Maintenance, DR/D: Dose reduction/Discontinuation

Table 7C: Patients with SAE, number of SAE events by category and treatment group

Treatment group	SAE category	Patients with SAE	Number of SAEs
M	Hospitalization-initial or prolonged	42	42
M	Other	1	1
M	Death	1	1
DR/D	Death	5	5
DR/D	Hospitalization-initial or prolonged	32	32
DR/D	Other	3	3

SAE: severe adverse event, M: Maintenance, DR/D: Dose reduction/Discontinuation

© 2025 American Medical Association. All rights reserved, including those for text and data mining, AI training, and similar technologies.

Tabel 7D: Barnes Akathisia Rating Scale (BARS) and St. Hans Rating Scale (SHRS) per condition and visit: number and %

	Baseline		6 months		12 months		24 months		36 months		48 months	
	M N=179	DR/D N=168	M N=179	DR/D N=168	M N=179	DR/D N=168	M N=179	DR/D N=168	M N=179	DR/D N=168	M N=179	DR/D N=168
**Prop. symptoms of Parkinsonism**												
**Mild**	35 (19.6%)	34 (20.2%)	18 (10.1%)	17 (10.1%)	18 (10.1%)	16 (9.5%)	15 (8.4%)	10 (6.0%)	9 (5.0%)	7 (4.2%)	4 (2.2%)	4 (2.4%)
**Mild / moderate**	7 (3.9%)	12 (7.1%)	5 (2.8%)	5 (3.0%)	4 (2.2%)	11 (6.5%)	5 (2.8%)	5 (3.0%)	1 (0.6%)	4 (2.4%)	3 (1.7%)	3 (1.8%)
**Moderate**	1 (0.6%)	3 (1.8%)	0 (0%)	0 (0%)	0 (0%)	1 (0.6%)	0 (0%)	1 (0.6%)	0 (0%)	0 (0%)	0 (0%)	0 (0%)
**Moderate / severe**	0 (0%)	1 (0.6%)	0 (0%)	1 (0.6%)	0 (0%)	0 (0%)	0 (0%)	0 (0%)	0 (0%)	0 (0%)	0 (0%)	0 (0%)
**No symptoms**	73 (40.8%)	59 (35.1%)	71 (39.7%)	76 (45.2%)	57 (31.8%)	52 (31.0%)	32 (17.9%)	43 (25.6%)	37 (20.7%)	26 (15.5%)	14 (7.8%)	12 (7.1%)
**Uncertain**	40 (22.3%)	35 (20.8%)	28 (15.6%)	22 (13.1%)	11 (6.1%)	11 (6.5%)	15 (8.4%)	7 (4.2%)	4 (2.2%)	10 (6.0%)	4 (2.2%)	3 (1.8%)
**Missing**	23 (12.8%)	24 (14.3%)	57 (31.8%)	47 (28.0%)	89 (49.7%)	77 (45.8%)	112 (62.6%)	102 (60.7%)	128 (71.5%)	121 (72.0%)	154 (86.0%)	146 (86.9%)
**Prop. symptoms of dyskinesia**												
**Mild**	16 (8.9%)	6 (3.6%)	6 (3.4%)	3 (1.8%)	8 (4.5%)	3 (1.8%)	2 (1.1%)	1 (0.6%)	1 (0.6%)	1 (0.6%)	1 (0.6%)	1 (0.6%)
**Mild / moderate**	2 (1.1%)	2 (1.2%)	0 (0%)	2 (1.2%)	0 (0%)	2 (1.2%)	1 (0.6%)	0 (0%)	0 (0%)	2 (1.2%)	1 (0.6%)	1 (0.6%)
**Moderate**	0 (0%)	2 (1.2%)	0 (0%)	0 (0%)	0 (0%)	0 (0%)	0 (0%)	0 (0%)	0 (0%)	0 (0%)	0 (0%)	0 (0%)
**Moderate / severe**	0 (0%)	1 (0.6%)	0 (0%)	0 (0%)	0 (0%)	0 (0%)	0 (0%)	0 (0%)	0 (0%)	0 (0%)	0 (0%)	0 (0%)
**No symptoms**	133 (74.3%)	126 (75.0%)	109 (60.9%)	107 (63.7%)	81 (45.3%)	84 (50.0%)	53 (29.6%)	63 (37.5%)	45 (25.1%)	42 (25.0%)	23 (12.8%)	20 (11.9%)
**Uncertain**	10 (5.6%)	8 (4.8%)	10 (5.6%)	6 (3.6%)	3 (1.7%)	4 (2.4%)	9 (5.0%)	2 (1.2%)	5 (2.8%)	2 (1.2%)	1 (0.6%)	0 (0%)
**Missing**	18 (10.1%)	23 (13.7%)	54 (30.2%)	50 (29.8%)	87 (48.6%)	75 (44.6%)	114 (63.7%)	102 (60.7%)	128 (71.5%)	121 (72.0%)	153 (85.5%)	146 (86.9%)
**Prop. symptoms of dystonia**												
**Mild**	7 (3.9%)	15 (8.9%)	8 (4.5%)	7 (4.2%)	5 (2.8%)	4 (2.4%)	3 (1.7%)	3 (1.8%)	1 (0.6%)	2 (1.2%)	2 (1.1%)	2 (1.2%)
**Mild / moderate**	7 (3.9%)	6 (3.6%)	2 (1.1%)	2 (1.2%)	0 (0%)	2 (1.2%)	1 (0.6%)	0 (0%)	0 (0%)	2 (1.2%)	0 (0%)	3 (1.8%)
**Moderate**	1 (0.6%)	3 (1.8%)	0 (0%)	0 (0%)	0 (0%)	0 (0%)	0 (0%)	1 (0.6%)	0 (0%)	0 (0%)	0 (0%)	0 (0%)
**Moderate / severe**	0 (0%)	1 (0.6%)	0 (0%)	0 (0%)	0 (0%)	0 (0%)	0 (0%)	0 (0%)	0 (0%)	0 (0%)	0 (0%)	0 (0%)
**No symptoms**	141 (78.8%)	119 (70.8%)	109 (60.9%)	102 (60.7%)	89 (49.7%)	83 (49.4%)	58 (32.4%)	59 (35.1%)	44 (24.6%)	41 (24.4%)	22 (12.3%)	16 (9.5%)
**Uncertain**	8 (4.5%)	8 (4.8%)	12 (6.7%)	12 (7.1%)	3 (1.7%)	8 (4.8%)	4 (2.2%)	4 (2.4%)	4 (2.2%)	1 (0.6%)	2 (1.1%)	1 (0.6%)
**Missing**	15 (8.4%)	16 (9.5%)	48 (26.8%)	45 (26.8%)	82 (45.8%)	71 (42.3%)	113 (63.1%)	101 (60.1%)	130 (72.6%)	122 (72.6%)	153 (85.5%)	146 (86.9%)
**Prop. symptoms of akathisia**												
**Mild**	7 (3.9%)	15 (8.9%)	8 (4.5%)	7 (4.2%)	5 (2.8%)	4 (2.4%)	3 (1.7%)	3 (1.8%)	1 (0.6%)	2 (1.2%)	2 (1.1%)	2 (1.2%)
**Mild / moderate**	7 (3.9%)	6 (3.6%)	2 (1.1%)	2 (1.2%)	0 (0%)	2 (1.2%)	1 (0.6%)	0 (0%)	0 (0%)	2 (1.2%)	0 (0%)	3 (1.8%)
**Moderate**	1 (0.6%)	3 (1.8%)	0 (0%)	0 (0%)	0 (0%)	0 (0%)	0 (0%)	1 (0.6%)	0 (0%)	0 (0%)	0 (0%)	0 (0%)
**Moderate / severe**	0 (0%)	1 (0.6%)	0 (0%)	0 (0%)	0 (0%)	0 (0%)	0 (0%)	0 (0%)	0 (0%)	0 (0%)	0 (0%)	0 (0%)

© 2025 American Medical Association. All rights reserved, including those for text and data mining, AI training, and similar technologies.

	Baseline		6 months		12 months		24 months		36 months		48 months	
	M N=179	DR/D N=168	M N=179	DR/D N=168	M N=179	DR/D N=168	M N=179	DR/D N=168	M N=179	DR/D N=168	M N=179	DR/D N=168
**No symptoms**	141 (78.8%)	119 (70.8%)	109 (60.9%)	102 (60.7%)	89 (49.7%)	83 (49.4%)	58 (32.4%)	59 (35.1%)	44 (24.6%)	41 (24.4%)	22 (12.3%)	16 (9.5%)
**Uncertain**	8 (4.5%)	8 (4.8%)	12 (6.7%)	12 (7.1%)	3 (1.7%)	8 (4.8%)	4 (2.2%)	4 (2.4%)	4 (2.2%)	1 (0.6%)	2 (1.1%)	1 (0.6%)
**Missing**	15 (8.4%)	16 (9.5%)	48 (26.8%)	45 (26.8%)	82 (45.8%)	71 (42.3%)	113 (63.1%)	101 (60.1%)	130 (72.6%)	122 (72.6%)	153 (85.5%)	146 (86.9%)

M: Maintenance, DR/D: Dose reduction/Discontinuation, prop: proportion

© 2025 American Medical Association. All rights reserved, including those for text and data mining, AI training, and similar technologies.

eAppendix 8. Per protocol analyses: generalized mixed models

Table 8A: Summary of the linear mixed effects models for the primary and secondary outcomes from the per-protocol analysis.

Outcome	3 months	6 months	12 months	24 months	36 months	48 months
WHODAS	β = -0.24<br>-3.14 to 2.67<br>p = 0.873	β = -2.32<br>-5.32 to 0.68<br>p = 0.13	β = 0.03<br>-3.07 to 3.13<br>p = 0.984	β = -2.78<br>-6.19 to 0.62<br>p = 0.109	β = -2.89<br>-6.61 to 0.84<br>p = 0.129	β = -2.18<br>-6.7 to 2.34<br>p = 0.344
PANSS	β = -0.99<br>-2.99 to 1.01<br>p = 0.332	**β = -2.74**<br>**-4.8 to -0.68**<br>**p = 0.009**	β = -0.81<br>-2.94 to 1.32<br>p = 0.457	**β = -2.55**<br>**-4.89 to -0.21**<br>**p = 0.033**	**β = -4.86**<br>**-7.42 to -2.3**<br>**p = <.0001**	**β = -5.7**<br>**-8.84 to -2.55**<br>**p = <.0001**
GAF	β = 1.16<br>-1.43 to 3.76<br>p = 0.38	**β = 3.68**<br>**1 to 6.37**<br>**p = 0.007**	β = 2.11<br>-0.65 to 4.88<br>p = 0.134	**β = 4.55**<br>**1.52 to 7.58**<br>**p = 0.003**	**β = 5.27**<br>**1.92 to 8.63**<br>**p = 0.002**	**β = 7.52**<br>**3.42 to 11.62**<br>**p = <.0001**
EQ-5D-VAS	β = 2.1<br>-0.92 to 5.13<br>p = 0.173	β = 1.9<br>-1.18 to 4.98<br>p = 0.227	β = 0.07<br>-3.28 to 3.43<br>p = 0.966	β = 1.27<br>-2.28 to 4.82<br>p = 0.483	β = 3.71<br>-0.57 to 7.99<br>p = 0.089	β = -0.35<br>-5.06 to 4.35<br>p = 0.883
Relapse risk	reference	OR = 1.18<br>0.4 to 3.39<br>p = 0.757	OR = 1.62<br>0.58 to 4.34<br>p = 0.342	OR = 1.11<br>0.41 to 2.92<br>p = 0.829	OR = 0.93<br>0.34 to 2.46<br>p = 0.889	OR = 0.82<br>0.3 to 2.18<br>p = 0.702
BMI	β = -0.2<br>-0.66 to 0.27<br>p = 0.409	β = -0.38<br>-0.86 to 0.09<br>p = 0.115	**β = -0.72**<br>**-1.21 to -0.23**<br>**p = 0.004**	**β = -0.76**<br>**-1.3 to -0.22**<br>**p = 0.006**	β = -0.14<br>-0.74 to 0.45<br>p = 0.637	β = -0.59<br>-1.28 to 0.09<br>p = 0.091
Olanzapine equivalent	β = -3.83<br>-4.89 to -2.77<br>p = <.0001	β = -6.28<br>-7.34 to -5.22<br>p = <.0001	β = -4.02<br>-5.08 to -2.96<br>p = <.0001	β = -3.28<br>-4.33 to -2.22<br>p = <.0001	β = -2.71<br>-3.81 to -1.69<br>p = <.0001	β = -2.83<br>-3.91 to -1.79<br>p = <.0001

WHODAS: WHO disability scale, GAF: global assessment of functioning, PANSS: positive and negative symptom scale, EQ-5D-VAS: visual analogue of the quality-of-life scale, BMI: body mass index

© 2025 American Medical Association. All rights reserved, including those for text and data mining, AI training, and similar technologies.

Figure 8B: mean differences and 95% confidence intervals in primary and secondary outcome measures from baseline for the per protocol treated groups

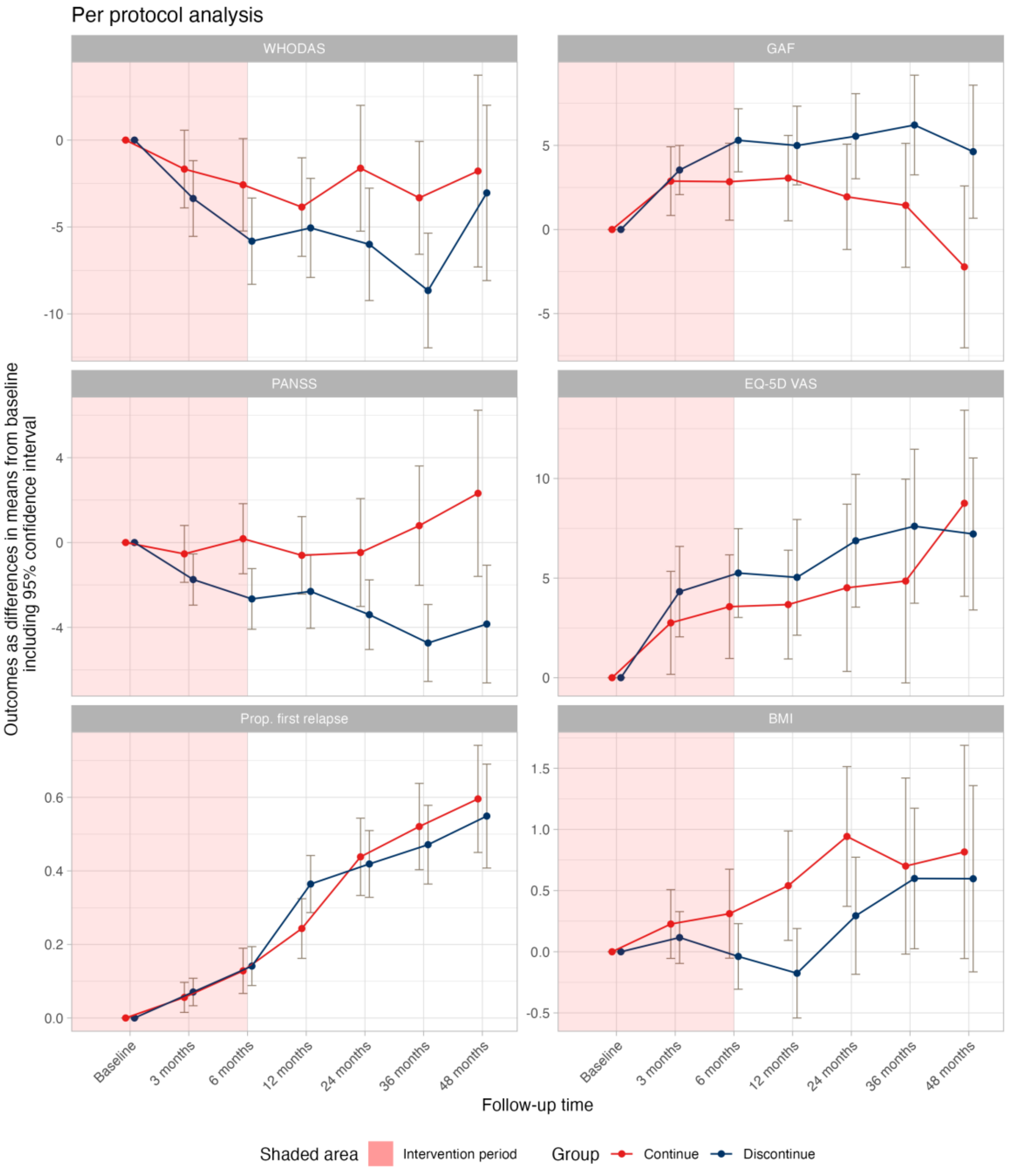

© 2025 American Medical Association. All rights reserved, including those for text and data mining, AI training, and similar technologies.

eAppendix 9. Sex-at-birth subanalyses: generalized mixed models

Table 9A: Summary of the linear mixed effects models for the primary and secondary outcomes <u>for male participants only</u>

Outcome	3 months	6 months	12 months	24 months	36 months	48 months
WHODAS	β =1.33<br>(-2.19 to 4.85)<br>p = 0.459	β =0.5<br>(-3.14 to 4.14)<br>p = 0.788	β =0.12<br>(-3.68 to 3.93)<br>p = 0.949	β =-0.53<br>(-4.71 to 3.65)<br>p = 0.804	β =-0.85<br>(-5.49 to 3.79)<br>p = 0.719	β =1.95<br>(-3.81 to 7.72)<br>p = 0.507
PANSS	β =1.44<br>(-0.99 to 3.86)<br>p = 0.246	β =1.76<br>(-0.75 to 4.28)<br>p = 0.169	β =1.45<br>(-1.18 to 4.07)<br>p = 0.281	β =-0.08<br>(-2.96 to 2.8)<br>p = 0.957	β =-1.53<br>(-4.73 to 1.67)<br>p = 0.349	β =-2.24<br>(-6.24 to 1.76)<br>p = 0.273
GAF	β =0.03<br>(-3.02 to 3.09)<br>p = 0.983	β =-2.66<br>(-5.83 to 0.52)<br>p = 0.101	β =-2.23<br>(-5.54 to 1.08)<br>p = 0.187	β =1.02<br>(-2.6 to 4.64)<br>p = 0.579	β =1.94<br>(-2.12 to 5.99)<br>p = 0.35	**β =5.56<br>(0.5 to 10.62)<br>p = 0.031**
EQ-5D-VAS	β =1.61<br>(-2 to 5.22)<br>p = 0.382	**β =-4.19<br>(-7.85 to -0.52)<br>p = 0.025**	**β =-4.55<br>(-8.65 to -0.44)<br>p = 0.03**	β =-2.36<br>(-6.71 to 2)<br>p = 0.288	β =2.12<br>(-3.2 to 7.44)<br>p = 0.434	β =-0.45<br>(-6.46 to 5.56)<br>p = 0.883
BMI	β =-0.09<br>(-0.63 to 0.46)<br>p = 0.757	β =-0.29<br>(-0.84 to 0.26)<br>p = 0.295	β =-0.23<br>(-0.81 to 0.35)<br>p = 0.428)	β =0.06<br>(-0.58 to 0.69)<br>p = 0.856	β =0.18<br>(-0.53 to 0.88)<br>p = 0.626	β =0.41<br>(-0.43 to 1.24)<br>p = 0.339
Olanzapine equivalent	**β =-2.67<br>(-3.93 to -1.41)<br>p <.0001**	**β =-2.82<br>(-4.08 to -1.56)<br>p <.0001**	β =-1.08<br>(-2.34 to 0.18)<br>p = 0.094	β =-0.48<br>(-1.74 to 0.78)<br>p = 0.458	β =-0.35<br>(-1.61 to 0.92)<br>p = 0.59	β =-0.36<br>(-1.63 to 0.9)<br>p = 0.574
Relapse risk	reference	OR = 1.63<br>(0.52 to 5.19)<br>p = 0.403	OR = 2.29<br>(0.77 to 6.9)<br>p = 0.135	OR = 2.2<br>(0.75 to 6.59)<br>p = 0.15	OR = 1.99<br>(0.67 to 5.98)<br>p = 0.214	OR = 1.88<br>(0.63 to 5.68)<br>p = 0.255

WHODAS: WHO disability scale, GAF: global assessment of functioning, PANSS: positive and negative symptom scale, EQ-5D-VAS: visual analogue of the quality-of-life scale, BMI: body mass index

© 2025 American Medical Association. All rights reserved, including those for text and data mining, AI training, and similar technologies.

Table 9: Summary of the linear mixed effects models for the primary and secondary outcomes for female participants only

Outcome	3 months	6 months	12 months	24 months	36 months	48 months
WHODAS	β =0.93 (-3.94 to 5.81) p = 0.707	β =-0.6 (-5.59 to 4.39) p = 0.814	β =4.03 (-1.07 to 9.14) p = 0.121	β =4.68 (-1 to 10.36) p = 0.106	β =2.86 (-3.27 to 8.98) p = 0.36	β =-3.22 (-10.45 to 4.01) p = 0.382
PANSS	β =-0.29 (-3.58 to 3) p = 0.864	β =-0.16 (-3.53 to 3.21) p = 0.927	β =2.97 (-0.48 to 6.42) p = 0.091	β =1.99 (-1.86 to 5.83) p = 0.311	β =-1.86 (-6.01 to 2.29) p = 0.38	β =-4 (-9.03 to 1.02) p = 0.118
GAF	β =2.24 (-2.34 to 6.82) p = 0.337	β =3.95 (-0.74 to 8.65) p = 0.099	β =-0.82 (-5.61 to 3.97) p = 0.736	β =0.78 (-4.57 to 6.13) p = 0.776	**β =7.02 (1.19 to 12.85) p = 0.018**	**β =7.37 (0.41 to 14.34) p = 0.038**
EQ-5D-VAS	β =-1.34 (-6.46 to 3.77) p = 0.606	β =-1.36 (-6.63 to 3.91) p = 0.613	β =0.12 (-5.46 to 5.69) p = 0.967	β =-4.84 (-10.74 to 1.07) p = 0.108	β =-5.63 (-12.7 to 1.45) p = 0.119	β =-2.24 (-9.73 to 5.25) p = 0.557
BMI	β =0.18 (-0.67 to 1.03) p = 0.672	β =0.25 (-0.65 to 1.14) p = 0.587	β =0.82 (-0.08 to 1.72) p = 0.075	β =0.96 (-0.05 to 1.97) p = 0.063	**β =1.22 (0.13 to 2.32) p = 0.029**	β =0.16 (-1.06 to 1.37) p = 0.802
Olanzapine equivalent	β =-1.92 (-3.91 to 0.07) p = 0.059	**β =-2.71 (-4.71 to -0.72) p = 0.008**	β =-0.32 (-2.31 to 1.67) p = 0.753	β =0.35 (-1.64 to 2.34) p = 0.732	β =-0.11 (-2.1 to 1.89) p = 0.917	β =-0.21 (-2.2 to 1.78) p = 0.837
Relapse risk	reference	OR = 3.15 (0.26 to 79.95) p = 0.394	OR = 7.5 (0.76 to 172.34) p = 0.111	OR = 5.65 (0.59 to 126.87) p = 0.166)	OR = 3.94 (0.41 to 88.43) p = 0.271	OR = 2.85 (0.3 to 64.22) p = 0.401

WHODAS: WHO disability scale, GAF: global assessment of functioning, PANSS: positive and negative symptom scale, EQ-5D-VAS: visual analogue of the quality-of-life scale, BMI: body mass index

© 2025 American Medical Association. All rights reserved, including those for text and data mining, AI training, and similar technologies.

Figure 9C: Mean differences and 95% confidence intervals in primary and secondary outcome measures from baseline by sex

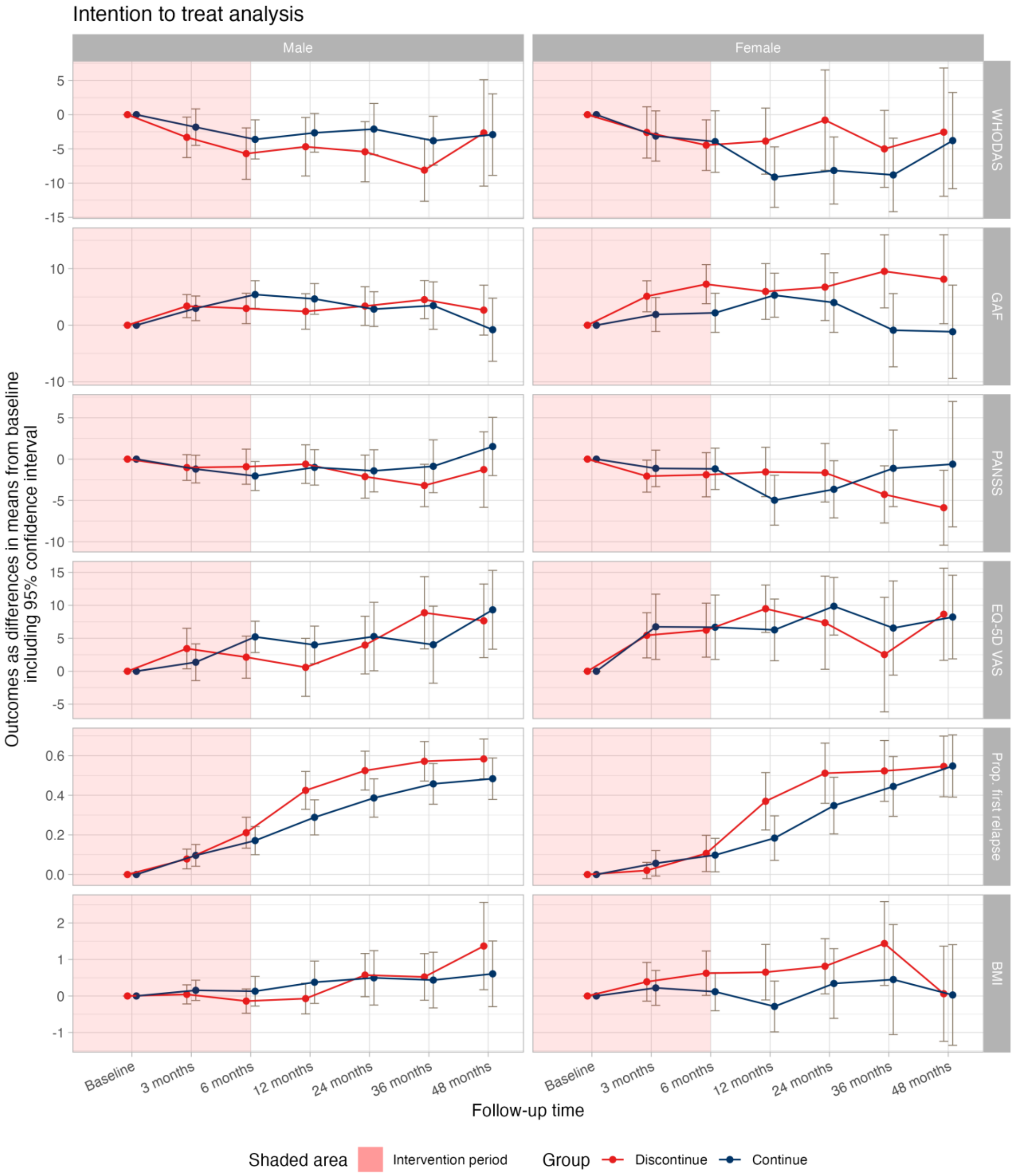

© 2025 American Medical Association. All rights reserved, including those for text and data mining, AI training, and similar technologies.

eAppendix 10. Sensitivity analysis including only participants with schizophrenia or schizo-affective disorder.

Table 10A: Mean scores on the outcome measures per condition and timepoint

	Baseline		3 months		6 months		12 months		24 months		36 months		48 months	
**Outcome**	**DR/D (n=116)**	**M (n=122)**	**DR/D (n=110)**	**M (n=107)**	**DR/D (n=100)**	**M (n=101)**	**DR/D (n=90)**	**M (n=90)**	**DR/D (n=70)**	**M (n=71)**	**DR/D (n=56)**	**M (n=58)**	**DR/D (n=31)**	**M (n=38)**
WHODAS	58.63 (19.6)	54.12 (16.95)	57.79 (19.2)	51.86 (16.18)	54.94 (18.65)	51.05 (14.96)	56.38 (18.74)	50.67 (15.18)	56.6 (21.9)	50.1 (12.83)	54.79 (18.56)	48 (10.84)	59.71 (21.04)	51.79 (16.77)
GAF	61.91 (12.5)	63.22 (10.92)	63.91 (14.05)	64.73 (11.3)	64.76 (15.08)	67.15 (12.84)	64.24 (14.7)	67.38 (12.7)	65.97 (14.44)	67.86 (13.67)	67 (15.95)	68.57 (12.96)	68.1 (15.45)	65.58 (16.57)
PANSS	47.54 (10.68)	45.57 (9.59)	47.25 (12.71)	43.91 (9.85)	46.42 (13.18)	43.5 (10.11)	46.98 (13.44)	43.39 (10.05)	45.07 (11.62)	42.24 (8.61)	43.98 (11.18)	43.42 (11.25)	45.48 (13.66)	46.05 (13.59)
EQ-5D-VAS	67.98 (15.99)	70.32 (13.8)	70.64 (16.15)	72.84 (13.38)	70 (16.32)	75.85 (11.88)	69.87 (13.46)	74.91 (11.45)	70.47 (15.24)	78.26 (12.71)	70.85 (16.77)	77.49 (11.96)	72.96 (12.03)	80.36 (10.5)
Prop. relapse	0 (0)	0 (0)	0.07 (0.26)	0.07 (0.26)	0.15 (0.36)	0.14 (0.35)	0.41 (0.49)	0.23 (0.43)	0.52 (0.5)	0.37 (0.49)	0.55 (0.5)	0.44 (0.5)	0.59 (0.5)	0.63 (0.49)
Ola Eq.	9.76 (6.01)	9.32 (5.68)	6.47 (7.43)	8.25 (5.54)	5.16 (8.07)	7.13 (6.35)	5.89 (7.57)	4.82 (5.5)	5.33 (5.82)	4.4 (5.36)	6.52 (9.14)	4.47 (5.27)	6.56 (9.62)	5.11 (5.42)
BMI	25.59 (4.71)	24.98 (4.32)	25.71 (4.57)	24.88 (4.25)	25.82 (4.65)	25.21 (4.78)	25.36 (4.6)	24.95 (4.41)	26.27 (5.16)	24.56 (3.94)	27.07 (6.21)	24.44 (3.41)	26.81 (5.99)	24.33 (4.06)

M: Maintenance, DR/D: Dose reduction/Discontinuation, WHODAS: WHO disability scale, GAF: global assessment of functioning, PANSS: positive and negative symptom scale, EQ-5D-VAS: visual analogue of the quality-of-life scale, BMI: body mass index

© 2025 American Medical Association. All rights reserved, including those for text and data mining, AI training, and similar technologies.

Table 10B: Summary of the linear mixed effects models for the primary and secondary outcomes from the ITT sensitivity analysis

**Outcome**	**3 months**	**6 months**	**12 months**	**24 months**	**36 months**	**48 months**
WHODAS	β =2.72<br>(-0.95 to 6.39)<br>p = 0.146	β =0.58<br>(-3.21 to 4.36)<br>p = 0.766	β =2.74<br>(-1.22 to 6.7)<br>p = 0.175	β =2.87<br>(-1.51 to 7.24)<br>p = 0.199	β =1.93<br>(-2.86 to 6.72)<br>p = 0.429	β =2.17<br>(-3.72 to 8.06)<br>p = 0.47
PANSS	β =2<br>(-0.43 to 4.44)<br>p = 0.107	β =1.89<br>(-0.63 to 4.41)<br>p = 0.141	β =2.02<br>(-0.62 to 4.65)<br>p = 0.133	β =0.71<br>(-2.2 to 3.62)<br>p = 0.632	β =-1.84<br>(-5.03 to 1.34)<br>p = 0.255	β =-3.41<br>(-7.38 to 0.55)<br>p = 0.092
GAF	β =-0.17<br>(-3.17 to 2.84)<br>p = 0.914	β =-1.65<br>(-4.77 to 1.47)<br>p = 0.3	β =-1.86<br>(-5.1 to 1.39)<br>p = 0.262	β =0.43<br>(-3.16 to 4.03)<br>p = 0.813	β =1.6<br>(-2.38 to 5.59)<br>p = 0.43	**β =5.62<br>(0.69 to 10.55)<br>p = 0.025**
EQ-5D-VAS	β =-0.44<br>(-4.09 to 3.21)<br>p = 0.811	β =-3.65<br>(-7.39 to 0.08)<br>p = 0.055	β =-2.67<br>(-6.79 to 1.46)<br>p = 0.205	β =-4.03<br>(-8.44 to 0.37)<br>p = 0.073	β =-2.7<br>(-8.13 to 2.72)<br>p = 0.328	β =-2.93<br>(-8.75 to 2.89)<br>p = 0.324
BMI	β =0.02<br>(-0.56 to 0.59)<br>p = 0.959	β =-0.1<br>(-0.68 to 0.49)<br>p = 0.749	β =0.28<br>(-0.34 to 0.89)<br>p = 0.376	**β =0.76<br>(0.07 to 1.44)<br>p = 0.03**	**β =0.98<br>(0.24 to 1.73)<br>p = 0.01**	β =0.35<br>(-0.51 to 1.21)<br>p = 0.422
Olanzapine equivalent	β =-1.83<br>(-3.18 to -0.48)<br>p = 0.008	β =-1.96<br>(-3.31 to -0.62)<br>p = 0.004	β =-0.12<br>(-1.47 to 1.23)<br>p = 0.86	β =-0.04<br>(-1.39 to 1.3)<br>p = 0.949	β =0.34<br>(-1.01 to 1.69)<br>p = 0.623	β =0.31<br>(-1.04 to 1.66)<br>p = 0.648
Relapse risk	Reference*	OR = 1.91<br>(0.6 to 6.27)<br>p = 0.276	OR = 3.24<br>(1.09 to 10.02)<br>p = 0.037	OR = 3.23<br>(1.1 to 9.86)<br>p = 0.035	OR = 2.85<br>(0.96 to 8.75)<br>p = 0.061	OR = 2.27<br>(0.77 to 6.99)<br>p = 0.143

WHODAS: WHO disability scale, GAF: global assessment of functioning, PANSS: positive and negative symptom scale, EQ-5D-VAS: visual analogue of the quality-of-life scale, BMI: body mass index

© 2025 American Medical Association. All rights reserved, including those for text and data mining, AI training, and similar technologies.

Figure 10C Mean differences and 95% confidence intervals of primary and secondary outcomes per timepoint for the subgroup with only diagnosis of schizophrenia and schizo-affective disorder

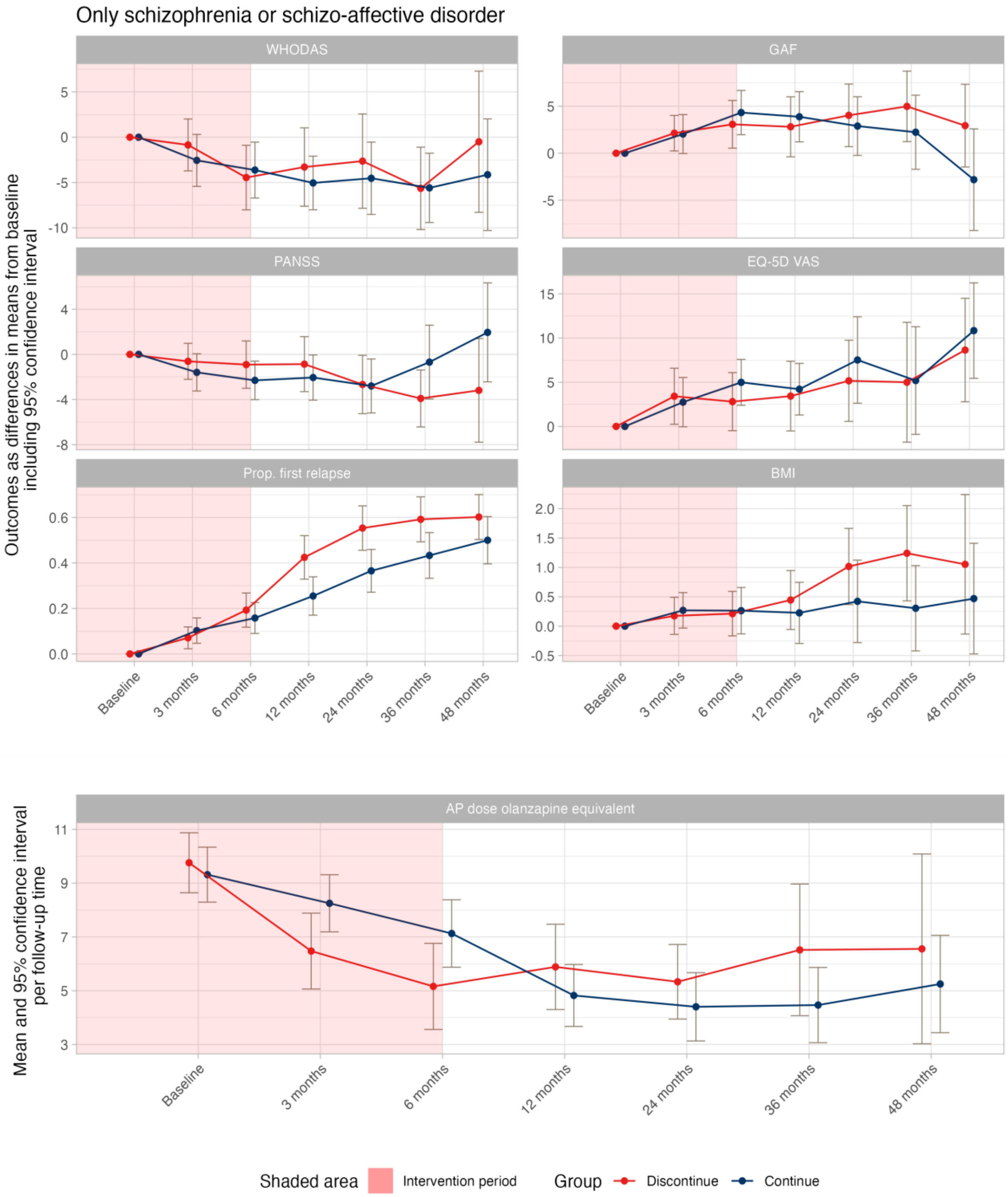

© 2025 American Medical Association. All rights reserved, including those for text and data mining, AI training, and similar technologies.
