# Supplementary material for: Early Dose Reduction or Discontinuation vs Maintenance Antipsychotics After First Psychotic Episode Remission: A Randomized Clinical Trial
Source: JAMA Psychiatry. 2025 Oct 1;83(1):68–73. doi: 10.1001/jamapsychiatry.2025.2525 (PMC12489793; doi:10.1001/jamapsychiatry.2025.2525)
Supplement: Supplement 3. — Nonauthor Collaborators [file jamapsychiatry-e252525-s003.pdf]

Supplemental Online Content: Nonauthor Collaborators
*First name, last name, and suffix (if applicable) are required and will appear in PubMed.

*Group Name(s): HAMLETT-OPHELIA consortium							
***First Name and Middle Initial(s)**	***Last Name**	***Suffix (eg, Jr, III)**	Academic Degrees	Institution	Location (city, state/province, country)	Role or Contribution, eg, chair, principal investigator	Group (if more than 1 Group listed in the byline) and/or Subgroup (eg, Steering Committee)
Iris E C	Sommer		Prof. Dr.	Center for Clinical Neuroscience and Cognition, University of	Groningen	PI, Chair, Executive Board member	
Lieuwe	de Haan		Prof. Dr.	Department of Early Psychosis, Amsterdam UMC, Academic	Amsterdam	Executive Board member	
Wim	Veling		Prof. Dr.	Department of Psychiatry, University of Groningen, University	Groningen	Executive Board member	
Jim	van Os		Prof. Dr.	Department of Psychiatry, UMC Utrecht Brain Center, University	Maastricht; Utrecht; London	Executive Board member	
Filip	Smit		Prof. Dr.	Department of Epidemiology and	Utrecht; Amsterdam	Member	
Marieke	Begemann		Dr.	Center for Clinical Neuroscience and Cognition, University of	Groningen	Executive Board member	
Sanne	Koops		Dr.	Center for Clinical Neuroscience and Cognition, University of	Groningen	Executive Board member	
Machteld	Marcelis		Prof. Dr.	Department of Psychiatry and	Maastricht; Groningen	Member	
Martijn	Kikkert		Dr.	Department of Research, Arkin Mental Health Care, Amsterdam,	Amsterdam	Executive Board member	
Nico	van Beveren		Prof. Dr.	Antes Center for Mental Health Care, Rotterdam, The Netherlands.;	Rotterdam	Executive Board member	
Nynke	Boonstra		Prof. Dr.	Department of Psychiatry, UMC Utrecht Brain Center, University	Utrecht; Leeuwarden	Executive Board member	
Bram-Sieben	Rosema		MSc	Center for Clinical Neuroscience and Cognition, University of	Leeuwarden	Executive Board member	
P. Roberto	Bakker		Dr.	Department of Psychiatry,	Groningen; Amsterdam	Researcher	
Sinan	Gülöksüz		Dr.	Department of Psychiatry and	Maastricht; New Haven	Researcher	
Joran	Lokkerbol		Dr.	Centre of Economic Evaluation &	Utrecht		
Ben	Wijnen		Dr.	Centre of Economic Evaluation & Machine Learning, Trimbos	Utrecht	Executive Board member	
Bodyl	Brand		Dr.	Department of Psychiatry,	Oxford	Researcher	
Shiral	Gangadin		Dr.	Center for Clinical Neuroscience	Groningen	Researcher	

Supplemental Online Content: Nonauthor Collaborators
*First name, last name, and suffix (if applicable) are required and will appear in PubMed.

*First Name and Middle Initial(s)	*Last Name	*Suffix (eg, Jr, III)	Academic Degrees	Institution	Location (city, state/province, country)	Role or Contribution, eg, chair, principal investigator	Group (if more than 1 Group listed in the byline) and/or Subgroup (eg, Steering Committee)
Erna	van ’t Hag		BSc	Department of Psychiatry,	Groningen	Coordinator	
Priscilla	Oomen		Dr.	Behavioural Science Institute,	Nijmegen	Researcher	
Alban	Voppel		Dr.	Center for Clinical Neuroscience	Groningen; Montréal	Researcher	
Franciska	de Beer		MSc	Center for Clinical Neuroscience	Groningen	Researcher	
Sterre	Kamphuis		MSc	Department of Psychiatry and	Maastricht	Researcher	
Iris	Hamers		MSc	Center for Clinical Neuroscience	Groningen	Researcher	
Matej	Djordjevic		MSc	Department of Psychiatry,	Groningen	Researcher	
Toon	Scheurink		MSc	Department of Biomedical Sciences,	Groningen	Researcher	
Jort	Noorman		B	Center for Clinical Neuroscience	Groningen	Coordinator	
Therese	van Amelsvoort		Prof. Dr.	Department of Psychiatry and Neuropsychology, School for	Maastricht; Heerlen	Member	
Maarten	Bak		Dr.	Department of Psychiatry and	Maastricht; Heerlen	Member	
Steven	Berendsen		Dr.	Department of Early Psychosis,	Amsterdam; Deventer;	Member	
Truus	van den Brink			Early Intervention Team, GGZ	Amersfoort	Member	
Gunnar	Faber		Dr.	Yulius, Mental Health Institute,	Dordrecht	Member	
Koen	Grootens		Prof. Dr.	Reinier van Arkel Institute for	's Hertogenbosch; Tilburg	Member	
Martin	de Jonge			Program for Psychosis & Severe	Wolfheze	Member	
Henderikus	Knegtering		Dr.	Department of Psychiatry,	Groningen	Member	
Jörg	Kurkamp		Dr.	Center for Youth with Psychosis,	Enschede	Member	
Gerdina Hendrika Maria	Pijnenborg		Prof. Dr.	Department of Psychotic Disorders, GGZ-Drenthe, Assen, The	Assen; Groningen	Member	
Anton	Staring		Dr.	Department ABC Early Psychosis,	Utrecht	Member	
Natalie	Veen		Dr.	GGZ Delfland, Delfland Institute for	Delft	Member	
Selene	Veerman		Dr.	Community Mental Health, Mental	Schagen	Member	
Sybren	Wiersma			Early Intervention Psychosis Team,	Hoofddorp	Member	
Albert	Batalla		Dr.	Department of Psychiatry, UMC	Utrecht	Member	
Ruben	Curfs			Parnassia Psychiatric Institute, The	The Hague	Member	
Jan-Jaap	Hage			GGZ Breburg, Tilburg, The	Tilburg	Member	
Ellen	Graveland			Yulius, Mental Health Institute,	Dordrecht	Member	
Joelle	Hoornaar			Antes Center for Mental Health	Rotterdam	Member	
Inge	Hobus			Center for Clinical Neuroscience	Groningen; Breda	Researcher	

Supplemental Online Content: Nonauthor Collaborators
*First name, last name, and suffix (if applicable) are required and will appear in PubMed.

***First Name and Middle Initial(s)**	***Last Name**	***Suffix (eg, Jr, III)**	Academic Degrees	Institution	Location (city, state/province, country)	Role or Contribution, eg, chair, principal investigator	Group (if more than 1 Group listed in the byline) and/or Subgroup (eg, Steering Committee)
Karin	Huizer		Dr.	Parnassia Psychiatric Institute, The Hague, The Netherlands.; Department of Psychiatry, Amsterdam UMC, Academic Medical Center, Amsterdam, The Netherlands.; Department of Pathology, Erasmus MC, Rotterdam, The Netherlands.	The Hague; Amsterdam; Rotterdam	Researcher	
