# Supplementary material for: Early Dose Reduction or Discontinuation vs Maintenance Antipsychotics After First Psychotic Episode Remission: A Randomized Clinical Trial
Source: JAMA Psychiatry. 2025 Oct 1;83(1):68–73. doi: 10.1001/jamapsychiatry.2025.2525 (PMC12489793; doi:10.1001/jamapsychiatry.2025.2525)
Supplement: Supplement 4. — Data Sharing Statement [file jamapsychiatry-e252525-s004.pdf]

Data Sharing Statement

Sommer. Early Dose Reduction or Discontinuation of Antipsychotic Medication After First Psychotic Episode Remission. *JAMA Psychiatry*. Published October 01, 2025. doi:10.1001/jamapsychiatry.2025.2525

Data
Additional Information: EudraCT number 2017-002406-12
https://www.clinicaltrialsregister.eu/ctr-search/trial/2017-002406-12/NL
Data available: No

Additional Information
Explanation for why data not available: We will not share data until our primary objectives have been published. after that data we have a data request and sharing policy in place. expected of 2027.
